# Effects of CETP inhibition with anacetrapib on metabolism of VLDL-TG and plasma apolipoproteins C-II, C-III, and E[S]

**DOI:** 10.1194/jlr.M074880

**Published:** 2017-03-17

**Authors:** John S. Millar, Michael E. Lassman, Tiffany Thomas, Rajasekhar Ramakrishnan, Patricia Jumes, Richard L. Dunbar, Emil M. deGoma, Amanda L. Baer, Wahida Karmally, Daniel S. Donovan, Hashmi Rafeek, John A. Wagner, Stephen Holleran, Joseph Obunike, Yang Liu, Soumia Aoujil, Taylor Standiford, David E. Gutstein, Henry N. Ginsberg, Daniel J. Rader, Gissette Reyes-Soffer

**Affiliations:** University of Pennsylvania,* Philadelphia, PA 19104; Merck & Co., Inc.,† Kenilworth, NJ 07033; Columbia University,§ New York, NY 10032; New York City College of Technology, CUNY,** Brooklyn, NY 11201

**Keywords:** lipoprotein metabolism, plasma lipid transfer proteins, drug therapy, kinetics, statins, cholesteryl ester transfer protein, very low density lipoprotein, triglyceride

## Abstract

Cholesteryl ester transfer protein (CETP) mediates the transfer of HDL cholesteryl esters for triglyceride (TG) in VLDL/LDL. CETP inhibition, with anacetrapib, increases HDL-cholesterol, reduces LDL-cholesterol, and lowers TG levels. This study describes the mechanisms responsible for TG lowering by examining the kinetics of VLDL-TG, apoC-II, apoC-III, and apoE. Mildly hypercholesterolemic subjects were randomized to either placebo (N = 10) or atorvastatin 20 mg/qd (N = 29) for 4 weeks (period 1) followed by 8 weeks of anacetrapib, 100 mg/qd (period 2). Following each period, subjects underwent stable isotope metabolic studies to determine the fractional catabolic rates (FCRs) and production rates (PRs) of VLDL-TG and plasma apoC-II, apoC-III, and apoE. Anacetrapib reduced the VLDL-TG pool on a statin background due to an increased VLDL-TG FCR (29%; *P* = 0.002). Despite an increased VLDL-TG FCR following anacetrapib monotherapy (41%; *P* = 0.11), the VLDL-TG pool was unchanged due to an increase in the VLDL-TG PR (39%; *P* = 0.014). apoC-II, apoC-III, and apoE pool sizes increased following anacetrapib; however, the mechanisms responsible for these changes differed by treatment group. Anacetrapib increased the VLDL-TG FCR by enhancing the lipolytic potential of VLDL, which lowered the VLDL-TG pool on atorvastatin background. There was no change in the VLDL-TG pool in subjects treated with anacetrapib monotherapy due to an accompanying increase in the VLDL-TG PR.

Cholesteryl ester transfer protein (CETP) facilitates the net exchange of cholesteryl esters (CEs) and triglycerides (TGs) between HDL particles and apoB-containing lipoproteins (1–3). Inhibition of CETP reduces CE and TG exchange among lipoproteins, which has effects on the lipid composition of lipoproteins as well as their metabolism (4–6). CETP inhibition is associated with increased levels of HDL-cholesterol (HDL-C) and reduced levels of LDL-cholesterol (LDL-C) and apoB (7). Potent CETP inhibition also modestly lowers TG levels; anacetrapib (100 mg/day) reduced TG by 6.8% (8) and evacetrapib (100 mg/day) by up to 7.9% (9) in dyslipidemic patients on background statin therapy. Large cohort studies have shown associations between genetic variation in *CETP* and CVD risk. Results from these large studies have identified polymorphisms that result in reduced CETP activity and are associated with reduced CVD risk (7). In addition, studies in animal models have shown beneficial effects of CETP inhibition on reducing the development of atherosclerosis (7). While these findings initially made CETP an attractive target for reducing CVD risk, subsequent studies with CETP inhibitors have shown an apparent lack of efficacy or harm due to off-target effects, leading to increased uncertainty around the hypothesis that CETP reduces CVD risk (7, 10, 11).

Anacetrapib is a CETP inhibitor that is currently being evaluated in a phase 3 trial to determine its effects on cardiovascular protection when added to a statin (12). We have previously reported that CETP inhibition has the effects of enhancing VLDL and LDL apoB clearance while reducing the clearance of HDL apoA-I (4, 5), changes that are thought to reduce atherosclerotic risk. In the case of VLDL apoB, we speculated that CETP inhibition resulted in the formation of a TG-enriched VLDL particle that was an optimal substrate for lipoprotein lipase-mediated lipolysis and, hence, the increase in VLDL apoB fractional catabolic rate (FCR). If this was indeed the case, we hypoth­esized that CETP inhibition should enhance clearance of TG from VLDL over and above what is seen during the baseline period. The current study was conducted to determine the effects of CETP inhibition with anacetrapib on the production and clearance of VLDL-TG. We also measured the metabolism of apoC-II, apoC-III, and apoE, three proteins that affect VLDL lipolysis and clearance from the circulation.

## MATERIALS AND METHODS

### Study subjects/design

Thirty-nine mildly hypercholesterolemic subjects were enrolled at Columbia University Medical Center and the University of Pennsylvania. A detailed study design has been reported previously (4) (ClinicalTrials.gov identifier NCT00990808; MK0859 PN026). This study protocol is included in the supplemental materials. Subject characteristics at screening are shown in supplemental Table S1. Subjects were randomized to either panel A (anacetrapib plus statin background treatment) or panel B (anacetrapib monotherapy plus background of placebo) in a 3:1 ratio according to a computer-generated allocation schedule stratified by LDL-C levels <160 mg/dl or ≥160 mg/dl to ensure a balanced distribution of these subjects across panels. Each panel consisted of two treatment periods with no wash-out period separating the treatments within each panel.

In period 1, subjects received background treatment with placebo or atorvastatin 20 mg daily, for a minimum of 4 weeks. In period 2, subjects added anacetrapib 100 mg/day once daily for 8 weeks (maximum of 9 weeks) to their existing background treatment.

At the end of each treatment period subjects underwent a lipoprotein kinetic study performed with bolus injections of [5,5,5-^2^H_3_]leucine (9–10 μmol/kg body weight) and [1,1,2,3,3-^2^H_5_]glycerol (100 μmol/kg body weight) and primed-constant infusion of [5,5,5-^2^H_3_]leucine (9–10 μmol/kg body weight prime, 9–10 μmol/kg body weight/h infusion) (isotopes from Cambridge Isotope Laboratories, Cambridge, MA) over a 15 h period under continuous feeding conditions. Blood samples were collected at 0 (pre-bolus), 20, and 40 min, and at 1, 2, 4, 6, 8, 10, 12, 14, 15, 15.5, 16, 18, 21, 24, and 48 h post-bolus to determine apolipoprotein and TG kinetics.

### Biochemical and immunologic assays

Blood for biochemical measurements was collected at the end of each period following a 12 h fast. Total cholesterol (TC), TG, and HDL-C in fasting plasma as well as TG and cholesterol levels in isolated VLDL (via density ultracentrifugation) from plasma obtained during the kinetic study were measured enzymatically on a Cobas Fara II autoanalyzer (Roche Diagnostic Systems, Inc., Basel, Switzerland) using Sigma reagents (Sigma Chemical Co., St. Louis, MO). LDL-C levels were determined using the Friedewald formula. Plasma apoC-II, apoC-III, and apoE concentrations for pool size determination were measured in samples collected during each kinetic study using LC-MS/MS (13). apoC-III in VLDL and HDL fractions isolated by ultracentrifugation was determined using the apoC3 human ELISA kit (ab154131; Abcam, Cambridge, MA).

### VLDL-TG kinetics

VLDL-TG enrichment with [1,1,2,3,3-^2^H_5_]glycerol was measured by the Metabolic Tracer Resource at the University of Pennsylvania and at the Irving Institute for Clinical and Translational Research core resource laboratory at Columbia University Medical Center (14). VLDL (d < 1.006 g/ml) was isolated by ultracentrifugation from blood samples collected throughout the kinetic study. VLDL lipids were extracted using chloroform:methanol and TG isolated using either zeolite or BondElut NH_2_ columns. TG extracts were saponified and the liberated glycerol derivatized (15) and analyzed by GC-MS. D_5_-glycerol enrichments were determined from the ratio of M+5/M+0 ions using standards of known enrichment. Kinetic parameters were individually determined by fitting the stable isotope-labeled glycerol enrichment data to a multicompartmental model using a weighted least-squares approach with WinSAAM version 3.0.7. The multicompartmental model was identical to that previously reported for apoB (4). Transfer rates between compartments were constrained to values determined for apoB, while rates for direct clearance were variable (16). The hepatic TG precursor was represented by the plasma [1,1,2,3,3-^2^H_5_]glycerol enrichment or, if unavailable, a reference plasma glycerol model (16). The FCR of VLDL-TG was calculated from kinetic parameters as the fraction of TG cleared from plasma per day. The production rate (PR) for VLDL-TG was calculated as the product of the FCR and the plasma pool size, which was calculated as the product of the average VLDL-TG concentration, measured at a minimum of three time points during the metabolic study, and the plasma volume, assumed to be 4.5% of body weight.

### apoC-II, apoC-III, and apoE kinetics

apoC-II, apoC-III and apoE analyses were prepared for LC-MS using a method published previously (17) but modified to reduce trypsin volume requirements and to achieve maximum digestion efficiency while reducing the overall cost of analysis (18). Briefly, 20 μl of plasma was diluted and digested with trypsin overnight, prior to analysis performed by ultra-performance LC combined with a triple quadrupole MS using multiple reaction monitoring to measure isotope enrichment. apoC-II, apoC-III, and apoE kinetic parameters were individually determined by fitting the stable isotope-labeled leucine tracer data to a multicompartmental model using a weighted least-squares approach using WinSAAM version 3.0.7. The multicompartmental model consisted of three compartments: a hepatic precursor, a synthetic delay, and plasma protein (apoC-II, apoC-III, or apoE). The hepatic precursor was represented by the plasma [5,5,5-^2^H_3_]leucine enrichment. The FCR of each protein was calculated from kinetic parameters as the fraction of protein cleared from plasma per day. The PR for each protein was calculated as the product of the FCR and the plasma pool size measured at a minimum of three time points during the metabolic study, and the plasma volume, assumed to be 4.5% of body weight.

### Statistics

All statistical analyses were conducted using SAS^®^ software (SAS Institute Inc., Cary, NC). Analysis was performed on log-scale and the estimates obtained were back-transformed using the formula 100 × [exp (estimate) − 1] to yield point estimates, 95% CIs and between-treatment *P* values (two-tailed) for the true percent change from treatment in period 1. Data normality was first assessed. For normally distributed data, linear mixed effects models containing fixed effects for panel and treatment within panel, and random effect for subject within panel were used to assess percent change from period 1 treatment. Geometric mean and percent coefficient of variation (%CV) were also provided for each treatment. For nonnormally distributed data, median and inter­quartile ranges were reported for individual treatment periods. Hodges-Lehmann estimates based on Wilcoxon signed rank test and corresponding *P* values were reported for treatment period differences (period 2 – period 1). The endpoints and comparisons in this work were exploratory endpoints and were tested at the 0.05 level and were not subject to multiplicity adjustment. All available data were included and no data were excluded from analysis.

### Study approval

The study was conducted in accordance with the Principles of Good Clinical Practice and was approved by the Institutional Review Boards at Columbia University Medical Center and the University of Pennsylvania. All study subjects provided written informed consent.

## RESULTS

The baseline characteristics of the subjects participating in this study have been reported previously (4). Briefly, 39 subjects completed the study. Subjects completing the study were predominantly male (67%) with a mean age of 48 years and a BMI of 30 ± 5 kg/m^2^. At screening, subjects had a mean TC level of 214 mg/dl, median TG level of 118 mg/dl, and mean LDL-C level of 137 mg/dl. Following treatment with atorvastatin (panel A), subjects had a mean TC level of 163 mg/dl, mean TG level of 89 mg/dl, and median LDL-C level of 90 mg/dl, while subjects on placebo (panel B) had mean TC level of 209 mg/dl, mean TG level of 121 mg/dl, and median LDL-C level of 134 mg/dl (**Table 1**).

**TABLE 1.** Baseline lipid data following background statin (panel A) or placebo (panel B) treatment

Characteristic	Panel A (N = 29)	Panel B (N = 10)	All Subjects (N = 39)
TC (mg/dl)	163	209	184
Geometric mean (% CV)	(13)	(17)	(17)
TGs (mg/dl)	89	121	104
Geometric mean (% CV)	(38)	(59)	(44)
LDL-C (mg/dl)	90	134	93
Median (IQR)	(16)	(23)	(39)
HDL-C (mg/dl)	48	43	48
Median (IQR)	(19)	(20)	(20)
apoC-II (mg/dl)	4.4	5.2	4.4
Median (IQR)	(1.3)	(3.1)	(1.5)
apoC-III (mg/dl)	7.4	8.4	7.9
Geometric mean (% CV)	(40.8)	(35.2)	(39.4)
apoE (mg/dl)	3.3	4.4	3.7
Median (IQR)	(0.8)	(0.8)	(0.8)

### VLDL-TG response to anacetrapib monotherapy or on background statin treatment

Anacetrapib treatment significantly reduced the VLDL-TG pool size by 14% in the subjects treated on an atorvastatin background. This was due to a significant 29% increase in the VLDL-TG FCR (**Table 2**). The increase in the VLDL-TG FCR was somewhat offset by a trend toward an increase in the VLDL-TG PR of 14%. In contrast, there was no significant change in the VLDL-TG pool size in the subjects treated with anacetrapib monotherapy. The lack of change in the VLDL-TG pool size in the anacetrapib monotherapy group was due to competing mechanisms of increased VLDL-TG FCR (40%, *P* = 0.11) and increased VLDL-TG PR (39%, *P* = 0.01). The ratio of VLDL-TG PR/VLDL apoB100 PR provides a measure of the TG content of newly secreted VLDL. The VLDL-TG PR/VLDL apoB100 PR ratio increased by 18% in subjects treated on an atorvastatin background. In contrast, subjects treated with anacetrapib monotherapy showed no significant change in the VLDL-TG PR/VLDL apoB100 PR ratio.

**TABLE 2.** VLDL-TG kinetics in subjects treated with anacetrapib in combination with atorvastatin (panel A) or anacetrapib alone (panel B)

	Panel A (N = 28)				Panel B (N = 10)				All Subjects (N = 38)			
	Period 1 (ATV)	Period 2 (ANA+ATV)	Percent Change from Period 1 (95% CI)	*P*	Period 1 (PBO)	Period 2 (ANA)	Percent Change from Period 1 (95% CI)	*P*	Period 1	Period 2	Percent Change from Period 1 (95% CI)	*P*
VLDL-TG PS (mg)	3,778 (56.5)	3,235 (58.9)	−14.4 (−23.1, −4.6)	0.006	3,740 (78.9)	3,696 (64.6)	−1.2 (−17.5, 18.3)	0.894	3,759 (61)	3,458 (60)	−8.0 (−17.2, 2.2)	0.115
VLDL-TG FCR*^a^* (pools/day)	7.32 (3.3)	10.45 (6.04)	29.4 (5.9, 52.8)	0.002	5.64 (3.4)	7.77 (2.2)	40.7 (−15.0, 64.4)	0.105	6.78 (3.75)	8.54 (5.9)	31.4 (10.8, 52.6)	<0.001
VLDL-TG PR (mg/kg/day)	324.6 (57.0)	370.2 (54.9)	14.1 (−2.1, 32.8)	0.089	306.5 (53.0)	425.1 (72.7)	38.7 (7.4, 79.0)	0.014	315.4 (55.2)	396.7 (59.1)	25.8 (8.4, 45.9)	0.004
VLDL-TG PR/VLDL apoB PR (mg/mg)	21.5 (19.0)	23.4 (14.9)	18.1 (0.1, 38.5)	0.048	19.6 (20.5)	15.0 (39.4)	−2.5 (−31.5, 40.5)	0.846	20.4 (18.7)	23.1 (23.9)	11.3 (−3.0, 28.1)	0.136

Mixed model analysis performed on log scale. Geometric mean (% CV) displayed under period 1 and period 2. Percent change from period 1 and corresponding 95% CI calculated using 100 × (GMR − 1). ATV, atorvastatin; ANA, anacetrapib; PBO, placebo.

a Nonparametric method used. Median (IQR) on raw scale displayed under period 1 and period 2. Hodges-Lehmann estimate (95% CI) back transformed from log scale displayed under percent change from period 1. *P* value from Wilcoxon signed rank test.

### Plasma apoC-II, apoC-III, and apoE kinetic response to anacetrapib monotherapy or anacetrapib plus background statin treatment

The plasma apoE pool size increased by 22% in subjects on anacetrapib plus statin (*P* = 0.015). This was due to a 21% increase in the apoE PR (*P* = 0.009) with no significant change in the apoE FCR (**Table 3**). In subjects treated with anacetrapib monotherapy, there was no significant change in the apoE pool size. Consistent with the lack of change in the apoE pool size, there were no significant changes in either the apoE FCR or PR in the anacetrapib monotherapy group.

**TABLE 3.** Plasma apoC-II, apoC-III, and apoE kinetics in subjects treated with anacetrapib in combination with atorvastatin (panel A) or anacetrapib alone (panel B)

	Panel A (N = 29)				Panel B (N = 10)				All Subjects (N = 39)			
	Period 1 (ATV)	Period 2 (ANA+ATV)	Percent Change from Period 1 (95% CI)	*P*	Period 1 (PBO)	Period 2 (ANA)	Percent Change from Period 1 (95% CI)	*P*	Period 1	Period 2	Percent Change from Period 1 (95% CI)	*P*
apoC-II PS (mg)	193 (42.1)	220 (40.2)	13.9 (5.0, 23.5)	0.002	203 (40.4)	233 (43.7)	14.8 (0.0, 31.8)	0.050	198 (41.2)	226 (40.6)	14.4 (5.6, 23.9)	0.002
apoC-II FCR (pools/day)	0.63 (25.4)	0.57 (35.1)	−9.8 (−17.7, −1.2)	0.028	0.56 (34.6)	0.53 (32.5)	−5.6 (−19.3, 10.2)	0.452	0.59 (28.0)	0.55 (34.1)	−7.8 (−15.7, 0.9)	0.077
apoC-II PR (mg/kg/day)	1.39 (32.5)	1.43 (38.1)	2.8 (−8.7, 15.7)	0.641	1.45 (389.0)	1.57 (28.0)	8.6 (−11.2, 32.8)	0.410	1.42 (33.7)	1.50 (35.7)	5.7 (−6.0, 18.7)	0.345
apoC-III PS*^a^* (mg)	380 (34.0)	498 (48.2)	31.1 (6.5, 61.4)	0.012	287 (34.9)	504.7 (45.3)	75.8 (23.3, 150.5)	0.003	330 (36.3)	502 (46.8)	51.8 (23.6, 86.4)	<0.001
apoC-III FCR*^a^* (pools/day)	0.83 (0.3)	0.75 (0.4)	−7.5 (−22.6, 7.1)	0.465	0.86 (0.3)	0.50 (0.4)	−35.0 (−69.9, −19.6)	0.027	0.83 (0.3)	0.70 (0.5)	−14.7 (−28.0, −1.0)	0.078
apoC-III PR (mg/kg/day)	3.62 (1.7)	4.15 (1.9)	20.0 (−8.3, 51.6)	0.108	3.07 (1.4)	3.91 (3.0)	27.5 (−42.3, 46.9)	0.557	3.44 (1.9)	4.15 (2.2)	20.4 (−3.7, 42.9)	0.065
apoE PS (mg)	154 (31.9)	188 (39.5)	22.0 (4.2, 42.9)	0.015	145 (30.3)	168 (31.7)	15.2 (−11.9, 50.6)	0.293	150 (31.2)	178 (37.6)	18.6 (1.5, 38.5)	0.033
apoE FCR (pools/day)	3.71 (25.3)	3.67 (46.6)	−1.0 (−18.4, 20.0)	0.914	3.98 (20.1)	3.57 (37.2)	−10.2 (−35.3, 24.7)	0.510	3.84 (24.0)	3.62 (43.8)	−5.7 (−22.1, 14.0)	0.533
apoE PR (mg/kg/day)	6.55 (21.7)	7.91 (28.2)	20.8 (5.0, 38.8)	0.009	7.34 (17.2)	7.63 (32.8)	3.9 (−18.1, 31.7)	0.746	6.93 (21.1)	7.77 (29.0)	12.0 (−2.4, 28.5)	0.103

Mixed model analysis performed on log scale. Geometric mean (% CV) displayed under period 1 and period 2. Percent change from period 1 and corresponding 95% CI calculated using 100 × (GMR − 1). ATV, atorvastatin; ANA, anacetrapib; PBO, placebo.

a Nonparametric method used. Median (IQR) on raw scale displayed under period 1 and period 2. Hodges-Lehmann estimate (95% CI) back transformed from log scale displayed under percent change from period 1. *P* value from Wilcoxon signed rank test.

The apoC-II PS increased by 14% in subjects treated with anacetrapib plus statin (*P* = 0.002). This was due to a 10% reduction in the apoC-II FCR (*P* = 0.028) with no change in the apoC-II PR (Table 3). In subjects treated with anacetrapib monotherapy there was a 15% increase in the apoC-II pool size (*P* = 0.05). Despite the increase in apoC-II pool size, no detectible changes in either the apoC-II FCR or PR were observed.

The apoC-III PS increased by 31% in response to anacetrapib plus statin (*P* = 0.012). There was no significant change in the apoC-III FCR, while the apoC-III PR trended in a positive direction (+20%, *P* = 0.11). In subjects treated with anacetrapib monotherapy, there was a 76% increase in the apoC-III pool size (*P* = 0.003). The increase was associated with a significant 35% reduction in the apoC-III FCR (*P* = 0.027) and a nonsignificant increase in the apoC-III PR.

Because an increase in plasma apoC-III is typically accompanied by reduced clearance of VLDL-TG, we examined the distribution of apoC-III among the VLDL and HDL lipoprotein fractions isolated by ultracentrifugation. In both treatment panels there were significant reductions in the amount of apoC-III in the VLDL fraction (**Table 4**). This was accompanied by a significant increase in the amount of apoC-III in the HDL fraction.

**TABLE 4.** apoC-III in VLDL and HDL during the atorvastatin or placebo run-in period and following addition of anacetrapib

	Panel A (N = 26)				Panel B (N = 9)				All Subjects (N = 35)			
	Period 1 (ATV)	Period 2 (ANA+ATV)	Percent Change from Period 1 (95% CI)	*P*	Period 1 (PBO)	Period 2 (ANA)	Percent Change from Period 1 (95% CI)	*P*	Period 1	Period 2	Percent Change from Period 1 (95% CI)	*P*
apoC-III in VLDL*^a^* (μg/ml)	37.80 (27.60)	16.00 (23.20)	−51.49 (−66.16, −31.09)	<.001	39.60 (40.80)	12.00 (6.40)	−70.77 (−90.59, −25.19)	0.020	39.60 (29.20)	14.00 (22.80)	−56.00 (−69.15, −40.56)	<0.001
apoC-III in HDL*^b^* (μg/ml)	79.59 (49.35)	97.50 (32.01)	22.50 (4.31, 43.86)	0.015	72.48 (71.33)	107.73 (33.65)	48.64 (11.95, 97.35)	0.008	75.95 (54.08)	102.49 (32.24)	34.94 (14.65, 58.82)	<0.001

a Nonparametric method used. Median (IQR) on raw scale displayed under period 1 and period 2. Hodges-Lehmann estimate (95% CI) back transformed from log scale displayed under percent change from period 1. *P* value from Wilcoxon signed rank test. ATV, atorvastatin; ANA, anacetrapib; PBO, placebo.

b Mixed model analysis performed on log scale. Geometric mean (% CV) displayed under period 1 and period 2. Percent change from period 1 and corresponding 95% CI calculated using 100 × (GMR − 1); (panel A, N = 28; panel B, N = 9; all subjects, N = 37).

## DISCUSSION

CETP mediates the net exchange of TG on VLDL for CE on HDL. While CETP inhibition might be expected to increase plasma TG levels due to inhibition of TG transfer out of VLDL (6), plasma TG levels have been reported to be reduced following potent CETP inhibition with anacetrapib (8). A reduction in plasma TG levels seen in response to CETP inhibition may be due to a reduced VLDL-TG PR or increased clearance of VLDL-TG. In the latter case, increases in either lipolysis or direct removal of TGs in VLDL remnants by the liver would both have the effect of lowering VLDL-TG levels. The current study was conducted to assess the mechanism responsible for changes in VLDL-TG in response to CETP inhibition with anacetrapib.

We found that CETP inhibition with anacetrapib enhanced fractional catabolism of VLDL-TG both on background statin treatment and when anacetrapib was given as monotherapy. On the background of statin treatment, a trend toward an increase in the PR of VLDL-TG was far outweighed by the increase in FCR, and the VLDL-TG pool size fell significantly. However, in the anacetrapib monotherapy group, the increase in VLDL-TG PR was of a similar magnitude to the increase in VLDL-TG FCR, and the VLDL-TG pool size, therefore, did not change. The reason for an increase in the FCR of VLDL-TG is not apparent, but could be due to a number of factors. First, the decrease in the transfer of VLDL-TG to HDL may result in larger TG-rich VLDLs, which are better substrates for lipoprotein lipase than smaller particles (19). Second, CETP inhibition may lead to an increase in lipoprotein lipase mass with greater lipolysis of VLDL-TGs. Third, the reduced transfer of VLDL-TG to HDL in response to CETP inhibition may result in a redistribution of exchangeable apolipoproteins on VLDL and HDL, which could enhance the in vivo activity of lipoprotein lipase or facilitate direct hepatic removal of VLDL remnants.

The ratio of the VLDL-TG and apoB PRs can be used as a measure of the TG content of newly secreted VLDL particles. Our results indicated that there was a modest, but significant, increase in the TG content of new secreted VLDL in response to CETP inhibition with anacetrapib on the background of a statin, but not with anacetrapib monotherapy. This indicates that there is a change in the size of newly secreted VLDL following treatment with anacetrapib and would suggest that a change in VLDL size may contribute to the increased clearance rate of VLDL-TG that we measured in subjects on a background of statin treatment, but not with anacetrapib monotherapy. Mice treated with anacetrapib showed no change in the VLDL-TG or apoB PRs, indicating no changes in number or TG content of newly secreted VLDL (20). We previously measured lipoprotein lipase mass and activity and found that there were no changes in either measure in response to treatment with anacetrapib (4). This indicates that a change in lipoprotein lipase mass or activity is not responsible for the increased clearance rate of VLDL-TG that we measured. Previous reports have shown that the TG:cholesterol ratio of VLDL was increased following treatment with anacetrapib (4, 21). This suggests that, while the size of VLDL is unchanged in response to anacetrapib treatment, circulating VLDL is relatively enriched with TG and depleted of cholesterol. Cholesterol has been reported to lower the activity of lipoprotein lipase against TG-enriched emulsions (22). This is thought to be due to effects of cholesterol on limiting availability of TG on the outer phospholipid layer of the VLDL particle surface (23), which leads to reduced TG lipolysis (24). The cholesterol content of VLDL can also influence particle affinity for apolipoproteins that regulate lipoprotein lipase activity (22), as well as the particle affinity of lipoprotein lipase (25).

VLDL-TG metabolism is regulated by both the activity of lipoprotein lipase, which is controlled by a number of exchangeable apolipoproteins, including apoC-II and C-III, and whole particle VLDL uptake by lipoprotein receptors, controlled, in part, by apoE and apoC-III. We measured the changes in these apolipoproteins to determine their role in potential downstream effects on VLDL-TG metabolism. There was an increase in the apoE pool size in subjects treated with anacetrapib on a background of atorvastatin, but not when used as a monotherapy. Bisgaier et al. (26) conducted in vitro studies examining the effect of CETP inhibition on the redistribution of apoE among VLDL and HDL. They found that inhibition of CETP with a neutralizing antibody promoted the redistribution of apoE from HDL to lipoproteins in a size range similar to larger apoB-lipoproteins. Such a change would be expected to increase whole particle VLDL clearance. We did not detect a change in VLDL-apoB clearance, as shown by the lack of effects on the conversion of VLDL-apoB to LDL-apoB after anacetrapib treatment (4). This suggests that anacetrapib treatment primarily increases VLDL-TG lipolysis, but not VLDL-apoB clearance, when given on the background of a statin.

Two apolipoproteins known to regulate VLDL lipolysis by lipoprotein lipase are apoC-II and apoC-III, which activate and inhibit lipoprotein lipase, respectively. Anacetrapib treatment increased apoC-II and apoC-III pool sizes both when used as a monotherapy and on an atorvastatin background. The increase of the apoC-II pool is unlikely to promote further activity of lipoprotein lipase beyond what is observed during the baseline period. Minimal amounts of apoC-II are required to activate lipoprotein lipase beyond which there is no apparent increase in activity (27, 28). Shachter et al. (29) have shown that overexpression of apoC-II in mice results in hypertriglyceridemia due to displacement of apoE on VLDL. We saw no effect of CETP inhibition with anacetrapib on direct clearance of VLDL, suggesting that there was no change in the relative amounts of apoE and apoC-II on VLDL following anacetrapib treatment. Increases in the apoC-III pool size might be expected to result in an increase in plasma TG levels by inhibiting both VLDL clearance and lipoprotein lipase. In our study, the opposite effect on plasma TG levels was observed. We found that this was due to a shift of apoC-III particles from VLDL to HDL in response to anacetrapib treatment, an effect likely due to an increase in the surface area of HDL. A similar redistribution in apoC-III from VLDL to HDL was seen by Krauss et al. (21) in response to anacetrapib treatment. Redistribution of apoC-III from VLDL to HDL reduces the inhibitory effect of apoC-III on lipolysis of VLDL-TG, consistent with what we observed in the current study. Krauss et al. (21) reported that the increase in HDL apoC-III following anacetrapib treatment was greater than 100%, whereas the increase in HDL apoE was ∼50%. This indicates that there may be a greater reduction in apoC-III than apoE in VLDL, which may enhance lipolysis of TG on VLDL. While anacetrapib has not been tested in patients with hypertriglyceridemia, a similar TG-lowering effect would be expected in patients with elevated TG levels resulting from excessive apoC-III.

In an effort to further understand the regulation of TG metabolism following anacetrapib treatment, we studied the kinetics of apoC-II, apoC-III, and apoE. Our findings varied between the groups of statin background and monotherapy. In subjects treated with anacetrapib on a statin background, apoC-II, apoC-III, and apoE pool sizes were all significantly increased. Because these apolipoproteins exchange between VLDL and HDL, it might be expected that the increase in pool sizes of these constituents would be a result of reduced clearance from plasma, similar to what was observed for HDL apoA-I (5). Indeed, we did find that the change in apoC-II pool size in this group was due to a reduction in the FCR. However, the rise in both the apoC-III and apoE pool sizes was due to an increased PR. The increase in the apoE PR is consistent with an increase in TG-rich lipoprotein apoE production that we reported in statin-treated subjects in response to the CETP inhibitor, torcetrapib (30). An increase in apoE and apoC-III production could be due to a direct effect of anacetrapib on *APOE* and *APOC3* expression in the liver or could be secondary to effects on expression due to enhanced delivery of lipids from apoB-containing lipoproteins and HDL to liver. In subjects treated with anacetrapib monotherapy, we observed a significant increase in the apoC-II and apoC-III pool sizes. In contrast to what was seen in statin-treated subjects, the increase in the apoC-III pool size in the monotherapy group was due to a reduction in the apoC-III FCR. It should be noted that in both the statin-treated and monotherapy groups, while not always significant, there were reductions in the apoC-III FCR and an increase in the PR, so both mechanisms may be contributing to the increase in apoC-III pool size following anacetrapib treatment.

The clinical implications of these findings are not readily apparent. CETP inhibition with other candidates did not result in cardiovascular benefit when compared with statin treatment (10, 11, 31) for reasons that have been discussed (32). Anacetrapib is currently being evaluated in the REVEAL study to determine its effect on cardiovascular risk. If anacetrapib is found to be successful in reducing cardiovascular risk, further studies will be needed to determine whether the benefit results from reduction in apoB-containing lipoproteins, TG, an increase in HDL, or some combination of beneficial changes.

In summary, anacetrapib treatment increases the FCR of VLDL-TG, likely by increasing the TG:cholesterol ratio on VLDL, and possibly the size of newly secreted VLDL, thus enhancing the lipolytic potential of VLDL. We found no evidence for an increase in apoC-III resulting in impaired VLDL-TG clearance, most likely due to a shift in apoC-III from VLDL to HDL. The increase in the VLDL-TG FCR was associated with decreases in VLDL-TG levels when anacetrapib was added to atorvastatin treatment. There was no change, however, in VLDL-TG in subjects treated with anacetrapib monotherapy due to an accompanying similar increase in the VLDL-TG PR.

## Supplementary Material

Supplemental Data
