# Supplementary material for: Effects of CETP inhibition with anacetrapib on metabolism of VLDL-TG and plasma apolipoproteins C-II, C-III, and E
Source: J Lipid Res. 2017 Mar 17;58(6):1214–20. doi: 10.1194/jlr.M074880 (PMC5454510; doi:10.1194/jlr.M074880)
Supplement: Supplemental Data [file supp_58_6_1214__index.html]

Effects of CETP Inhibition with Anacetrapib on Metabolism of VLDL TG and Plasma Apolipoproteins C-II, C-III, and E — Effects of CETP inhibition with anacetrapib on metabolism of VLDL-TG and plasma apolipoproteins C-II, C-III, and E — Supplemental Data 

# Effects of CETP inhibition with anacetrapib on metabolism of VLDL-TG and plasma apolipoproteins C-II, C-III, and E

## Supplemental Data

- TG Kinetics (.pdf, 9 KB) - Characteristics of study subjects at screening. Values for continuous variables are mean ? standard deviation except triglyceride which is median (interquartile range).
- Supplemental Materials (.pdf, 2.2 MB) - Redacted Protocol for manuscript
