# Supplementary material for: Effects of CETP inhibition with anacetrapib on metabolism of VLDL-TG and plasma apolipoproteins C-II, C-III, and E
Source: J Lipid Res. 2017 Mar 17;58(6):1214–20. doi: 10.1194/jlr.M074880 (PMC5454510; doi:10.1194/jlr.M074880)
Supplement: Supplemental Data [file 10.1194_M074880_jlr.M074880-1.pdf]

Supplemental Table I. Characteristics of study subjects at screening. Values for continuous variables are mean ± standard deviation except triglyceride which is median (interquartile range).

Characteristic	All Subjects (n=39)
Age (years)	48 ± 10
Sex (M/F)	25/13
Race (Caucasian/Black/Other)	23/13/3
Weight (kg)	87 ± 17
Height (cm)	171 ± 8.8
BMI (kg/m^2)	30 ± 5
Systolic Blood Pressure (mm Hg)	123.0 ± 10.7
Diastolic Blood Pressure (mm Hg)	77.5 ± 9.2
Total Cholesterol (mg/dL)	214 ± 30
LDL Cholesterol (mg/dL)	137 ± 25
HDL Cholesterol (mg/dL)	49 ± 14
Triglyceride (mg/dL)	118 (84, 146)
