# Supplementary material for: Effects of CETP inhibition with anacetrapib on metabolism of VLDL-TG and plasma apolipoproteins C-II, C-III, and E
Source: J Lipid Res. 2017 Mar 17;58(6):1214–20. doi: 10.1194/jlr.M074880 (PMC5454510; doi:10.1194/jlr.M074880)
Supplement: Supplemental Data [file 10.1194_M074880_jlr.M074880-2.pdf]

026-03

A Multiple Dose Study to Investigate the Effect of MK-0859 on Lipoprotein Metabolism When Added to Ongoing Statin Therapy in Dyslipidemic Patients

Product: MK-0859
Protocol/Amendment No.: 026-03

THIS PROTOCOL AND ALL OF THE INFORMATION RELATING TO IT ARE CONFIDENTIAL AND PROPRIETARY PROPERTY OF MERCK & CO., INC., WHITEHOUSE STATION, NJ, U.S.A.

THIS PROTOCOL REPLACES THE ORIGINAL PROTOCOL AND ANY SUBSEQUENT AMENDMENTS AND SHOULD BE SIGNED BY ALL INVESTIGATORS SIGNING THE ORIGINAL PROTOCOL.

SPONSOR:

Merck & Co., Inc. (hereafter referred to as the **SPONSOR**)
One Merck Drive
P.O. Box 100
Whitehouse Station, NJ, 08889-0100, U.S.A.

Protocol-specific Sponsor Contact information can be found in the Administrative Binder.

TITLE:

A Multiple Dose Study to Investigate the Effect of MK-0859 on Lipoprotein Metabolism When Added to Ongoing Statin Therapy in Dyslipidemic Patients

INVESTIGATOR:

PRIMARY:

CLINICAL PHASE: I

US IND NUMBER: 73,290

SITE:

INSTITUTIONAL REVIEW BOARD/ETHICS REVIEW COMMITTEE:

Product: MK-0859 1
Protocol/Amendment No.: 026-03

SUMMARY OF CHANGES

PRIMARY REASON FOR THIS AMENDMENT:

[redacted]

[redacted]

[redacted]

[redacted]

[redacted]

[redacted]

- [redacted]

CCI [redacted]

[redacted]

- [redacted]

  [redacted]

- [redacted]

  [redacted]

PROTOCOL

A Multiple Dose Study to Investigate the Effect of MK-0859 on Lipoprotein Metabolism When Added to Ongoing Statin Therapy in Dyslipidemic Patients

TABLE OF CONTENTS

Contents	Application Starting Page
Summary of Changes	3
PRIMARY REASON FOR THIS AMENDMENT:	3
OTHER CHANGES INCLUDED IN THE AMENDMENT:	3
1. SUMMARY	10
1.1 Title	10
1.2 Indication	10
1.3 Summary of Rationale	10
1.4 Summary of Study Design	10
1.5 Sample	12
1.6 Dosage/Dosage Form, Route, and Dose Regimen	12
1.7 Study Flow Chart	14
2. CORE PROTOCOL	17
2.1 Objectives and Hypotheses	17
2.1.1 Primary	17
2.1.2 Secondary	17
2.1.3 Tertiary	17
2.1.4 Exploratory	18
2.2 Patient Inclusion Criteria	19
2.3 Patient Exclusion Criteria	21
2.4 Study Design and Duration	24
2.4.1 Summary of Study Design	24
2.4.2 Treatment Plan	25
2.5 List of Efficacy/Pharmacokinetic/Immunogenicity, etc., Measurements	26

TABLE OF CONTENTS (CONT.)

Contents	Application Starting Page
2.5.1 Pharmacodynamic Measurements	26
2.5.2 Pharmacokinetic Measurements (for archive only)	26
2.6 List of Safety Measurements	27
2.7 Data Analysis Summary	27
3. PROTOCOL DETAILS	29
3.1 Rationale	29
3.1.1 Rationale for This Study	29
3.1.2 Rationale for Dose Regimen and Endpoints	33
3.1.3 Rationale for Subject/Patient Genetic Sample Collection	34
3.2 Study Procedures	34
3.2.1 Study Restrictions	34
3.2.1.1 Concomitant Medication	34
3.2.1.2 Diet	35
3.2.1.3 Alcohol/Caffeine/Smoking/Activity	36
3.2.2 Procedures	37
3.2.2.1 Prestudy (Screening) (Visit 1)	40
3.2.2.2 Washout Period (Visit 2)	41
3.2.2.3 Period 1: Day 1 Randomization (Visit 3)	41
3.2.2.4 Poststudy	45
3.2.2.5 Volume of Blood Drawn During the Study	45
3.2.3 Procedures	45
3.2.3.1 Informed Consent	45
3.2.3.1.1 General Informed Consent	45
3.2.3.1.2 Consent and Collection of Specimens for Genetic Analysis	46
3.2.3.2 Assignment of Baseline Number/Screening	46
3.2.3.3 Randomization/Allocation	46

TABLE OF CONTENTS (CONT.)

Contents	Application Starting Page
3.2.3.4 Blinding/Unblinding	47
3.2.3.5 Discontinuation/Withdrawal from Study	47
3.3 Efficacy/Pharmacokinetic/Immunogenicity, etc. Measurements	48
3.3.1 Pharmacodynamic Measurements	48
3.3.2 Pharmacokinetic Measurements-Archive Plasma for MK-0859	48
3.3.3 Blood for Pharmacogenomic Analysis	48
3.3.4 Medication Compliance	49
3.4 Safety Measurements	49
3.4.1 Clinical and Laboratory Measurements for Safety	49
3.4.2 Recording Adverse Experiences	49
3.4.3 Definition of an Overdose for This Protocol	50
3.4.3.1 Reporting of Overdose to SPONSOR	50
3.4.4 Reporting of Pregnancy to SPONSOR	50
3.4.5 Immediate Reporting of Adverse Experiences to the SPONSOR	50
3.4.5.1 Serious Adverse Experiences	50
3.4.6 Evaluating Adverse Experiences	51
3.4.7 SPONSOR Responsibility for Reporting Adverse Experiences	54
3.5 Data Analysis	54
3.5.1 Hypotheses	54
3.5.2 Variables and Time Points	55
3.5.3 Approaches to Analyses	55
3.5.4 Statistical Methods	55
3.5.5 Assumptions	57
3.5.6 Multiplicity	57
3.5.7 Power Calculations	58

TABLE OF CONTENTS (CONT.)

Contents	Application Starting Page
3.6 Labeling, Packaging, Storage, Dispensing, and Return of Clinical Supplies	59
3.6.1 Subject and Replacements Information	59
3.6.2 Product Descriptions	59
3.6.3 Primary Packaging and Labeling Information	60
3.6.4 Secondary Packaging and Labeling Information (kit)	61
3.6.5 Clinical Supplies Disclosure	61
3.6.6 Storage and Handling Requirements	62
3.6.7 Standard Policies / Return of Clinical Supplies	62
3.6.8 Comparator Statement	62
3.7 Data Management	63
3.8 Biological Specimens	63
4. ADMINISTRATIVE AND REGULATORY DETAILS	64
4.1 Confidentiality	64
4.1.1 Confidentiality of Data	64
4.1.2 Confidentiality of Subject/Patient Records	64
4.1.3 Confidentiality of Investigator Information	64
4.2 Compliance with Law, Audit, and Debarment	65
4.3 Compliance with Financial Disclosure Requirements	66
4.4 Quality Control and Quality Assurance	67
4.5 Compliance with Information Program on Clinical Trials for Serious or Life Threatening Conditions	67
4.6 Publications	67
5. LIST OF REFERENCES	68
6. APPENDICES	70
6.1 Calculating Body Mass Index (BMI)	70
6.2 Laboratory Safety Tests	71

TABLE OF CONTENTS (CONT.)

Contents	Application Starting Page
6.3 Algorithm for Assessing Out-of Range Laboratory Values	72
6.4 Blood Drawn During Study	73
6.5 Archive Plasma for PCSK9 Levels—Sample Collection, Handling, Labeling, Storage, and Shipment	74
6.6 Archive Serum for CETP Activity—Sample Collection, Handling, Labeling, Storage, and Shipment	76
6.7 Archive Plasma for MK-0859—Sample Collection, Handling, Labeling, Storage, and Shipment	78
6.8 CCI [redacted]	81
6.9 Expected Effects for Intra- and Inter-Panel Comparisons	83
6.10 Procedure to Measure Fasting LDL-C and HDL-C (If TG are >400 mg/dL)	84
7. ATTACHMENTS	85
8. SIGNATURES	118
8.1 SPONSOR'S REPRESENTATIVE	118
8.2 INVESTIGATOR	118

1. SUMMARY

1.1 TITLE

A Multiple Dose Study to Investigate the Effect of MK-0859 on Lipoprotein Metabolism When Added to Ongoing Statin Therapy in Dyslipidemic Patients

1.2 INDICATION

Treatment of Dyslipidemia

1.3 SUMMARY OF RATIONALE

MK-0859 is a promising cholesteryl ester transfer protein (CETP) inhibitor being developed for the treatment of dyslipidemias, including primary hypercholesterolemia and mixed hyperlipidemia, which increase susceptibility to atherosclerotic cardiovascular disease. In clinical studies to date, MK-0859 administration has resulted in favorable decreases in low-density lipoprotein cholesterol (LDL-C; up to ~60%) and increases in high-density lipoprotein cholesterol (HDL-C; up to ~120%). The primary purpose of this study is to elucidate the metabolic mechanisms by which CETP inhibition with MK-0859 mediates reductions in LDL-C on a background of statin treatment.

1.4 SUMMARY OF STUDY DESIGN

This is a multi-center, randomized, double-blind, double-dummy, placebo-controlled, 2-period, fixed-sequence study in dyslipidemic patients. Patients allowed to participate in the study will be those treated with a statin as well as statin-naïve patients. Patients currently taking a statin will undergo a 2- to 3-week washout period prior to randomization. Patients that are statin-naïve can be randomized immediately if they otherwise qualify for the study. Approximately 40 dyslipidemic patients will be randomized according to an allocation schedule in a double-blinded manner into either Panel A or Panel B (in a 3:1 ratio).

Panel A (N=30): In Period 1, patients will receive atorvastatin 20 mg (LIPITOR™) co-administered with placebo to MK-0859 (PBO to MK-0859) for a minimum of 4 weeks (maximum of 5 weeks). In Period 2, patients will receive MK-0859 100 mg co-administered with atorvastatin 20 mg once daily for 8 weeks (maximum of 9 weeks).

Panel B (N=10): In Period 1, patients will receive placebo to atorvastatin 20 mg (PBO to atorvastatin) co-administered with PBO to MK-0859 for a minimum of 4 weeks (maximum of 5 weeks). In Period 2, patients will receive MK-0859 100 mg co-administered with PBO to atorvastatin once daily for 8 weeks (maximum of 9 weeks).

There will not be a washout between periods in either Panel.

All patients will undergo a lipoprotein kinetic study at the end of Period 1 and at the end of Period 2. [redacted] CCI

[redacted] Blood samples will be collected at 0 (pre-bolus), 20 and 40 minutes, and 1, 2, 4, 6, 8, 10, 12, 14, 15, 15.5, 16, 18, 21, 24, and 48 hours post-infusion to determine the kinetics of apolipoprotein (apo) B100 in LDL, very low-density lipoprotein (VLDL) and intermediate-density lipoprotein (IDL); apoA-I and apoA-II in high-density lipoprotein (HDL); and apo(a) in Lp(a). Archive samples will also be obtained for possible determination of VLDL Triglyceride (TG) production rate, and PCSK9 and CETP kinetics.

Blood samples will also be collected to determine the concentrations of lipoprotein lipase (LPL), hepatic lipase (HL) and apolipoproteins, as well as activities of LPL, HL and lecithin-cholesterol acyltransferase (LCAT). Archive samples will also be obtained for possible determination of PCSK9, lathosterol, and prebeta HDL concentrations and lipoprotein size by NMR. Samples for determination of CETP activity and mass will also be archived. Plasma samples for MK-0859 concentrations will be obtained and archived on the day prior to initiation of the kinetic assay in period 1 and during period 2 at the time points specified in the Study Flow Chart.

Safety will be monitored throughout the study by repeated clinical and laboratory evaluation.

A schematic representation of the study design is presented in Figure 1-1.

Figure 1-1

Schematic Representation of MK-0859 Protocol #026 Study Design

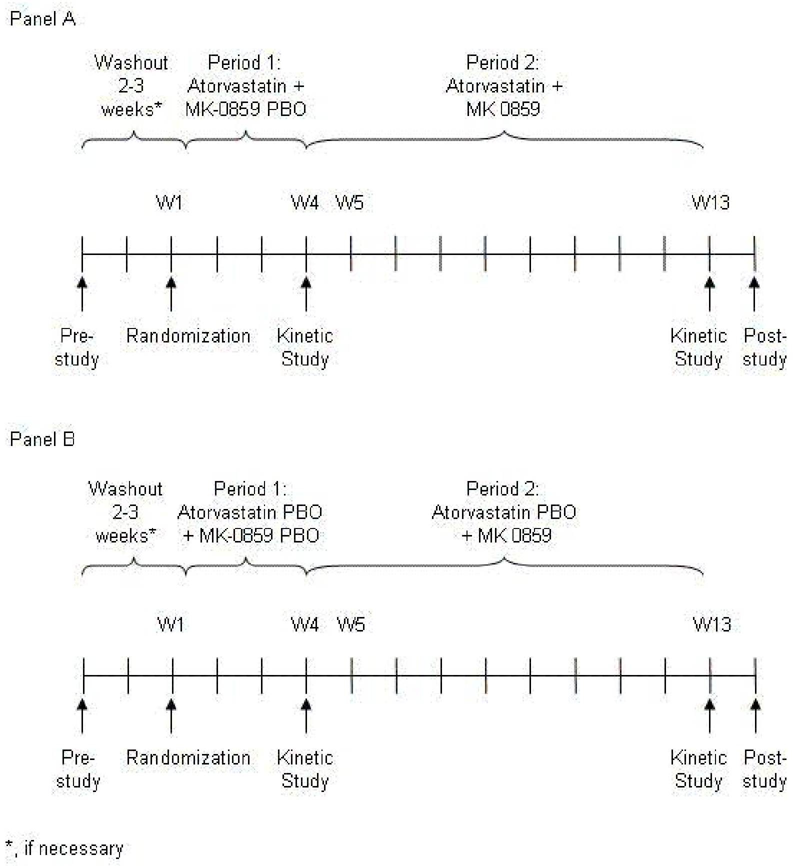

1.5 SAMPLE

Approximately, forty (40) dyslipidemic male or female patients between the ages of 18 and 75 years of age (inclusive) who fulfill the entry criteria outlined in Section 2.2 and 2.3 will be enrolled in the study.

All patients who participate in the study will have a TG level of ≤ 400 mg/dL at the screening visit. All patients will have an LDL-C level of ≥ 100 mg/dL and ≤ 190 mg/dL (if they have no or 1 cardiac risk factor according to NCEP guidelines) or ≤ 160 mg/dL (if they have 2 or more cardiac risk factors according to NCEP guidelines) at the screening visit for statin-naïve patients, or at Visit 2 for patients that are undergoing the 2-3 week washout period for statins.

1.6 DOSAGE/DOSAGE FORM, ROUTE, AND DOSE REGIMEN

In Period 1, Panel A will receive atorvastatin 20 mg (1 x 20 mg tablet) co-administered with PBO to MK-0859 (1 x PBO to MK-0859 tablet) and Panel B will receive PBO to

atorvastatin (1 x PBO to atorvastatin tablet) and PBO to MK-0859 (1 x PBO to MK-0859 tablet) daily for 4 to 5 weeks followed by a lipoprotein kinetic assay. Patients will continue to receive the study drug regimen during the lipoprotein kinetic assay (total dosing days in Period 1 will be 30 to 37 days).

In Period 2, Panel A will receive 100 mg MK-0859 (1 x 100 mg tablet) co-administered with atorvastatin 20 mg (1 x 20 mg tablet) for 8 to 9 weeks, followed by a lipoprotein kinetic assay. Panel B will receive 100 mg MK-0859 (1 x 100 mg tablet) co-administered with PBO to atorvastatin (1 x PBO to atorvastatin tablet) for 8 to 9 weeks, followed by a kinetics assay. Patients will continue to receive the study drug regimen during the kinetic assay (total dosing days in Period 2 will be 57 to 64 days). A sample treatment plan is provided in Table 1-1.

In both periods oral doses will be taken at home with the main meal of the day (dose in the evening with dinner), with the exception of the doses given during the kinetics assays, which will be given in the clinical research center (CRC). Compliance will be monitored by pill counts and random phone calls by the research staff. In Period 2, the exact time of dosing on the day prior to Visit 8 (Day 55, Period 2) and for the doses given in the CRC will be recorded on the case report forms (CRF).

Table 1-1

Treatment Plan

Panel	Patients (N)	Period 1	Period 2
A	30	Atorvastatin 20 mg +<br>PBO to MK-0859<br>(30 to 37 days)	MK-0859 100 mg + atorvastatin 20 mg<br>(57 to 64 days)
B	10	PBO to atorvastatin 20 mg + PBO to MK-0859<br>(30 to 37 days)	MK 0859 100 mg + PBO to atorvastatin 20 mg<br>(57 to 64 days)

1.7 STUDY FLOW CHART

	Pre-study	Washout 2-3 wks###	Period 1†††					Period 2¶¶¶¶					Poststudy†
			Random-ization										
Approximate Day:		Day -2	D1	D21##	D28‡	D29-30‡	D31	D1††††	D28‖	D56‖	D57-58‖	59‖	
Clinic Visit I.D.:	Visit 1	Visit 2###	Visit 3	Visit 4##	Visit 5‡		Visit 6	Visit 6	Visit 7‖	Visit 8‖		Visit 9‖	Visit 10
Informed consent (main study)	X												
Informed consent (genetic analysis)	X												
Medical history	X												
Blood for genetic analysis for archive					X								
Serum FSH test (if applicable)§	X												
Serum/Urine β-hCG‖‖‖	X			X	X				X	X			X
Physical examination	X				X				X	X			X
Electrocardiogram (12-lead)- semi-recumbent	X				X				X	X			X
Weight	X				X					X			X
Height	X												
Vital signs (HR, BP, RR, temperature)-semi-recumbent	X				X				X	X			X
Laboratory safety tests	X			X					X	X			X
Lipid panel#	X	X###		X	X				X	X			
Plasma lipoproteins and archived samples for lipoprotein size by NMR¶					X					X			
LCAT activity assay					X					X			
PCSK9, Lathosterol, and prebeta HDL concentration for archive					X					X			
Hepatic lipase and lipoprotein lipase analyses							X‡‡‡					X‡‡‡	
MK-0859 for archive					X§§§					X§§§---------------------		X	X§§§

0859, Protocol 026-03 Issue Date: 29-Jul-2010

	Pre-study	Washout 2-3 wks[###]	Period 1[†††]					Period 2[¶¶¶]					Poststudy[†]
			Random-ization										
Approximate Day:		Day -2	D1	D21[##]	D28[‡]	D29-30[‡]	D31	D1[††††]	D28[‖]	D56[‖]	D57-58[‖]	59[‖]	
Clinic Visit I.D.:	Visit 1	Visit 2[###]	Visit 3	Visit[##] 4	Visit[‡] 5		Visit 6	Visit 6	Visit 7[‖]	Visit 8[‖]		Visit 9[‖]	Visit 10
CETP (mass and activity) for archive					X					X			
Atorvastatin/PBO to MK-0859 or PBO to atorvastatin/PBO to MK-0859 administration[††]			X--	----	----	-----X							
Atorvastatin/MK-0859 or MK-0859/PBO to atorvastatin administration[††]								X----------------------	----	----	-----X		
CCI [redacted][§§]						X					X		
Blood samples for lipoprotein kinetics; archive samples for PCSK9 and CETP kinetics[¶¶]						X--------	-X				X--------	-X	
In-house stay[‖‖]					X	X				X	X		
Meals [‡‡]					X	X				X	X		
Study Drug Compliance					X				X	X			
Dispense study medication			X					X					
24-hour diet recall	X												
Dispense 3-day food record				X					X				
Evaluation of adverse experiences	X----------	----------	----------	----------	----------	----------	----------	----------	----------	----------	----------	-----X	X

† The poststudy visit should occur approximately 14 days after the last dose of study drug in Period 2.
‡ Visit 5 in Period 1 can occur between Days 28 and 35 (inclusive) of Period 1; subsequent procedures and days will be adjusted accordingly.
§ Postmenopausal females must be confirmed to be in the post menopausal range.
‖ Visit 7 in Period 2 should occur approximately 28 days after the start of Period 2. Visit 8 in Period 2 can occur between Days 56 and 63 (inclusive) of Period 2; subsequent procedures and days will be adjusted according to the actual day of the visit.
¶ Tests to be done include: apoA-I, apoA-II, apoB100, apoC-II, apoC-III, apoE and Lp(a) concentrations; samples will be archived for possible determination of lipoprotein size by NMR.
# Tests to be done include: TC, HDL-C, TG and LDL-C [LDL-C will be a calculated value (TC-HDL-C minus 20% TG)], per the automated standard in-house methods in all visits. If TG are >400 mg/dl, procedures for the measurement of fasting LDL-C and HDL-C as described in Appendix 6.10 will be employed.

	Pre-study	Washout 2-3 wks[###]	Period 1[†††]					Period 2[¶¶¶]					Poststudy[†]
			Random-ization										
Approximate Day:		Day -2	D1	D21[##]	D28[‡]	D29-30[‡]	D31	D1[††††]	D28[‖]	D56[‖]	D57-58[‖]	59[‖]	
Clinic Visit I.D.:	Visit 1	Visit 2[###]	Visit 3	Visit[##] 4	Visit[‡] 5		Visit 6	Visit 6	Visit 7[‖]	Visit 8[‖]		Visit 9[‖]	Visit 10

[††] Patients will dose at home with the main meal of the day (dose in the evening with dinner). The clinical staff will call the patients once or twice during each period to review the concomitant therapies/medical history/adverse experiences as well as to check the dosing compliance and remind them of dosing instructions. Study drug administration during Visit 5 (Period 1) and Visit 8 (Period 2) will be witnessed in the clinic. The exact time of dosing for doses given in the CRC and the day prior to Visit 8 (Day 55, Period 2) should be recorded on the CRF. Doses during Visit 8 are to be given at 5 PM on Day 56 and Day 57. The last dose of Period 2 is administered on Day 57.

[‡‡] A standard meal will be provided in the evening on Day 28 (Period 1) and Day 56 (Period 2) at approximately 5 PM. Starting on Day 29 (Period 1) and Day 57 (Period 2) Patients will be fed an isocaloric meal every 2 hours for 30 hours during the kinetic assay.

[§§] [redacted]

[‖‖] Patients will be admitted to the clinic the evening prior to the kinetic study on Visit 5 and on Visit 8, and will be discharged at the discretion of the investigator after the 24 hour blood sample of each kinetic study.

[¶¶] Blood samples for the lipoprotein turnover assay will be collected at 0 minutes (pre-bolus), 20 minutes, 40 minutes, and 1, 2, 4, 6, 8, 10, 12, 14, 15, 15.5, 16, 18, 21, 24, and 48 hours [redacted]. An aliquot from each sample will be archived for possible determination of VLDL Triglyceride (TG) production rate, and CETP and PCSK9 kinetics.

[##] Visit 4 should occur approximately 1 week prior to the start of the kinetic assay in Period 1. The time interval between Visit 4 and Visit 5 can be greater than 7 days, as long as dosing in Period 1 does not exceed 35 days prior to the conduct of the kinetics assay. Alternatively, the time interval between Visit 4 and Visit 5 can be less than 7 days.

[†††] Period 1 can be up to 38 days.

[‡‡‡] Samples will be obtained at the end of each kinetic assay (48 hours post start of kinetic assay) after heparin infusion. HL and LPL activities and concentrations will be determined.

[§§§] Samples for MK-0859 archive to be obtained at Visit 5 (Day 28 of Period 1) and during Visit 8: Day 56, predose, and 16 and 20 hours postdose; and on Day 57 predose, 2, 4, 8, 12, 16, 40, and ~240 hours postdose. The ~240 hour time point will be taken during the poststudy visit. The Visit 5 (Day 28 of Period 1) plasma sample for MK-0859 archive may be collected at any time on Day 28. The Visit 8 Day 56 and Day 57 predose plasma samples for MK-0859 archive are to be collected anytime within one hour of the Day 56 and Day 57 doses, respectively. Furthermore, it is not necessary to obtain the samples at the same time on Day 56 and Day 57 for a particular study participant.

[‖‖‖] A serum β-hCG will be determined at the prestudy and poststudy visits; a urine β-hCG can be determined at all other time points.

[¶¶¶] Period 2 can be up to 66 days.

[###] Washout Period applies only to patients on statin therapy at the time of screening; the lipid panel at Visit 2 will be determined only for patients currently on statin therapy.

[††††] Day 31 procedures in Period 1 are conducted on the same day as Period 2 Day 1.

2. CORE PROTOCOL

2.1 OBJECTIVES AND HYPOTHESES

2.1.1 Primary

1. **Objective:** To determine the effect of treatment with MK-0859 100 mg added to background statin therapy on the turnover [production rate (PR) and/or fractional catabolic rate (FCR)] of apolipoprotein (apo) B100 in low-density lipoprotein (LDL) in dyslipidemic patients.

   Hypothesis: The addition of MK-0859 to background atorvastatin therapy is associated with a decrease in PR of LDL apoB100. A true difference in LDL apoB100 PR obtained following Panel A, Period 2 compared to the LDL apoB100 PR obtained following Panel A, Period 1 (MK-0859 + atorvastatin versus atorvastatin) is expected.

   Hypothesis: The addition of MK-0859 to background atorvastatin therapy is associated with an increase in FCR of LDL apoB100. A true difference in LDL apoB100 FCR obtained following Panel A, Period 2 compared to the LDL apoB100 PR obtained following Panel A, Period 1 (MK-0859 + atorvastatin versus atorvastatin) is expected.

2.1.2 Secondary

1. **Objective:** To determine the effect of treatment with MK-0859 100 mg (compared with PBO) on the FCR of apolipoprotein (apo) B100 in low-density lipoprotein (LDL) in dyslipidemic patients.

   Hypothesis: The administration of MK-0859 is associated with an increase in FCR of LDL apoB100. A true difference in LDL apoB100 FCR obtained following Panel B, Period 2 compared to the LDL apoB100 FCR obtained following Panel B, Period 1 (MK-0859 versus PBO) is expected.

2.1.3 Tertiary

1. **Objective:** To determine the effect of treatment with MK-0859 100 mg per day on the FCR of apoA-I in high-density lipoprotein (HDL) in dyslipidemic patients.

   Hypothesis: The administration of MK-0859 is associated with a decrease in FCR of HDL apoA-I. A true difference in HDL apoA-I FCR obtained following Panel B, Period 2 compared to the HDL apoA-I FCR obtained following Panel B, Period 1 (MK-0859 versus PBO) is expected.

2. **Objective:** To determine the effect of treatment with MK-0859 100 mg added to background statin therapy on the FCR of HDL apoA-I in dyslipidemic patients.

Hypothesis: The addition of MK-0859 to background atorvastatin therapy is associated with a decrease in FCR of HDL apoA-I. A true difference in HDL apoA-I FCR obtained following Panel A, Period 2 compared to the HDL apoA-I FCR obtained following Panel A, Period 1 (MK-0859 + atorvastatin versus atorvastatin) is expected.

3. **Objective:** To determine the effect of treatment with atorvastatin 20 mg (compared with PBO) on the FCR of LDL apoB100 in dyslipidemic patients.

   Hypothesis: The administration of atorvastatin is associated with an increase in FCR of LDL apoB100. A true difference in LDL apoB100 FCR obtained following Panel A, Period 1 (atorvastatin 20 mg) compared to the LDL apoB100 FCR obtained following Panel B, Period 1 (PBO) is expected.

2.1.4 Exploratory

1. **Objective:** To determine the effect of MK-0859 100 mg plus atorvastatin (versus atorvastatin alone) on the pool size (PS) of LDL apoB100 in dyslipidemic patients.

2. **Objective:** To determine the effect of MK-0859 100 mg (versus PBO) on the PR and PS of LDL apoB100 in dyslipidemic patients.

3. **Objective:** To determine the with-in Panel A effects of MK-0859 100 mg plus atorvastatin (versus atorvastatin alone), and the with-in Panel B effects of MK-0859 100 mg (versus PBO) on the following in dyslipidemic patients:

   a. PR and PS of HDL apoA-I

   b. PS, PR and FCR of apoB100 in very low-density lipoprotein (VLDL) and intermediate-density lipoprotein (IDL)

   c. PS, PR and FCR of apo(a) in Lp(a)

   d. PS, PR and FCR of apoA-II in HDL

   e. concentration and/or activities of proteins involved in the modulation of VLDL, LDL, and HDL lipid composition, including LCAT, LPL and HL

   f. concentration of apoA-I, apoA-II, apoB100, apoC-II, apoC-III, apoE and Lp(a)

   g. percent conversion of VLDL apoB100 to IDL apoB100, VLDL apoB100 to LDL apoB100, and IDL apoB100 to LDL apoB100

4. **Objective:** To determine the cross-panel effects (atorvastatin versus PBO; atorvastatin versus MK-0859 100 mg; PBO versus MK-0859 100 mg plus atorvastatin; MK-0859 100 mg versus MK-0859 100 mg plus atorvastatin) on the following in dyslipidemic patients:

   a. PR and PS of LDL apoB100

b. PS, PR and FCR of HDL apoA-I

c. PS, PR and FCR of apoB100 in very low-density lipoprotein (VLDL) and intermediate-density lipoprotein (IDL)

d. PS, PR and FCR of apo(a) in Lp(a)

e. PS, PR and FCR of apoA-II in HDL

f. concentration and/or activities of proteins involved in the modulation of VLDL, LDL, and HDL lipid composition, including LCAT, LPL and HL

g. concentration of apoA-I, apoA-II, apoB100, apoC-II, apoC-III, apoE and Lp(a)

h. percent conversion of VLDL apoB100 to IDL apoB100, VLDL apoB100 to LDL apoB100, and IDL apoB100 to LDL apoB100

2.2 PATIENT INCLUSION CRITERIA

Demographics

1. Patient is a male or female between 18 to 75 years of age.

   Note: Women who are postmenopausal or status post hysterectomy, oophorectomy (patient recall) or tubal ligation (patient recall) are eligible for participation in the study.

   Postmenopausal is defined as no menses for > than 1 year **and** an FSH value in the postmenopausal range upon prestudy (screening) evaluation.

   Note: Female subjects of reproductive potential must demonstrate a serum β-hCG level consistent with the nongravid state at the prestudy (screening) visit and agree to use (and/or have their partner use) two acceptable methods of birth control beginning at least 2 weeks prior to administration of the first dose of study drug, throughout the study and until at least 2 weeks after administration of the last dose of study drug in the last treatment period. Acceptable methods of birth control are abstinence, or 2 of the following: intrauterine device (IUD-with or without local hormone release), diaphragm, spermicides, cervical cap, contraceptive sponge, and condoms.

2. Patient has a stable weight (± 3 kg) for >6 weeks prior to screening (based on patient recall).

3. The patient has a Body Mass Index (BMI) of $18.5 \leq \text{BMI} \leq 40$ kg/m^2 at the prestudy (screening) visit. BMI is calculated by taking the patient's weight in kg and dividing by the patient's height in meters, squared (Appendix 6.1).

Medical history, physical examinations, laboratory safety tests and ECG measurement

4. Patient is judged to be in good health based on medical history, physical examination, vital sign measurements, and laboratory safety tests (see Appendices 6.2 and 6.3) performed at the pre-study (screening) visit and/or prior to administration of the initial dose of study drug.

5. Patient has a TG level of ≤ 400 mg/dL at the screening visit. All patients will have an LDL-C level of ≥ 100 mg/dL and ≤ 190 mg/dL (if they have no or 1 cardiac risk factor according to NCEP guidelines) or ≤ 160 mg/dL (if they have 2 or more cardiac risk factors according to NCEP guidelines) at the screening visit for statin-naïve patients, or at Visit 2 for patients that are undergoing the 2-3 week washout period for statins.

   Note: TC, HDL-C, TG and LDL-C [LDL-C will be a calculated value (TC-HDL-C minus 20% TG)] will be determined per the automated standard in-house methods in all visits. If TG are >400 mg/dl, procedures for the measurement of fasting LDL-C and HDL-C as described in Appendix 6.10 will be employed.

6. Patient has no clinically significant abnormality on electrocardiogram (ECG) performed at the pre-study (screening) visit and/or prior to administration of the initial dose of study drug.

Diet/Activity/Other

7. Patient has been a nonsmoker and/or has not used nicotine or nicotine-containing products for at least approximately 6 months and does not plan to begin smoking during the conduct of the study; patients who have discontinued smoking or the use of nicotine/nicotine containing products for at least approximately 3 months may be enrolled in the study at the discretion of the investigator. Non-daily or social smokers who have consumed no greater than approximately 5 cigarettes or equivalent over the 3 month period prior to screening also may be enrolled at the discretion of the investigator.

8. Patient understands the study procedures and agrees to participate in the study by giving written informed consent.

9. Patient is willing to comply with the study restrictions (see Section 3.2. for a complete summary of study restrictions).

10. Patient is able to follow the American Heart Association NCEP diet.

2.3 PATIENT EXCLUSION CRITERIA

Medical history, physical examinations, laboratory safety tests and ECG measurements

1. Patient is under the legal age of consent

2. Patient is mentally or legally incapacitated, has significant emotional problems at the time of pre-study (screening) visit or expected during the conduct of the study or has a history of a clinically significant psychiatric disorder over the last 5 years. Patients who have had situational depression may be enrolled in the study at the discretion of the investigator.

3. Patient has an estimated creatinine clearance of ≤ 60 mL/min based on the Cockcroft-Gault equation; the Cockcroft-Gault equation is:

   $$Cl_{Cr} = \frac{(140\text{-age[yr]})(\text{body wt [kg]})}{(72)(\text{serum creat [mg/dL]})}$$

   An actual creatinine clearance, as determined by a 24-hour urine collection, may be used in place of, or in conjunction with, the Cockcroft-Gault equation; patients who have an actual or estimated creatinine clearance up to 10% below 60 mL/min may be enrolled at the discretion of the investigator.

4. Patient has a history of stroke, chronic seizures, or major neurological disorder.

5. Patient has a history of clinically significant endocrine, thyroid, gastrointestinal, cardiovascular, hematological, hepatic, immunological, renal, respiratory, or genitourinary abnormalities or diseases.

   Note: Patients with controlled hypertension (≤ 150/100 mmHg), if they are on a stable medical regimen (for at least 4 weeks) may be allowed at the discretion of the investigator.

   Note: For patients on thyroid hormone replacement treatment at the time of screening, there is no lower TSH threshold for entry. The patient must have been on a stable dose of thyroid hormone therapy for ≥ 6 weeks prior to the screening. If TSH levels are undetectable and the patient requires a change in thyroid hormone therapy or this represents a new diagnosis, then the patient will be excluded.

   Note: Hypothyroidism is defined as having a TSH >20% above the local laboratory's upper limit of the normal reference range.

   One redraw will be allowed if the original TSH value is equal to or less than 40% above the normal reference range at the local laboratory. The patient must meet the criterion upon redraw.

6. Patient has a history of neoplastic disease.

   Exceptions: (1) Patients with adequately treated non-melanomatous skin carcinoma; (2) Patients with other malignancies which have been successfully treated ≥10 years prior to the pre-study (screening) visit where, in the judgment of both the investigator and treating physician, appropriate follow-up has revealed no evidence of recurrence from the time of treatment through the time of the pre-study (screening) visit; or, (3) Patients, who, in the opinion of the study investigator, are highly unlikely to sustain a recurrence for the duration of the study.

7. Patient has a history of any illness that, in the opinion of the study investigator, might confound the results of the study or poses an additional risk to the patient by their participation in the study.

8. Patient is a nursing mother.

Medications

9. Patient is currently taking lipid-lowering medications (except for statins) or medications known to alter lipoprotein metabolism (e.g., glucocorticoids, oral contraceptives, anabolic agents, omega-3, etc.).

   Note: Hormone replacement therapy may be allowed at the discretion of the investigator, if the patient is on a stable dose for at least 6 weeks.

   Note: Low dose thiazide diuretics (12.5 mg hydrochlorothiazide or equivalent) may be allowed at the discretion of the investigator.

   Note: Stable doses (for at least 4 weeks) of beta-blockers may be allowed at the discretion of the investigator.

   Note: Stable doses of thyroid replacement treatment are allowed; for more details, please refer to Section 2.3, item #5 above.

10. Patient is currently taking fiber-based laxatives, phytosterol margarines, and/or over the counter (OTC) therapies that are known to affect serum lipids or anticipates use during the study.

    Note: Psyllium may be allowed at the discretion of the investigator.

11. Patient has taken lipid-lowering agents (except for statins) including fish oils, fibrates, Cholestin™ (Pharmanex, Inc.; also known as red yeast rice, or *Monascus purpureus* extract), or niacin (vitamin B_3) (>200 mg/day) within 6 weeks prior to screening or anticipates use during the study.

    Note: If a patient has taken lipid-lowering agents (except for statins) including fish oils, fibrates, Cholestin™ (Pharmanex, Inc.; also known as red yeast rice, or

Monascus purpureus extract), or niacin (vitamin B_3) (>200 mg/day) within 6 weeks prior to screening, the patient may be rescreened after 6 weeks has elapsed from last use.

12. Patient has used any investigational drugs or participated in another investigational study within 30 days of the screening visit.

    Note: If a patient has participated in another investigational study involving administration of isotopic material, or lipid-modifying compound within the last 6 months, then the SPONSOR medical monitor should be consulted for eligibility prior to enrollment.

13. Patient is unable to refrain from or anticipates the use of any new medication, including prescription and non-prescription drugs or herbal remedies (such as St. John's Wort [hypericum perforatum]), and/or any other drugs (herbal or organic or homeopathic) or dietary or nutritional remedies that are known to influence plasma lipid levels beginning approximately 2 weeks (or 5 half-lives) prior to administration of the initial dose of study drug, throughout the study (including washout intervals between treatment periods), and until the poststudy visit.

Diet/Activity/Other

14. Patient consumes excessive amounts of alcohol, defined as greater than 2 glasses of alcoholic beverages (1 glass is approximately equivalent to: beer [12 ounces], wine [5 ounces], or distilled spirits [1.5 ounce]) per day.

15. Patient consumes excessive amounts, defined as greater than 6 servings (1 serving is approximately equivalent to 120 mg of caffeine) of coffee, tea, cola, or other caffeinated beverages per day.

    Note: Patients who consume greater than 6 servings of caffeine may be enrolled at the discretion of the investigator.

16. Patient has had major surgery, donated or lost 1 unit of blood (approximately 500 mL) or participated in another investigational study within 4 weeks prior to the prestudy (screening).

17. Patient has a history of significant multiple and/or severe allergies (including latex), or has had an anaphylactic reaction or significant intolerability to prescription or non-prescription drugs or food.

    Note: Patients with a history of minor allergies may be enrolled at the discretion of the investigator.

18. Patient is currently a regular user (including "recreational use") of any illicit drugs or has a history of drug (including alcohol) abuse within approximately 2 years.

19. Patient is an employee of the clinical research unit.

    Note: Other hospital or university employees may participate in the study, provided they qualify based on other inclusion and exclusion criteria.

20. There is any concern by the investigator regarding the safe participation of the patient in the study or for any other reason; the investigator considers the patient inappropriate for participation in the study.

2.4 STUDY DESIGN AND DURATION

2.4.1 Summary of Study Design

This is a multi-center, randomized, double-blind, double-dummy, placebo-controlled, 2-period, fixed-sequence study in dyslipidemic patients. Patients allowed to participate in the study will be those treated with a statin as well as statin-naïve patients. Patients currently taking a statin will undergo a 2- to 3-week washout period prior to randomization. Patients that are statin-naïve can be randomized immediately if they otherwise qualify for the study. Approximately 40 dyslipidemic patients will be randomized according to an allocation schedule in a double-blinded manner into either Panel A or Panel B (in a 3:1 ratio).

Panel A (N=30): In Period 1, patients will receive atorvastatin 20 mg co-administered with placebo to MK-0859 (PBO to MK-0859) for a minimum of 4 weeks (maximum of 5 weeks). In Period 2, patients will receive MK-0859 100 mg co-administered with atorvastatin 20 mg once daily for 8 weeks (maximum of 9 weeks).

Panel B (N=10): In Period 1, patients will receive placebo to atorvastatin 20 mg (PBO to atorvastatin) co-administered with PBO to MK-0859 for a minimum of 4 weeks (maximum of 5 weeks). In Period 2, patients will receive MK-0859 100 mg co-administered with PBO to atorvastatin once daily for 8 weeks (maximum of 9 weeks).

There will not be a washout between periods in either Panel.

All patients will undergo a lipoprotein kinetic study at the end of Period 1 and at the end of Period 2. Patients will start receiving a small meal (iso-caloric meal consisting of ~18% fat) every 2 hours (16 meals over 30 hours)] approximately 8 hours prior to the start of each kinetic assay. CCI [redacted] Blood samples will be collected at 0 (pre-bolus), 20 and 40 minutes, and 1, 2, 4, 6, 8, 10, 12, 14, 15, 15.5, 16, 18, 21, 24, and 48 hours post-infusion to determine the kinetics of apoB100 in LDL, VLDL and IDL, apoA-I and apoA-II in HDL, and apo(a) in Lp(a). Archive samples will also be obtained for possible determination of VLDL TG production rate, and PCSK9 and CETP kinetics.

Blood samples will also be collected to determine the concentrations of lipoprotein lipase (LPL), hepatic lipase (HL) and apolipoproteins, as well as activities of LPL, HL and lecithin-cholesterol acyltransferase (LCAT). Archive samples will also be obtained for possible determination of PCSK9, lathosterol, and prebeta HDL concentrations and lipoprotein size by NMR. Samples for determination of CETP activity and mass will also be archived. Plasma samples for MK-0859 concentrations will be obtained and archived during period 1 and period 2 at the time points specified in the Study Flow Chart.

Safety will be monitored throughout the study by repeated clinical and laboratory evaluation.

The duration of the study for an individual patient will be approximately 22 weeks (including the prestudy and poststudy visits).

2.4.2 Treatment Plan

In Period 1, Panel A will receive atorvastatin 20 mg (1 x 20 mg tablet) co-administered with PBO to MK-0859 (1 x PBO to MK-0859 tablet) and Panel B will receive PBO to atorvastatin (1 x PBO to atorvastatin tablet) and PBO to MK-0859 (1 x PBO to MK-0859 tablet) daily for 4 to 5 weeks prior to the first lipoprotein kinetic assay. Patients will continue to receive study drug during the lipoprotein kinetic assay (total dosing days in Period 1 will be 30 to 37 days).

In Period 2, Panel A will receive 100 mg MK-0859 (1 x 100 mg tablet) co-administered with atorvastatin 20 mg (1 x 20 mg tablet) for 8 to 9 weeks, followed by a lipoprotein kinetic assay. Panel B will receive 100 mg MK-0859 (1 x 100 mg tablet) co-administered with PBO to atorvastatin (1 x PBO to atorvastatin tablet) for 8 to 9 weeks, followed by a kinetics assay. Patients will continue to receive study drug during the kinetics assay (total dosing days in Period 2 will be 57 to 64 days).

Study drugs will be administered orally with the main meal of the day (dose in the evening with dinner). Dosing will be done at home with the exception of doses administered during Visit 5 (Period 1) and Visit 8 (Period 2) which will be at the CRC. Compliance will be monitored by pill count and random phone calls by the research staff. In Period 2, the exact time of dosing on the day prior to Visit 8 (Day 55, Period 2) and for the doses given in the CRC will be recorded on the case report forms (CRF).

Patients will be randomized to Panel A or Panel B according to an allocation schedule. Patients with LDL-C levels at randomization of <160 mg/dL will receive the next lowest allocation number, and patients with LDL-C levels at randomization of ≥160 mg/dL will receive the next highest allocation number, to be sure that the patients are allocated to each panel at a 3:1 ratio according to LDL-C level.

The investigator and patient will be blinded to treatment. A sample allocation schedule is in Table 2-1.

Table 2-1

Sample Allocation Schedule

Panel	Patients	Period 1‡		Period 2‡	
A	N = 30	20 mg atorvastatin + PBO to MK-0859 (30† days)		100 mg MK-0859 + 20 mg atorvastatin x 57† days	
B	N = 10		PBO to atorvastatin + PBO to MK-0859 (30† days)		100 mg MK-0859 + PBO to atorvastatin (57† days)

† Dosing can continue up to an additional 7 days.
‡ All patients will undergo a lipoprotein kinetic study at the end of Period 1 and at the end of Period 2. CCI [redacted]

2.5 LIST OF EFFICACY/PHARMACOKINETIC/IMMUNOGENICITY, ETC., MEASUREMENTS

2.5.1 Pharmacodynamic Measurements

Blood samples will be collected at 0 (pre-bolus), 20 and 40 minutes, and 1, 2, 4, 6, 8, 10, 12, 14, 15, 15.5, 16, 18, 21, 24, and 48 hours post-infusion for the isolation of plasma VLDL, IDL, LDL, and HDL using ultracentrifugation. ApoB100, apoA-I, apoA-II and apo(a) will be isolated from lipoprotein fractions using SDS PAGE. CETP and PCSK9 may be isolated from archived plasma using immunoaffinity chromatography (samples will be archived for possible determination of CETP and PCSK9 kinetics). CCI [redacted] Production and fractional clearance rates of apoB100 in LDL, VLDL and IDL, apoA-I and apoA-II in HDL and apo(a) in Lp(a) will be calculated. Comparisons between groups will be performed for percent conversion of VLDL apoB100 to IDL apoB100, VLDL apoB100 to LDL apoB100, and IDL apoB100 to LDL apoB100. Additional samples will be archived for possible determination of VLDL TG production rate and PCSK9 and CETP kinetics.

Plasma will be analyzed to determine the concentrations of lipoprotein lipase (LPL), hepatic lipase (HL) and apolipoproteins, as well as activities of LPL, HL and lecithin-cholesterol acyltransferase (LCAT). Additional samples will be archived for possible determination of PCSK9, lathosterol and prebeta concentrations and lipoprotein size by NMR. Samples for determination of CETP activity and mass will also be archived.

2.5.2 Pharmacokinetic Measurements (for archive only)

Archive plasma samples for MK-0859 will be obtained during Visit 5 (Day 28, Period 1) and at several time points during period 2; on Day 56 predose, and 16 and 20 hours postdose; on Day 57 predose and over a 240-hour period following the last dose of MK-0859 on Day 57.

For specific time points please refer to the Study Flow Chart. See Appendix 6.5 for sample handling and processing procedures.

2.6 LIST OF SAFETY MEASUREMENTS

Safety will be monitored throughout the study by repeated clinical and laboratory evaluation including vital signs, physical exam, 12-lead ECG, laboratory safety parameters (hematology, chemistry, and urinalysis), plasma lipids (total cholesterol, HDL, LDL and triglycerides) and serum/urine β-hCG. Patients will be monitored for adverse experiences throughout the study.

2.7 DATA ANALYSIS SUMMARY

Method: The primary hypotheses will be addressed by analyzing both the production rate (PR) and the fractional catabolic rate (FCR) of LDL apoB100 with separate linear mixed effects models containing fixed effects for panel and treatment within panel, and a random effect for subject within panel. Summary statistics including point estimates, 95% confidence intervals (CIs) and between-treatment p-values (two-tailed) for the true mean differences (MK-0859 w/atorvastatin - atorvastatin) in LDL apoB100 PR and LDL apoB100 FCR will be calculated based on their respective models. The 4A multiple testing procedure of Li and Mehrotra (2008) [1] will be followed in order to account for the disparity in statistical power for the two primary endpoints. If the p-value for the true mean difference (MK-0859 w/atorvastatin versus atorvastatin) in LDL apoB100 PR is statistically significant at the α_1=0.04 level, the corresponding true mean difference for LDL apoB100 FCR will be tested at the full α_2=0.05 level. However, if the true mean difference for LDL apoB100 PR is not significant at α_1=0.04, the true mean difference for LDL apoB100 FCR will be tested at an adjusted α_2 level based on both the observed p-value for PR and the correlation among the two endpoints. The primary hypothesis will be supported if the test for either endpoint is significant at their respective α level. That is, administration of MK-0859 on a background of atorvastatin therapy is associated with a significant change in LDL apoB100 turnover.

The secondary hypothesis will be addressed by an analyzing the fractional catabolic rate of LDL apoB100 with a linear mixed effects model containing fixed effects for panel and treatment within panel, and a random effect for subject within panel. Summary statistics including point estimates, 95% CIs and between-treatment p-values (two-tailed) for the true mean differences (MK-0859 – placebo; atorvastatin - placebo) in LDL apoB100 FCR will be calculated based on this model.

The tertiary hypotheses and exploratory objectives will each be addressed by analyzing each tertiary/exploratory endpoint with a similar linear mixed effects model as described above. Point estimates, 95% CIs and between-treatment p-values (two-tailed) will be calculated for all of the treatment differences in these endpoints.

Power: <u>Primary Hypotheses</u>: Assuming a pooled, within-subject standard deviation of 1.94 mg·kg/d, N=30 subjects in Panel A, and a significance level of 0.04 (two-tailed), there is 96.0% probability to detect a -1.4 mg·kg/d decrease in LDL apoB100 PR for

MK-0859 administered on a background of atorvastatin therapy compared to atorvastatin alone. Assuming a pooled, within-subject standard deviation of 0.05 pools/d, N=30 subjects in Panel A, and a significance level of 0.05 (two-tailed), an increase of 0.026 pools/d in LDL apoB100 FCR can be detected with 80.0% probability for MK-0859 administered on a background of atorvastatin therapy compared to atorvastatin alone. Secondary hypothesis: Assuming a pooled, within-subject standard deviation of 0.05 pools/d for LDL apoB100 FCR, N=10 subjects in Panel B, and a significance level of 0.05 (two-tailed), there is a probability of 95.4% to detect a 0.065 pools/d increase in FCR of LDL apoB100 by MK-0859 compared to placebo.

3. PROTOCOL DETAILS

3.1 RATIONALE

CCI [redacted]

- [redacted]
- [redacted]
- [redacted]
- [redacted]
- [redacted]
- [redacted]
- [redacted]
- [redacted]

3.1.1 Rationale for This Study

MK-0859 is a promising CETP inhibitor being developed for the treatment of dyslipidemias, including primary hypercholesterolemia and mixed hyperlipidemia, which increase susceptibility to atherosclerotic cardiovascular disease. CETP inhibition with MK-0859 has been shown to significantly and favorably alter lipoprotein concentrations in the blood by as yet unknown mechanisms. The purpose of this study is to elucidate the mechanisms whereby MK-0859 affects lipoprotein concentrations in dyslipidemic patients on background statin therapy.

CCI [redacted].

Effects of CETP inhibition on plasma lipoproteins are expected, although the precise mechanisms are not completely clear. CETP plays an important role in mediating the bidirectional exchange of cholesteryl ester and triglyceride between HDL and the apoB-containing lipoproteins, as well as among the different classes of apoB-containing lipoproteins. This results in a net transfer of cholesteryl ester from HDL to triglyceride

rich lipoproteins (TRL), such as VLDL and IDL, and net transfer of triglyceride from TRL to HDL. Thus, CETP provides a link between the metabolism of apoB-containing lipoproteins and HDL. Expression of CETP in mice, which normally lack CETP, or manipulation of CETP activity in other animals that express CETP, changes the plasma concentration of apoB-containing lipoproteins as well as the HDL-C concentration. In humans, CETP inhibitors raise HDL-C and lower LDL-C levels.

Recently, Millar and co-workers [2] reported the effects of the CETP inhibitor, torcetrapib, on plasma lipoproteins in patients selected for low HDL-C (<40 mg • dL^{-1}). In these patients, torcetrapib treatment resulted in a dose-dependent increase in HDL-C and reductions in triglyceride and LDL-C levels. Kinetic studies were performed to define the mechanism(s) by which torcetrapib, alone or on a background with the HMG-CoA reductase inhibitor, atorvastatin, influenced lipoprotein and apolipoprotein metabolism. Data from these studies form the bases on which predictions of the effects of MK-0859 on lipoprotein kinetics were formulated.

Our primary objective in this study is to investigate whether CETP inhibition by MK-0859 on a statin background reduces LDL-C plasma levels by altering the turnover (production rate and/or fractional catabolic rate) of LDL apoB100. This objective is based on the observation in the Millar et al torcetrapib studies that, in the setting of background therapy with atorvastatin 20 mg, the addition of torcetrapib 120 mg daily resulted in a -10 ± 19% change in LDL apoB100 production rate, while the LDL apoB100 catabolic rate was unchanged. This trend suggests that in statin-treated dyslipidemic patients LDL apoB100 production rate is decreased with CETP inhibition, which in turn leads to further reductions in LDL-C plasma levels. On background statin therapy, the proposed 100 mg dose of MK-0859 for this study is roughly equipotent in terms of effects on apoB100 abundance to 120 mg of torcetrapib daily [2; 3]. As a result, we are predicting that MK-0859 will have an effect on the production rate of apoB100 in LDL that is similar to that which was observed with torcetrapib. Specifically, we are hypothesizing that the addition of MK-0859 to background statin therapy will result in a decrease in PR of LDL apoB100. We anticipate a decrease in LDL apoB100 PR of 10%, or an absolute reduction of 1.4 mg·kg/d (from a predicted baseline of ~13.5 mg·kg/d). The power to detect an expected absolute difference of 1.4 mg·kg/d in LDL apoB100 PR, assuming an intrasubject SD of 1.94 mg·kg/d (derived from experimental data from the laboratories of the co-investigators) is 96% with a cohort size of 30.

However, after Millar et al added CETP inhibition with torcetrapib to a statin background, the change in LDL apoB100 PR was not statistically significant [2]. Thus, due to a relatively small sample size, the evidence is not conclusive that reduced PR may account for the additional decrease in the LDL apoB100 pool size which was observed after the addition of torcetrapib to atorvastatin. Since decreased LDL apoB pool size may also result from an increase in the LDL apoB100 fractional catabolic rate, we have included a hypothesis that the addition of CETP inhibition with MK-0859 to a statin background is associated with increased LDL apoB100 FCR. With a cohort size of 30, there will be 80% power to detect a 6% change in LDL apoB100 FCR (or an absolute change of 0.026 pools/d from a predicted baseline of 0.44 pools/d). In order to fulfill the

primary objective of this trial, either or both of the primary hypotheses must be proven. A multiplicity adjustment will be employed for the primary endpoints to control the overall Type I error of the primary hypothesis 5%.

The secondary objective of this study focuses on the effect of MK-0859 monotherapy on LDL apoB100 kinetics. Data from the Millar et al study suggested that torcetrapib monotherapy (at a dose of 120 mg BID) increased the rate of LDL apoB100 catabolism by 26 ± 28%. As a result, the LDL apoB100 pool size decreased significantly by 19%. Thus, CETP inhibition appears to have a catabolic effect on LDL apoB100 in statin-naïve patients. As a result, we are hypothesizing that treatment with 100 mg of MK-0859 (in the absence of statin therapy) will increase the FCR of LDL apoB100. In statin-naïve patients, MK-0859 100 mg and torcetrapib 120 mg BID are roughly equipotent in their effects on apoB100 abundance. Thus, we are anticipating that treatment with 100 mg of MK-0859 will increase the FCR of LDL apoB100 by 26%, corresponding to an absolute increase of 0.065 pools/d (from a predicted baseline of ~0.25 pools/d). The power to detect an expected absolute difference of 0.065 pools/d in LDL apoB100 FCR, assuming an intrasubject SD of 0.05 pools/d (derived from experimental data from the laboratories of the co-investigators) is 95% with a cohort size of 10.

The effects of MK-0859 on HDL apoA-I metabolism in statin-naïve and statin-treated patients will be the focus of two of the tertiary objectives. Based on the torcetrapib experience [4], we hypothesize that CETP inhibition with MK-0859 in the absence of statin background therapy (tertiary hypothesis #1) or in the presence of statin co-therapy (tertiary hypothesis #2) is associated with a decrease in the FCR of HDL apoA-I. In statin-naïve patients, MK-0859 100 mg and torcetrapib 120 mg BID are roughly equipotent in their effects on apoA-I abundance. As a result, we anticipate that treatment with 100 mg of MK-0859 as monotherapy (as per tertiary hypothesis #1) will decrease the FCR of HDL apoA-I by 21%, corresponding to an absolute decrease of 0.048 pools/d (from a predicted baseline of 0.228 pools/d). The power to detect an expected absolute difference of 0.048 pools/d in HDL apoA-I FCR, assuming an intrasubject SD of 0.030 pools/d (derived from experimental data from the laboratories of the co-investigators) is 99.4% with a cohort size of 10. In statin-treated patients, MK-0859 100 mg has a > two-fold greater potency on apoA-I abundance compared to torcetrapib 120 mg QD. As a result, we anticipate that treatment with 100 mg of MK-0859 on a statin background (as per tertiary hypothesis #2) will decrease the FCR of HDL apoA-I by 14%, corresponding to an absolute decrease of 0.033 pools/d (from a predicted baseline of 0.237 pools/d). The power to detect an expected absolute difference of 0.033 pools/d in HDL apoA-I FCR, assuming an intrasubject SD of 0.030 pools/d (derived from experimental data from the laboratories of the co-investigators) is >99.9% with a cohort size of 10.

The third of the tertiary objectives is to investigate whether the previously described effect of atorvastatin on LDL apoB100 FCR [5] can be detected in the present study. We hypothesize that treatment with atorvastatin 20 mg will result in an increase in LDL apoB100 FCR. We anticipate that treatment with atorvastatin 20 mg will increase the FCR of LDL apoB100 by 81%, corresponding to an absolute increase of 0.20 pools/d

(from a predicted baseline of 0.25 pools/d). The power to detect an expected absolute difference of 0.20 pools/d in LDL apoB100 FCR, assuming an intersubject SD of 0.11 pools/d [5] is 99.9% with the proposed cohort sizes.

Additionally, because CETP transfers triglycerides from TRL to LDL in exchange for CE, inhibition of CETP with MK-0859 could have significant effects on the metabolism of apoB-containing lipoproteins that may not be directly related to exchange of lipids with HDL. Thus, the kinetics of apoB100 in VLDL and IDL will be investigated as exploratory objectives. Further assessment of the possible or anticipated effects on lipid kinetics in this study is provided in Appendix 6.9.

Interestingly, CETP inhibition (with either MK-0859 or torcetrapib) results in a dose-dependent increase in plasma CETP concentration over time. The mechanism responsible for the increased CETP plasma abundance is currently unknown. Possible mechanisms include: 1) reduced clearance from plasma due to association with HDL particles which are significantly increased in plasma following CETP inhibition; or 2) increased CETP production. We will archive serum in the current study to support potential future experiments to determine how treatment with MK-0859 influences CETP kinetics, mass and activity. Furthermore, we will archive serum to support possible investigations into how CETP inhibition affects the abundance of the regulatory molecule PCSK9.

In this study, randomized, double-blind, double-dummy, placebo-controlled, 2-period, fixed-sequence design will be used. Patients will be randomized into either Panel A or Panel B (in a 3:1 ratio). Panel A (N=30) will receive atorvastatin 20 mg co-administered with PBO to MK-0859 for 4 to 5 weeks (Period 1) followed by MK-0859 100 mg co-administered with atorvastatin 20 mg once daily for 8 to 9 weeks (Period 2). Panel B (N=10) will receive placebo to atorvastatin 20 mg (PBO to atorvastatin) co-administered with PBO to MK-0859 for 4 to 5 weeks (Period 1) followed by MK-0859 100 mg co-administered with PBO to atorvastatin once daily for 8 to 9 weeks (Period 2). CCI [redacted] Blood will be drawn at multiple time points during and after cessation of the infusion.

An 8-week duration for Period 2 in both Panels of this study was selected to insure that MK-0859 pharmacodynamics are at steady state, particularly with respect to changes in Lp(a). Incremental reductions in Lp(a) were observed in the previous Phase IIb MK-0859 study between 4 and 8 weeks of dosing. Patients will be stabilized on 20 mg of atorvastatin for approximately 4 weeks in Period 1, which in the case of statins is predicted to be a sufficient amount of time to allow for achievement of pharmacodynamic steady state.

A dyslipidemic patient population (with TG ≤ 400 mg/dL, LDL-C level of ≥ 100 mg/dL and ≤ 190 mg/dL, if they have no or 1 cardiac risk factor according to NCEP guidelines, or ≤ 160 mg/dL, if they have 2 or more cardiac risk factors according to NCEP

guidelines) under statin-treated conditions has been selected for this investigation, so that MK-0859 may be assessed in a paradigm that will most closely approximate its anticipated clinical use.

CCI [redacted] The intent of the frequently administered meals is to raise and maintain triglyceride levels and keep diet induced fluctuations in lipid levels to a minimum.

3.1.2 Rationale for Dose Regimen and Endpoints

Dose Regimen

MK-0859

A dose of 100 mg of MK-0859, administered once-daily with the main meal of the day has been chosen for this study. The rationale for dosing with the main meal of the day is to improve subject compliance for this study and to reduce pharmacokinetic variability associated with meals. CCI [redacted]

Atorvastatin

Atorvastatin calcium (LIPITOR®) is a lipid-lowering agent that acts as an inhibitor of 3-hydroxy-3-methlglutaryl-coenzyme A (HMG-CoA) reductase, which catalyzes an early and rate-limiting step in the biosynthesis of cholesterol. LIPITOR® administration is associated with reductions in total cholesterol and LDL cholesterol and with increases in HDL cholesterol, all of which have been associated with reductions in cardiovascular morbidity and mortality.

A dose of 20 mg of atorvastatin has been selected for background statin therapy for several reasons. Atorvastatin is a commonly used and well-tolerated statin drug that is indicated for the treatment of dyslipidemia and the most frequently prescribed dose is 20 mg daily. Atorvastatin was used at this dose in the studies of CETP inhibition with torcetrapib that form the basis for our power assumptions for this study. In addition, Atorvastatin was used at a dose of 20 mg in Phase IIb studies of MK-0859 and therefore a great deal of clinical experience has been accrued already with this specific combination.

Atorvastatin is generally well tolerated however, like other HMG-CoA reductase inhibitors, has been associated with liver chemistry abnormalities such as increases in

transaminase levels which typically manifests following administration over the first 3 months of treatment rather than after single-dose administration. In addition, rare cases of rhabdomyolysis and associated sequelae have been reported with atorvastatin and other HMG-CoA reductase inhibitors during the initial months of therapy.

Refer to LIPITOR® package insert in protocol Attachments for detailed information.

Endpoints

It is anticipated that the administration of MK-0859 in combination with atorvastatin to dyslipidemic patients will be associated with a mean change in at least one of our primary endpoints, the production rate or fractional catabolic rate of apoB100 in LDL-C. We further predict that MK-0859 monotherapy will result in a significant increase in our secondary endpoint, the fractional catabolic rate of LDL apoB100. Tertiary endpoints include changes in the fractional catabolic rate of apoA-I in HDL-C after treatment with MK-0859 in combination with atorvastatin, as well as MK-0859 monotherapy. Additionally, we will investigate the effect of atorvastatin alone on the fractional catabolic rate of LDL apoB100 in comparison to placebo as a tertiary objective. We will also explore the effect of treatment with MK-0859 monotherapy and MK-0859 in combination with atorvastatin on the kinetics of apoB100 in VLDL and IDL, apoA-II in HDL-C, and apo(a) in Lp(a). Additional samples will be archived for possible determination of VLDL TG production rate and PCSK9 and CETP kinetics. Effects on concentrations and/or activities of LCAT, LPL and HL, as well as lipoprotein size will also be studied. Additional samples will be archived for possible determination of PCSK9, lathosterol and prebeta HDL concentrations, as well as CETP mass and activity.

ApoB100 and apoA-I will be isolated from lipoprotein fractions after ultracentrifugation using SDS PAGE. [redacted] Additional samples will be archived for possible determination of lipoprotein size by proton NMR.

3.1.3 Rationale for Subject/Patient Genetic Sample Collection

As part of this study, pharmacogenomic analysis may be performed on samples from appropriately consented patients/patients. The objective of collecting genetic samples in this study is to investigate the relationship between genetic make-up, and the way investigational therapies are absorbed, broken down and eliminated from the body, how they affect the body and how DNA relates to human disease.

3.2 STUDY PROCEDURES

3.2.1 Study Restrictions

3.2.1.1 Concomitant Medication

Patients currently being treated with a statin at the prestudy (screening) visit will be washed-off their treatment beginning up to 3 weeks prior to study drug administration in

Period 1 of the study until the poststudy visit. After the poststudy visit has been completed, patients can resume treatment with their previously prescribed statin medication according to instructions provided by the study investigator.

Use of any other prescription or nonprescription medication (including herbal remedies such as St. John's Wort) within approximately 14 days prior to administration of the initial dose in Period 1 of the study (or 5 half lives, whichever is longer) and throughout the study or the anticipated use of any prescription or nonprescription medications are prohibited, unless the study investigator can rationalize that the specific use of a prior medication is not clinically relevant within the context of the study.

Medications of particular concern are prescription and non prescription lipid modifying therapies including fibrates, niacin, ezetimibe, bile acid-sequestrants, fish oils, hormonal contraceptives, and glucocorticoids; and potent inhibitors or inducers of CYP3A4.

Certain concomitant medications (e.g., stable doses of beta-blockers; low dose thiazide diuretics; hormone replacement therapy; psyllium and thyroid hormone replacement therapy) will be allowed to treat comorbid conditions (e.g., hypertension, hypothyroidism, and menopause), at the discretion of the investigator. Refer to Section 2.3 for details. Acetaminophen and over-the-counter non-steroidal anti-inflammatory drugs will be permitted for minor ailments with the permission of the study investigator.

Concurrent therapy with any medication during the course of the protocol (after randomization) including both prescription and nonprescription drugs must first be discussed with the investigator and Sponsor Medical Monitor prior to administration, unless appropriate medical care necessitates that therapy should begin before the investigator and Sponsor Medical Monitor can be consulted.

Paracetamol/acetaminophen and over-the-counter non-steroidal anti-inflammatory drugs may be used for minor ailments without prior consultation with the Merck clinical monitor.

3.2.1.2 Diet

Patients will be asked to provide a 24 hour food recall at the prestudy (screening) visit. Study participants will be asked to follow the American Heart Association NCEP or similar diet throughout the study. If deemed necessary by the clinical investigator the subject will meet with a dietician who will provide guidelines for following the American Heart Association NCEP diet or a similar diet. Patients will be asked to follow these guidelines throughout the study and not make any major changes to their diet during the study. Prior to Visit 5 (Day 28; Period 1) and Visit 8 (Day 56; Period 2) patients will be asked to complete a 3 day food record provided by the investigator. Three day food records should be reviewed upon receipt for accuracy and completion. Analysis of the 3-day food record will be conducted on an individual basis at the discretion of the investigator.

Patients must fast from all food and drink, except water, for 12 hours prior to laboratory safety tests and blood samples for the lipid panel, plasma lipoproteins, LCAT activity, HL and LPL analysis, and archive samples for: lipoprotein size, PCSK9, lathosterol and prebeta HDL concentration, and CETP mass and activity. All witnessed oral doses will be administered with approximately 240 mL of water.

During Visit 5 (Day 28, Period 1) and Visit 8 (Day 56, Period 2) patients will receive a standard meal in the evening. Patients will then fast, except from water until the administration of small iso-caloric meals (~ 18% fat) every 2 hours for 30 hours.

Fruit Juice Restrictions

Patients will refrain from consumption of grapefruit juice, grapefruits and grapefruit products beginning approximately 2 weeks prior to administration of the initial dose of study drug, throughout the study (including the washout interval between treatment periods) and until the poststudy visit.

3.2.1.3 Alcohol/Caffeine/Smoking/Activity

Alcohol

Patients will refrain from consumption of alcohol for 24 hours prior to Visit 5 (Day 28, Period 1) and Visit 8 (Day 56, Period 2) [prior to the beginning of the kinetic assay in each period] through the completion of the visit; and for 24 hours prior to Visit 2 (Day -2; Period 1), Visit 4 (Day 21; Period 1) and Visit 7 (Day 28; Period 2) and the pre- and post-study visits.

At all other times, alcohol consumption is limited to no more than approximately 2 alcoholic beverages or equivalent (beer [12 oz.], wine [5 oz] or distilled spirits [1.5 oz]) per day. Patients should be encouraged not to make major changes in their alcohol consumption for the duration of the study.

Caffeine

Patients will be allowed to consume up to two caffeinated beverages per day during Visit 5 (Day 28, Period 1) and Visit 8 (Day 56, Period 2) [prior to the beginning of the kinetic assay in each period] through the completion of the visit; and for 24 hours prior to Visit 2 (Day -2; Period 1), Visit 4 (Day 21; Period 1), Visit 7 (Day 28; Period 2) and the pre- and post-study visits.

At all other times, caffeinated beverages will be limited to no more than 6 units per day amounts (>6 units: 1 unit=120 mg of caffeine).

Activity

Patients should continue any activity he/she was accustomed to prior to entering the study. The subject must avoid all unusual, unaccustomed, or strenuous exercise or activity for the duration of the study and follow-up period. Activities to avoid include but

are not restricted to strenuous or unaccustomed exercise such as body building training, marathon training, and intense bicycling.

Smoking

Smoking is not permitted during the study.

Pregnancy and Contraception

Women of childbearing potential can be enrolled. However, 2 acceptable methods of barrier contraception must be used beginning at least 2 weeks prior to administration of the initial dose of study drug in Period 1, throughout the study until the poststudy visit. Acceptable methods of birth control are abstinence, or 2 of the following: intrauterine device (IUD-with or without local hormone release), diaphragm, spermicides, cervical cap, contraceptive sponge, and condoms.

Patients must be completely informed of the unknown risks of pregnancy and agree not to become pregnant during the time they are participating in this study.

Hormonal based contraceptives are not allowed as a method of birth control in this study.

Pregnancy Testing

Female patients of childbearing potential will be tested for serum β-human chorionic gonadotropin (hCG) at prestudy and poststudy visits, and for urine β-human chorionic gonadotropin (hCG) in Visit 4, Visit 5, Visit 7 and Visit 8. In the case of a positive or borderline serum β-hCG pregnancy test at the prestudy visit, the subject must not enter the study; in the case of a positive or borderline urine β-hCG pregnancy test during the study, the pregnancy test should be repeated and confirmed positive. If the pregnancy has been confirmed the subject must be discontinued from the study immediately and the Merck clinical monitor must be contacted within 24 hours. The site will contact the subject for SPONSOR updates until the pregnancy has been terminated or completed. The outcome of the pregnancy will be reported to the SPONSOR without delay and within 24 hours if the outcome is a serious adverse experience (e.g., death, abortion [spontaneous or elective], congenital anomaly, birth defect, or other disabling or life-threatening complication to the mother or newborn).

3.2.2 Procedures

Study procedures should be completed as close to the prescribed/scheduled time as possible.

The exact time at which a procedure is performed must be recorded on the case report forms. Any nonscheduled procedures required for urgent evaluation of safety concerns take precedence over all routine scheduled procedures.

See the Study Flow Chart in the Synopsis section for a complete outline of all study procedures.

Weight

Body weight will be measured using a standardized scale, with regular calibration. The same scale should be used for the duration of the study. Weight must be measured after an overnight fast, after voiding, with shoes and jacket off.

Vital Sign Measurements

Patients should be resting in a semi-recumbent position for at least 10 minutes prior to having vital sign measurements obtained. Semi-recumbent vital signs will include heart rate, blood pressure, respiratory rate, and temperature.

12-Lead ECG

Special Care must be taken for proper lead placement. Men should be shaved as necessary for proper lead placement. Women must remove their bras prior to lead placement.

Patients should be resting in a semi-recumbent position for at least 10 minutes prior to having ECG readings obtained.

Laboratory Safety Tests, Lipid Panel and Plasma Lipoproteins (Appendix 6.2)

Samples for the laboratory safety tests, lipid panel and plasma lipoproteins will be obtained after a 12-hour fast.

LCAT Activity Assay

LCAT activity will be measured on plasma by thin layer chromatography measuring the rate of cholesteryl ester formation. The site will follow the site SOP for conduction of assay. A copy of the site SOP for the LCAT activity assay will be maintained in the Investigator Trial files for this study.

Hepatic Lipase (HL) and Lipoprotein Lipase (LPL)

Blood samples will be obtained at the end of each kinetic assay (48 hours post start of the kinetic assay). Heparin (60 units/kg body weight) will be injected in the patient. Ten minutes after heparin infusion, 5 mL of blood will be collected and processed for the measurement of HL and LPL concentration and activity. Detailed procedures are provided in site SOPs. A copy of the site SOP for the conduct of the assay will be maintained in the Investigator Trial files for the study.

Lipoprotein Kinetic Assays

A brief summary of the procedures is in Appendix 6.8. The lipoprotein kinetic assays will be conducted per site specific SOPs. A copy of the site SOP for the lipoprotein kinetic assays will be maintained in the Investigator Trial files for this study.

Genetic Samples

Laboratory supplies and instructions for the labeling, packaging and overnight shipment of samples for genetic analysis will be provided by the central laboratory. The blood samples will be shipped the same day, at ambient temperature, to the central laboratory, according to the instructions provided by the central laboratory.

Archive Samples

Lipoprotein Size by NMR

Archive samples will be obtained after a 12 hour fast for possible determination of lipoprotein size by NMR. Procedures for sample preparation, storage and shipment are provided in an operations manual to the investigators.

Lathosterol Concentration and CETP Mass

Archive samples for possible determination of lathosterol concentration and CETP mass will be obtained after a 12 hour fast. Procedures for sample preparation, storage and shipment are provided in an operations manual to the investigators.

Prebeta HDL

Archive samples for possible determination of prebeta HDL will be obtained after a 12-hour fast Procedures for sample preparation, storage and shipment will be per site specific SOP and will be maintained in the Investigator Trial files for this study.

PCSK9 Concentration and CETP Activity

Archive samples for possible determination of PCSK9 concentration and CETP activity will be obtained after a 12-hour fast. Procedures for sample preparation, storage and shipment are in Appendices 6.5 and 6.6, respectively.

PCSK9 and CETP Kinetics

Archive samples will be obtained at each time point during the kinetics assay to support possible future investigations of PCSK9 and CETP kinetics. Storage and shipment procedures will be provided in an operations manual to the investigators.

Plasma for MK-0859 Concentration

Archive plasma samples for MK-0859 concentration will be collected at the timepoints specified in the Study Flow Chart. Procedures for sample preparation, storage and shipment are in Appendix 6.7.

Subject Domiciling

During the lipoprotein kinetics assays patients will be required to stay in the CRC for 2 consecutive overnight stays. Patients can be discharged from the CRC following the 24-hour blood draw for lipid turnover. Patients should report to the CRC for the 48 hour blood draw. All other clinic visits will be outpatient visits.

Dispensing of Study Drug

All study drug bottles will be dispensed by the site. Patients will be dispensed study drug at Visit 3 and Visit 6. Extra study drug supplies will be allotted in the bottle, in the event that the subject's next visit is outside of the protocol specified visit window.

Study Visit Scheduling

Every effort should be made to have the subject return to the CRC on the scheduled day of their visit. However, if due to extenuating circumstances the subject cannot keep an appointment, a 7-day window after the scheduled visit will be allowed. In this circumstance, the site will record the actual time and date of the rescheduled visit as it occurs.

3.2.2.1 Prestudy (Screening) (Visit 1)

Up to approximately 8 weeks (2 months) prior to study start, potential patients will be evaluated to determine whether they fulfill the entry requirements listed in Sections 2.2 and 2.3. The investigator or designate will discuss with the potential subject the nature of the study, its risks, requirements, and its restrictions.

All patients will be given a card, at the time of screening, identifying them as participants in a research study. The card will contain contact information (including direct telephone numbers) to be utilized in the event of an emergency.

Written informed consent will be obtained. After the patients signs the consent form, a unique baseline number should be assigned for identification purposes. Procedures to be conducted at the prestudy visit are given in Table 3-1.

Table 3-1

Prestudy Visit

Informed consents[†]
Medical history (including prior therapies)
Serum FSH test or serum β-hCG (if applicable)
Physical examination
Electrocardiogram (12-lead)
Weight
Height
Vital Signs (HR, BP, RR, temperature)
Laboratory safety tests
Lipid Panel
24-hour food recall
[†] Obtained prior to any study mandated procedures (consents for the main study, and genomic analysis).

3.2.2.2 Washout Period (Visit 2)

Patients who are taking statins during the screening visit will undergo a 2-week washout period prior to the randomization. Two days prior to randomization patients will report to the CRC (Visit 2) for a lipid panel to determine if their lipid levels meet the inclusion criteria. If the lipid levels are not within the specified range, patients will be allowed an additional 1 week washout. Statin-naïve patients will not undergo the washout period and can be randomized directly into the study if they otherwise meet all in inclusion criteria specified in Section 2.2.

3.2.2.3 Period 1: Day 1 Randomization (Visit 3)

Eligible patients will be randomized to blinded treatment and will receive a unique allocation number. Patients will be given the study medication to be taken at home with the dosing instructions. Patients will dose at home with the evening meal. The clinical staff will call the patients once or twice during the dosing interval to review the concomitant therapies/medical history/adverse experience as well as to check dosing compliance and remind patients of dosing instructions.

Period 1: Day 21 (Visit 4)

Visit 4 should occur approximately 1 week prior to the start of the kinetic assay in Period 1. Patients will report to the CRC having fasted for 12-hours. Laboratory safety tests, including a urine pregnancy test, if applicable, and a lipid panel will be taken during this visit. Patients will be given a 3-day food record to complete prior to reporting to the CRC for Visit 5.

Period 1: Day 28 through Day 31 (Visit 5 and Visit 6)

After 28 days of dosing (maximum 35 days) patients will undergo a lipoprotein kinetic assay. Patients will fast for 12 hours prior to reporting to the CRC on the morning of Day 28 for fasting blood draws. At the discretion of the investigator the patient can leave the CRC following the morning procedures and return to the CRC in the evening to begin the lipoprotein kinetic assay. Patients will then remain in the CRC until the completion of the 24 hour blood draw for lipoprotein kinetics, and should return to the CRC for the 48 hour blood draw. At the discretion of the investigator patients may be requested to remain in the CRC longer. Procedures to be conducted are outlined in Table 3-2.

Table 3-2

Procedures for Visit 5[†] and Visit 6 (Day 28 to Day 31)

Procedure	Time Relative to Drug Administration
Review study drug compliance	Morning of Day 28
Physical exam	Morning of Day 28
ECG	Morning of Day 28
Weight	Morning of Day 28
Vital signs	Morning of Day 28
Genetic Sample for archive	Morning of Day 28
Plasma lipoproteins and archive samples for lipoprotein size by NMR	Morning of Day 28
Lipid Panel	Morning of Day 28
Urine pregnancy Test	Morning of Day 28
Samples for LCAT activity assay	Morning of Day 28
PCSK9, Lathosterol and prebeta HDL for archive	Morning of Day 28
MK-0859 for archive	Morning of Day 28
CETP (mass and activity) for archive	Morning of Day 28
Study drug administration	Evening of Day 28 through Day 30
Standard meal	Evening of Day 28
Isocaloric meals[§]	Day 29: Starting at about 8 hours prior to the start of the kinetics assay, every 2 hours for 30 hours
[redacted] [‡]	Morning of Day 29
Blood for lipoprotein kinetic assay; archive samples for PCSK9 and CETP kinetics	Day 29: 0, 20, and 40 minutes and 1, 2, 4, 6, 8, 10, 12, 14, 15, 15.5, 16, 18, 21, 24, and 48 hours
Hepatic lipase and lipoprotein lipase analyses	Morning of Day 31 (48 hours post infusion)

† Visit 5 in Period 1 can occur between Days 28 and 35 (inclusive) of Period 1; subsequent procedures and days will be adjusted accordingly.

‡ [redacted]

§ Patients will be fed a small meal every 2 hours for 30 hours (iso-caloric meal of ~ 18% fat content).

Period 2: Day 1 (Visit 6)

There will be no washout between Period 1 and Period 2; therefore Day 1 of Period 2 is the same day as Day 31 of Period 1. While at the CRC for Period 1 Day 31 procedures patients will be given their study medication for Period 2. In Period 2, patients will dose at home with the evening meal. The clinical staff will call the patients once or twice during the dosing interval of each treatment to review the concomitant therapies/medical history/adverse experience as well as to check dosing compliance and remind patients of dosing instructions.

Period 2—Day 28 (Visit 7)

Visit 7 should occur approximately 28 days after the start of dosing in Period 2. Patients will report to the CRC in the morning having fasted for at least 12 hours. Procedures to be conducted during Visit 7 are listed in Table 3-3.

Table 3-3

Period 2, Visit 7 (Day 28) Procedures

Procedure
Review study drug compliance
Review adverse experiences
Physical Exam
Vital signs
12-lead ECG
Laboratory Safety Tests
Urine pregnancy Test
Lipid panel
Dispense 3-day food record

Period 2–Day 29 to 55

The patients will dose at home following the dosing instructions. The patients will be contacted periodically by the clinic staff to monitor concomitant therapies/medical history/adverse experience and drug compliance. The exact time of dosing on Day 55 will be recorded in the CRFs.

Period 2-Day 56 through Day 59 (Visit 8 and Visit 9)

After 56 days of dosing (maximum 63 days), patients will undergo a lipoprotein kinetic assay. Patients will fast for 12 hours prior to reporting to the CRC on the morning of Day 56 for fasting blood draws. At the discretion of the investigator, patients can leave the CRC following the morning procedures and return to the CRC in the evening to begin the lipoprotein kinetic assay. Patients will then remain in the CRC until the completion

of the 24 hour blood draw for lipoprotein kinetics, and should return to the CRC for the 48 hour blood draw. At the discretion of the investigator, patients may be requested to remain in the CRC longer. Visit 8 and Visit 9 procedures are in Table 3-4.

Table 3-4

Procedures for Visit 8† and Visit 9 (Days 56-59)

Procedure	Time Relative to Drug Administration
Review study drug compliance	Morning of Day 56
Vital Signs	Morning of Day 56
Laboratory Safety Tests	Morning of Day 56
Urine Pregnancy Test	Morning of Day 56
ECG	Morning of Day 56
Lipid Panel	Morning of Day 56
Physical exam	Morning of Day 56
Weight	Morning of Day 56
Plasma lipoproteins and archive samples for lipoprotein size by NMR	Morning of Day 56
Samples for LCAT activity assay	Morning of Day 56
PCSK9, Lathosterol and prebeta HDL for archive	Morning of Day 56
CETP (mass and activity) for archive	Morning of Day 56
MK-0859 for archive	Day 56 predose‡, 16 and 20 hours postdose; Day 57 predose‡, 2, 4, 8, 12, 16, 40 and 240‡ hours postdose Day 57
Standard Meal	5 PM Day 55
Study drug administration	5 PM Day 56 and Day 57
Isocaloric meals‖	Day 57: Starting at about 8 hours prior to the start of the kinetics assay, every 2 hours for 30 hours
[redacted]§	Morning of Day 57
Blood for lipid kinetic assay; archive samples for PCSK9 and CETP kinetics	Day 57: 0, 20, and 40 minutes and 1, 2, 4, 6, 8, 10, 12, 14, 15, 15.5, 16, 18, 21, 24, and 48 hours
Hepatic lipase and lipoprotein lipase analyses	Morning of Day 59 (48 hours post infusion)

† Visit 8 in Period 2 can occur between Days 56 and 63 (inclusive) of Period 2; subsequent procedures and days will be adjusted according to the actual day of the visit.

‡ The predose samples should be obtained anytime within 1 hour of dosing. The 240 hour post dose sample will be obtained during the poststudy visit.

§ [redacted]

‖ Patients will be fed a small meal every 2 hours for 30 hours (iso-caloric meal of ~ 18% fat content).

3.2.2.4 Poststudy

Subjects will be required to return to clinic approximately 14 days after the last dose of study drug for the poststudy visit. If the poststudy visit occurs less than 14 days after the last dose of study drug, a subsequent follow-up phone call should be made to determine if any adverse experiences have occurred since the poststudy clinic visit

If a subject discontinues at any time during the course of the study, the subject may be asked to return to the clinic (or be contacted) for a poststudy visit (approximately 14 days after the last dose of study drug) to have the applicable procedures conducted. However, the investigator may decide to perform the poststudy procedures at the time of discontinuation or as soon as possible after discontinuation. If the poststudy visit occurs prior to 14 days after the last dose of study drug, the investigator should perform a follow up phone call to determine if any adverse experiences have occurred since the poststudy clinic visit.

Following this, participation in this study will be complete.

Poststudy evaluations will include those listed in Table 3-5.

Table 3-5

Poststudy Procedures

Physical examination
Electrocardiogram (12-lead)
Weight
Vital Signs
MK-0859 for archive
Laboratory safety tests
Serum pregnancy test

3.2.2.5 Volume of Blood Drawn During the Study

The total blood volume withdrawn from any single subject will not exceed the maximum allowable volume during their participation in the entire study, see Appendix 6.4.

3.2.3 Procedures

3.2.3.1 Informed Consent

3.2.3.1.1 General Informed Consent

The investigator must obtain documented consent from each potential patient in biomedical research or when an investigational drug is administered to the patient in a clinical study, prior to any study related procedures being performed.

Consent must be documented by the patient's dated signature on a Consent Form along with the dated signature of the person conducting the consent discussion. A copy of the signed and dated consent form should be given to the subject before participating in the trial.

3.2.3.1.2 Consent and Collection of Specimens for Genetic Analysis

During this study, a separate signed informed consent will be administered for collecting a whole blood specimen for potential future genetic research. Only those patients who have consented to having this genetic sample collected may have this blood sample drawn. The investigator or designate is responsible for explaining and verifying the subject's consent before obtaining such blood samples. At the time of sample collection, the investigator's staff member and a witness from the investigator's staff should verify that the subject has signed consent and the correct subject-specific label is placed on the genetic sample, and then both the investigator's staff member and witness initial and date the left-side of the Genetic Sample Label. It should be explained to the subject that giving the blood sample for genetic information is entirely optional for the subject and participation in the associated clinical study is not dependent giving these samples. The approval of the consent form for analysis and the associated protocol procedures (e.g., collection of a blood sample) may, in some cases, proceed independently through Institutional Review Boards, Ethical Review Boards, Independent Ethical Committees, Privacy Committees, etc. from the associated clinical study. In such cases, the clinical study protocol should proceed ahead independently of the above; donation of the genetic sample, once approved, may then be deferred to subsequent study visits. In cases where the IRB/ERC approval for the donation of a sample for genetic analysis is denied or is not accomplished prior to the completion of the clinical study, samples for genetic analysis will not be collected.

3.2.3.2 Assignment of Baseline Number/Screening

Each patient screened will be assigned a baseline or screening number. The number is assigned to the subject upon signing the consent form to identify the subject for all procedures that occur prior to randomization. This number is composed of the Merck study number followed by a 4 digit patient number. For example, study site 0001 will assign a baseline number of 00010001 to the first patient screened. Each patient will be assigned only one baseline number and each baseline or screening number will be assigned to only one subject. Patients who are screened multiple times will retain the same baseline number.

3.2.3.3 Randomization/Allocation

Each subject will be assigned an allocation number at the time of randomization. The allocation number will be used to identify the subject for all procedures occurring after randomization.

A single patient/subject cannot be assigned more than 1 allocation number.

In a situation where rerandomization of the subjects/patients is planned (e.g., study extension periods), the rerandomization is done based on a new allocation schedule, however each subject/patient retains his/her original allocation number. Only the treatment regimen associated with the rerandomization period or phase may change.

3.2.3.4 Blinding/Unblinding

In the event of an emergency, the patient's treatment can be unblinded. **Except for an emergency when the information could influence medical care, the investigator should not unblind a subject's treatment without prior approval from Merck.** If unblinding of the investigational product(s) should occur (e.g., accidental unblinding, emergency unblinding for a serious adverse experience), the investigator must promptly document the circumstances and immediately notify the Merck Clinical Monitor. If unblinding occurs, only the principal investigator or delegate and the respective patient's code should be unblinded. Site personnel and Merck personnel directly associated with the conduct of the study should not be unblinded. The unblinding should be documented in the unblinding log provided by the Merck.

3.2.3.5 Discontinuation/Withdrawal from Study

Patients/patients may withdraw at any time or be dropped from the study at the discretion of the investigator should any untoward effects occur. In addition, a subject/patient may be withdrawn by the investigator or the SPONSOR if he/she violates the study plan or for administrative and/or other safety reasons. The investigator or study coordinator must notify the SPONSOR immediately when a subject/patient has been discontinued/ withdrawn due to an adverse experience (telephone or FAX). When a subject/patient discontinues/withdraws prior to study completion, all applicable activities scheduled for the final study visit should be performed at the time of discontinuation. Any adverse experiences which are present at the time of discontinuation/withdrawal should be followed in accordance with the safety requirements outlined in Section 3.4 SAFETY MEASUREMENTS - DETAILS.

Patients/patients who donate a blood sample for future genetic analyses may request that their sample be removed from storage and destroyed in accordance with the terms outlined in the informed consent for genetic analyses. Patients/patients should be informed that withdrawal from the main study does not cause the withdrawal and destruction of the genetic sample. Requests for withdrawal and destruction of the genetic sample should be made in writing to the investigator.

Subjects/patients who discontinue from the study will be replaced as required for the study to meet its objectives. The decision to remove a subject/patient and to replace dropouts will be made jointly by the investigator, SPONSOR Clinical Monitor, and SPONSOR study statistician. The replacement will generally receive the same treatment or treatment sequence (as appropriate) as the allocation number replaced. Both the replacement and originally allocated number will be unique numbers.

Biological specimens obtained prior to subject/patient discontinuation can be analyzed unless consent is withdrawn.

3.3 EFFICACY/PHARMACOKINETIC/IMMUNOGENICITY, ETC. MEASUREMENTS

3.3.1 Pharmacodynamic Measurements

Blood samples will be collected at 0 (pre-bolus), 20 and 40 minutes, and 1, 2, 4, 6, 8, 10, 12, 14, 15, 15.5, 16, 18, 21, 24, and 48 hours post-infusion for the isolation of plasma VLDL, IDL, LDL, and HDL using ultracentrifugation. ApoB100, apoA-I, apoA-II and apo(a) will be isolated from lipoprotein fractions using SDS PAGE. [redacted]

Production and fractional clearance rates of apoB100 in LDL, VLDL and IDL, apo A-I and apoA-II in HDL, and apo(a) in Lp(a) will be calculated. Comparisons between groups will be performed for percent conversion of VLDL apoB100 to IDL apoB100, VLDL apoB100 to LDL apoB100, and IDL apoB100 to LDL apoB100. Samples will be archived for potential determination of VLDL TG production, and PCSK9 and CETP kinetics.

Plasma will be analyzed to determine the concentrations of lipoprotein lipase (LPL), hepatic lipase (HL) and apolipoproteins, as well as activities of LPL, HL and lecithin-cholesterol acyltransferase (LCAT). Additional samples will be archived for possible determination of PCSK9, lathosterol and prebeta HDL concentrations and lipoprotein size by NMR. Samples for determination of CETP activity and mass will also be archived.

3.3.2 Pharmacokinetic Measurements-Archive Plasma for MK-0859

Archive Plasma for MK-0859

The decision as to which plasma and/or urine samples collected will be assayed for evaluation of pharmacokinetics/pharmacodynamics will be collaboratively determined by the Departments of Clinical DM/PK and Clinical Pharmacology (e.g., samples at lower doses may not be assayed if samples at higher doses reveal undetectable drug concentrations). If indicated, these samples may also be assayed and/or pooled for assay in an exploratory manner for metabolites and/or additional pharmacodynamic markers.

At the time points listed in the Study Flow Chart, blood will be collected for the determination of MK-0859 Appendix 6.7 provides instructions for sample collection, storage and shipping procedures.

3.3.3 Blood for Pharmacogenomic Analysis

Laboratory supplies and instructions for the labeling, packaging and overnight shipment of samples for genetic analysis will be provided by the genetic sample bank. Approximately 10 mL of whole venous blood will be collected into EDTA vacuum tubes from each patient consenting to genetic analysis. The blood samples will be shipped the same day, at ambient temperature, to the genetic sample bank, according to the

instructions provided by the genetic sample bank. It is extremely important to remember that the genetic sample tube be labeled only with the barcode label containing the GIC.

3.3.4 Medication Compliance

Adherence to study drug treatment will be assessed by patient report and confirmed by tablet count of each bottle at Visit 5 (Day 28, Period 1), Visit 7 (Day 28, Period 2) and Visit 8 (Day 56, Period 2). At the discretion of the investigator, the site may call patients once or twice on any day to monitor dosing compliance. The site will contact the patient the day prior to Visit 8 (Day 55) to record the exact time of dosing on that day. Every effort should be made to maintain adherence to study drug dosing per protocol.

3.4 SAFETY MEASUREMENTS

3.4.1 Clinical and Laboratory Measurements for Safety

Safety will be monitored throughout the study by repeated clinical and laboratory evaluation including vital signs, physical exam, 12-lead ECG, laboratory safety parameters (hematology, chemistry, and urinalysis), plasma lipids (total cholesterol, HDL, LDL and triglycerides) and serum/urine β-hCG at appropriate time points as specified in the Study Flow Chart.

3.4.2 Recording Adverse Experiences

An adverse experience is defined as any unfavorable and unintended change in the structure, function, or chemistry of the body temporally associated with the use of the SPONSOR’s product, whether or not considered related to the use of the product. Any worsening (i.e., any clinically significant adverse change in frequency and/or intensity) of a preexisting condition which is temporally associated with the use of the SPONSOR’s product, is also an adverse experience.

Changes resulting from normal growth and development which do not vary significantly in frequency or severity from expected levels are not to be considered adverse experiences. Examples of this may include, but are not limited to, teething, typical crying in infants and children, and onset of menses or menopause occurring at a physiologically appropriate time.

Adverse experiences may occur in the course of the use of a Merck product in clinical studies or within the follow-up period specified by the protocol, or prescribed in clinical practice, from overdose (whether accidental or intentional), from abuse, and from withdrawal.

Adverse experiences may also occur in screened patients/patients during any preallocation baseline period as a result of a protocol-specified intervention including washout or discontinuation of usual therapy, diet, placebo treatment, or a procedure.

Such events will be recorded at each examination on the Adverse Experience Case Report Forms/Worksheets.

3.4.3 Definition of an Overdose for This Protocol

The patient has taken, accidentally or intentionally, any drug administered as part of the protocol, and exceeding the dose as prescribed by the protocol. It is up to the investigator or the reporting physician to decide whether a dose is to be considered an overdose, but Merck personnel have the authority to upgrade the determination, if deemed appropriate. Before upgrading a report, it is necessary for Merck personnel to communicate with the investigator and reach a consensus.

3.4.3.1 Reporting of Overdose to SPONSOR

If an adverse experience(s) is associated with ("results from") the overdose of test drug or vaccine, the adverse experience(s) is reported as a serious adverse experience, even if no other criteria for serious are met.

If a dose of test drug or vaccine meeting the protocol definition of overdose is taken without any associated clinical symptoms or abnormal laboratory results, the overdose is reported as a non-serious Event of Clinical Interest (ECI), using the terminology "accidental or intentional overdose without adverse effect."

All reports of overdose with and without an adverse experience must be reported within 24 hours to one of the individuals listed on the sponsor contact information page found in the Administrative Binder.

3.4.4 Reporting of Pregnancy to SPONSOR

Although not considered an adverse experience, it is the responsibility of investigators or their designees to report any pregnancy in a subject/patient (spontaneously reported to them) which occurs during the study or within 14 days of completing the study. All subjects/patients who become pregnant must be followed to the completion/termination of the pregnancy. If the pregnancy continues to term, the outcome (health of infant) must also be reported to one of the individuals listed on the SPONSOR Contact Information page found in the Administrative Binder.

3.4.5 Immediate Reporting of Adverse Experiences to the SPONSOR

3.4.5.1 Serious Adverse Experiences

Any serious adverse experience, including death due to any cause, which occurs to any subject/patient entered into this study or within 14 days following cessation of treatment or within the established off therapy follow-up period for safety described in the protocol, whether or not related to the investigational product, must be reported within 24 hours to one of the individual(s) listed on the contact information page.

Additionally, any serious adverse experience considered by an investigator who is a qualified physician to be possibly, probably, or definitely related to the investigational product that is brought to the attention of the investigator at any time outside of the time period specified in the previous paragraph also must be reported immediately to one of

the individuals listed on the sponsor contact information page found in the administrative binder.

All patients/patients with serious adverse experiences must be followed up for outcome.

3.4.6 Evaluating Adverse Experiences

Refer to Table 3-6 for instructions in evaluating adverse experiences.

Table 3-6

An investigator who is a qualified physician, will evaluate all adverse experiences as to:

**Maximum Intensity**	**Mild**	awareness of sign or symptom, but easily tolerated (for pediatric studies, awareness of symptom, but easily tolerated)
	**Moderate**	discomfort enough to cause interference with usual activity (for pediatric studies, definitely acting like something is wrong)
	**Severe**	incapacitating with inability to work or do usual activity (for pediatric studies, extremely distressed or unable to do usual activities)
**Seriousness**	A serious adverse experience is any adverse experience occurring at any dose that:	
	†**Results in death**; or	
	†**Is life threatening; or** places the subject/patient, in the view of the investigator, at immediate risk of death from the experience as it occurred [Note: This does not include an adverse experience that, had it occurred in a more severe form, might have caused death.]; or	
	†**Results in a persistent or significant disability/incapacity** (substantial disruption of one's ability to conduct normal life functions); or	
	†**Results in or prolongs an existing inpatient hospitalization** (hospitalization is defined as an inpatient admission, regardless of length of stay, even if the hospitalization is a precautionary measure for continued observation. (Note: Hospitalization [including hospitalization for an elective procedure] for a preexisting condition which has not worsened does not constitute a serious adverse experience.); or	
	†**Is a congenital anomaly/birth defect** (in offspring of subject/patient taking the product regardless of time to diagnosis); or	
	**Is a cancer;** or	
	**Is an overdose** (Whether accidental or intentional.) Any overdose whether or not associated with an adverse experience must be reported within 24 hours to one of the individuals on the Contact Information Page found in the Administrative Binder.	
	**Other important medical events** that may not result in death, not be life threatening, or not require hospitalization may be considered a serious adverse experience when, based upon appropriate medical judgment, the event may jeopardize the subject/patient and may require medical or surgical intervention to prevent one of the outcomes listed previously (designated above by a †).	
**Duration**	Record the start and stop dates of the adverse experience. If less than 1 day, indicate the appropriate length of time and units	
**Action taken**	Did the adverse experience cause the test drug to be discontinued?	
**Relationship to test drug**	Did the test drug cause the adverse experience? The determination of the likelihood that the test drug caused the adverse experience will be provided by an investigator who is a qualified physician. The investigator's signed/dated initials on the source document or worksheet, that supports the causality noted on the AE form, ensures that a medically qualified assessment of causality was done. This initialed document must be retained for the required regulatory time frame. The criteria below are intended as reference guidelines to assist the investigator in assessing the likelihood of a relationship between the test drug and the adverse experience based upon the available information.<br>**The following components are to be used to assess the relationship between the test drug and the AE**; the greater the correlation with the components and their respective elements (in number and/or intensity), the more likely the test drug caused the adverse experience (AE):	
	**Exposure**	Is there evidence that the subject/patient was actually exposed to the test drug such as: reliable history, acceptable compliance assessment (pill count, diary, etc.), expected pharmacologic effect, or measurement of drug/metabolite in bodily specimen?
	**Time Course**	Did the AE follow in a reasonable temporal sequence from administration of the test drug?<br>Is the time of onset of the AE compatible with a drug-induced effect?
	**Likely Cause**	Is the AE not reasonably explained by another etiology such as underlying disease, other drug(s)/vaccine(s), or other host or environmental factors

**Relationship to test drug (continued)**	**The following components are to be used to assess the relationship between the test drug and the AE: (continued)**	
	**Dechallenge**	Was the dose of test drug discontinued or reduced?<br>If yes, did the AE resolve or improve?<br>If yes, this is a positive dechallenge. If no, this is a negative dechallenge.<br>(Note: This criterion is not applicable if: (1) the AE resulted in death or permanent disability; (2) the AE resolved/improved despite continuation of the test drug; or (3) the study is a single-dose drug study.)
	**Rechallenge**	Was the subject/patient reexposed to the test drug in this study?<br>If yes, did the AE recur or worsen?<br>If yes, this is a positive rechallenge. If no, this is a negative rechallenge.<br>(Note: This criterion is not applicable if: (1) the initial AE resulted in death or permanent disability, or (2) the study is a single-dose drug study.)<br>NOTE: IF A RECHALLENGE IS PLANNED FOR AN ADVERSE EVENT WHICH WAS SERIOUS AND WHICH MAY HAVE BEEN CAUSED BY THE TEST DRUG, OR IF REEXPOSURE TO THE TEST DRUG POSES ADDITIONAL POTENTIAL SIGNIFICANT RISK TO THE SUBJECT/PATIENT, THEN THE RECHALLENGE MUST BE APPROVED IN ADVANCE BY THE U.S. CLINICAL MONITOR AND THE INSTITUTIONAL REVIEW BOARD/INDEPENDENT ETHICS COMMITTEE.
	**Consistency with Study Drug Profile**	Is the clinical/pathological presentation of the AE consistent with previous knowledge regarding the test drug or drug class pharmacology or toxicology?
	The assessment of relationship will be reported on the case report forms/worksheets by an investigator who is a qualified physician according to his/her best clinical judgment, including consideration of the above elements.<br>**Use the following scale of criteria as guidance (not all criteria must be present to be indicative of a drug relationship).**	
	**Definitely related**	There is evidence of exposure to the test drug. The temporal sequence of the AE onset relative to administration of the test drug is reasonable. The AE is more likely explained by the test drug than by another cause. Dechallenge is positive. Rechallenge (if feasible) is positive. The AE shows a pattern consistent with previous knowledge of the test drug or test drug class.
	**Probably related**	There is evidence of exposure to the test drug. The temporal sequence of the AE onset relative to administration of the test drug is reasonable. The AE is more likely explained by the test drug than by another cause. Dechallenge (if performed) is positive.
	**Possibly related**	There is evidence of exposure to the test drug. The temporal sequence of the AE onset relative to administration of the test drug is reasonable. The AE could have been due to another equally likely cause. Dechallenge (if performed) is positive.
	**Probably not related**	There is evidence of exposure to the test drug. There is another more likely cause of the AE. Dechallenge (if performed) is negative or ambiguous. Rechallenge (if performed) is negative or ambiguous.
	**Definitely not related**	The subject/patient did not receive the test drug. **OR** Temporal sequence of the AE onset relative to administration of the test drug is not reasonable. **OR** There is another obvious cause of the AE.

3.4.7 SPONSOR Responsibility for Reporting Adverse Experiences

All adverse experiences will be reported to regulatory agencies, IRB/IECs, and investigators in accordance with all applicable global laws and regulations.

3.5 DATA ANALYSIS

The statistical analysis of the data obtained from this study will be conducted by, or under the direct auspices of, the Clinical Pharmacology Statistics Department in collaboration with the Drug Metabolism department, the Clinical Pharmacology Department of the Sponsor, and University of Pennsylvania.

If, after the study has begun, changes are made to the statistical analysis plan stated below, then these deviations to the plan will be listed, along with an explanation as to why they occurred, in the Clinical Study Report.

3.5.1 Hypotheses

Primary Hypothesis: The addition of MK-0859 to background atorvastatin therapy is associated with a decrease in PR of LDL apoB100. A true difference in LDL apoB100 PR obtained following Panel A, Period 2 compared to the LDL apoB100 PR obtained following Panel A, Period 1 (MK-0859 + atorvastatin versus atorvastatin) is expected.

Primary Hypothesis: The addition of MK-0859 to background atorvastatin therapy is associated with an increase in FCR of LDL apoB100. A true difference in LDL apoB100 FCR obtained following Panel A, Period 2 compared to the LDL apoB100 FCR obtained following Panel A, Period 1 (MK-0859 + atorvastatin versus atorvastatin) is expected.

Secondary Hypothesis: The administration of MK-0859 is associated with an increase in FCR of LDL apoB100. A true difference in LDL apoB100 FCR obtained following Panel B, Period 2 compared to the LDL apoB100 FCR obtained following Panel B, Period 1 (MK-0859 versus PBO) is expected.

Tertiary Hypothesis: The administration of MK-0859 is associated with a decrease in FCR of HDL apoA-I. A true difference in HDL apoA-I FCR obtained following Panel B, Period 2 compared to the HDL apoA-I FCR obtained following Panel B, Period 1 (MK-0859 versus PBO) is expected.

Tertiary Hypothesis: The addition of MK-0859 to background atorvastatin therapy is associated with a decrease in FCR of HDL apoA-I. A true difference in HDL apoA-I FCR obtained following Panel A, Period 2 compared to the HDL apoA-I FCR obtained following Panel A, Period 1 (MK-0859 + atorvastatin versus atorvastatin) is expected.

Tertiary Hypothesis: The administration of atorvastatin is associated with an increase in FCR of LDL apoB100. A true difference in LDL apoB100 FCR obtained following Panel A, Period 1 (atorvastatin 20 mg) compared to the LDL apoB100 FCR obtained following Panel B, Period 1 (PBO) is expected.

3.5.2 Variables and Time Points

The primary variables of interest are the production rate (PR) and fractional catabolic rate (FCR) of LDL apoB100. Additional endpoints of interest are as follows: 1) the pool size (PS) of LDL apoB100; 2) the kinetics (PR, FCR and PS) of apoA-I in HDL, apoA-II in HDL, apoB100 in VLDL, apoB100 in IDL and apo(a) in Lp(a); 3) the concentration of proteins involved in the modulation of VLDL, LDL, and HDL lipid composition, including lecithin-cholesterol acyltransferase (LCAT), lipoprotein lipase (LPL) and hepatic lipase (HL); and 4) the concentrations of apoA-I, apoA-II, apoB100, apoC-II, apoC-III, apoE, Lp(a); 5) the activities of lecithin-cholesterol acyltransferase (LCAT), LPL and HL; and 6) the percent conversions of VLDL apoB100 to IDL apoB100, VLDL apoB100 to LDL apoB100, and IDL apoB100 to LDL apoB100 (refer to Appendix 6.9).

Samples for the following variables will be archived: VLDL TG production rate, cholesteryl ester transfer protein (CETP; kinetics, concentration and activity), PCSK9 (kinetics and concentration), lathosterol, prebeta HDL and lipoprotein size by NMR for LDL, HDL and VLDL. These variables may also be included in the statistical analysis if samples are analyzed.

Safety endpoints will include all types of adverse experiences, laboratory tests, ECGs, vital signs and the concentrations of total cholesterol, HDL, LDL and triglycerides.

3.5.3 Approaches to Analyses

The following populations are defined for the analysis and reporting of data. All patients will be reported, and their data analyzed, according to the treatment(s) they actually received.

All Patients *as Treated* (APT) - All patients who received at least one dose of the investigational drug. This population will be used for assessments of safety and tolerability.

Per-Protocol (PP) – The set of data generated by the subset of patients who comply with the protocol sufficiently to ensure that these data will be likely to exhibit the effects of treatment, according to the underlying scientific model. Compliance covers such considerations as exposure to treatment, availability of measurements and absence of major protocol violations. Major protocol violators will be identified to the extent possible prior to unblinding by individuals responsible for data collection/compliance, and its analysis and interpretation. Any patients or data values excluded from analysis will be identified, along with their reason for exclusion, in the CSR. This population will be used for the PK and PD analyses.

3.5.4 Statistical Methods

Primary Hypotheses (LDL apoB100 PR & FCR)

The primary hypotheses will be addressed by analyzing both the production rate (PR) and the fractional catabolic rate (FCR) of LDL apoB100 with separate linear mixed effects models containing fixed effects for panel and treatment within panel, and a random effect

for subject within panel. Covariates for age and gender may also be included in the model. Summary statistics including point estimates, 95% confidence intervals (CIs) and between-treatment p-values (two-tailed) for the true mean differences (MK-0859 w/atorvastatin - atorvastatin) in LDL apoB100 PR and LDL apoB100 FCR will be calculated based on their respective models. The point estimate and CI for LDL apoB100 PR will be compared to the -1.4 mg·kg/d expected mean difference.

The 4A multiple testing procedure of Li and Mehrotra (2008) [1] will be followed in order to account for the disparity in statistical power for the two primary endpoints. If the p-value for the true mean difference (MK-0859 w/atorvastatin versus atorvastatin) in LDL apoB100 PR is statistically significant at the α_1=0.04 level, the corresponding true mean difference for LDL apoB100 FCR will be tested at the full α_2=0.05 level. However, if the true mean difference for LDL apoB100 PR is not significant at α_1=0.04, the true mean difference for LDL apoB100 FCR will be tested at an adjusted α_2 level based on both the observed p-value for PR and the correlation among the two endpoints. The primary hypothesis will be supported if the test for either endpoint is significant at their respective α level. That is, administration of MK-0859 on a background of atorvastatin therapy is associated with a significant change in LDL apoB100 turnover.

Secondary Hypothesis (LDL apoB100 FCR)

The secondary hypothesis and third tertiary hypothesis will be addressed by analyzing the fractional catabolic rate of LDL apoB100 with a linear mixed effects model containing fixed effects for panel and treatment within panel, and a random effect for subject within panel. Covariates for age and gender may also be included in the model. Summary statistics including point estimates, 95% CIs and between-treatment p-values (two-tailed) for the true mean differences (MK-0859 – placebo; atorvastatin - placebo) in LDL apoB100 FCR will be calculated based on this model. The contrast for the cross-panel comparison (i.e., atorvastatin versus placebo) will include the appropriate coefficients for the panel effects. The point estimate and CI for the comparison of MK-0859 versus placebo in LDL apoB100 FCR will be compared to the 0.065 pools/d expected mean difference. Similarly, the point estimate and CI for the comparison of atorvastatin versus placebo in LDL apoB100 FCR will be compared to the 0.20 pools/d expected mean difference.

Tertiary Hypotheses (HDL apoA-I FCR)

The first and second tertiary hypotheses will be addressed by an analyzing the fractional catabolic rate of HDL apoA-I using a similar linear mixed effects model as described above. Summary statistics including point estimates, 95% CIs and between-treatment p-values (two-tailed) for the true mean differences (MK-0859 w/atorvastatin – atorvastatin; MK-0859 – placebo) in HDL apoA-I FCR will be calculated based on this model. The point estimates and CIs will then be compared to their respective -0.048 and -0.033 pools/d expected mean differences.

The third tertiary hypothesis for comparison of atorvastatin versus placebo in LDL apoB100 FCR is addressed above.

Exploratory Analyses

Each of the exploratory endpoints listed in Section 3.5.2 will be analyzed with a similar linear mixed effects model as described above. Summary statistics including point estimates, 95% CIs and between-treatment p-values (two-tailed) for the true mean differences in these parameters will be reported. All contrasts for the cross-panel comparisons will include the appropriate coefficients for the panel effects. Descriptive statistics (mean and standard deviation, etc.) by treatment will also be provided.

A correlation analysis including all primary, secondary, tertiary and exploratory endpoints will be conducted to examine the relationships among these parameters. In addition to the Pearson correlation coefficient (ρ), a 95% confidence interval will also be calculated for each pair of endpoints. The upper limit of the 95% CI for ρ of LDL apoB100 PR versus FCR will be used to obtain the α_2 level for the 4A multiple testing method described above if the true mean difference (MK-0859 w/atorvastatin - atorvastatin) for LDL apoB100 PR is not significant at α_1=0.04.

Safety Analyses

Descriptive statistics and plots will be generated by panel for change (or percent change as appropriate) from prestudy of clinically appropriate safety laboratory parameters, vital signs, ECG parameters and the concentrations of total cholesterol, HDL, LDL and triglycerides.

3.5.5 Assumptions

Data will be examined for departures from the assumptions of the statistical model(s) as appropriate; e.g., heteroscedasticity, nonnormality of the error terms. Distribution-free methods may be used if a serious departure from the assumptions of the model(s) is observed, or suitable data transformations may be applied.

3.5.6 Multiplicity

This study has two primary comparisons: change in LDL apoB100 PR and change in LDL apoB100 FCR after administration of MK-0859 on a background of atorvastatin therapy versus atorvastatin alone. Success on either comparison is sufficient to declare a positive trial. Therefore, as outlined above, the method of Li and Mehrotra (2008) [1] will be followed to control the family-wise error rate for the primary hypotheses at α=0.05.

In addition to the primary comparisons, a large number of between-treatment comparisons will be conducted to address the secondary, tertiary and exploratory objectives of the study. In order to facilitate a balanced interpretation of these results, adjusted p-values will be reported in addition to the raw p-values. Using the two-stage method of Mehrotra

and Heyse (2004) [7], the false discovery rate (FDR) [8] will be controlled at 5%. For this purpose, three families of hypotheses are defined:

F1) hypothesis tests of the following endpoints: kinetics of apoB100 in LDL, VLDL and IDL, conversions of VLDL apoB100 to IDL apoB100, VLDL apoB100 to LDL apoB100, and IDL apoB100 to LDL apoB100, concentrations and activity of HL and LPL, and concentrations of apoB100, apoC-II, apoC-III and apoE

F2) hypothesis tests of the following endpoints: kinetics of apoA-I in HDL, kinetics of apoA-II in HDL, LCAT activity and concentrations of apoA-I and apoA-II

F3) hypothesis tests of the following endpoints: kinetics of apo(a) in Lp(a) and concentration of Lp(a)

for the following three sets of between-treatment differences:

D1) MK-0859 w/atorvastatin versus atorvastatin
D2) MK-0859 versus placebo
D3) MK-0859 w/atorvastatin versus MK-0859.

The "Double FDR" method will be implemented separately within each hypothesis family (F) using the three between-treatment differences (Ds) as the procedure subsets. Note that the Ds on some of the endpoints are expected to demonstrate no change (refer to Appendix 6.9). Since a multiplicity adjustment is inappropriate for these comparisons, the relevant endpoint for that D will be excluded from the Double FDR procedure.

3.5.7 Power Calculations

The variability estimates and detectable differences presented in the power calculations below were observed in [2] and from personal communication with PPD

Primary Hypotheses

Assuming a pooled, within-subject standard deviation of 1.94 mg·kg/d, N=30 subjects in Panel A, and a significance level of 0.04 (two-tailed), there is 96.0% probability to detect a -1.4 mg·kg/d decrease in LDL apoB100 PR for MK-0859 administered on a background of atorvastatin therapy compared to atorvastatin alone.

Assuming a pooled, within-subject standard deviation of 0.05 pools/d, N=30 subjects in Panel A, and a significance level of 0.05 (two-tailed), an increase of 0.026 pools/d in LDL apoB100 FCR can be detected with 80.0% probability for MK-0859 administered on a background of atorvastatin therapy compared to atorvastatin alone.

Secondary Hypothesis

Assuming a pooled, within-subject standard deviation of 0.05 pools/d, N=10 subjects in Panel B, and a significance level of 0.05 (two-tailed), there is 95.4% probability to detect a 0.065 pools/d increase in LDL apoB100 FCR for MK-0859 compared to placebo.

3.6 LABELING, PACKAGING, STORAGE, DISPENSING, AND RETURN OF CLINICAL SUPPLIES

3.6.1 Subject and Replacements Information

Clinical supplies will be packaged for 64 subjects to support enrollment and completion of approximately **40** subjects.

MK-0859 active/matching placebo and Atorvastatin (Lipitor™) active/matching placebo clinical supplies will be packaged according to an allocation schedule generated by the SPONSOR. CCI [redacted]

3.6.2 Product Descriptions

Investigational materials will be provided by the SPONSOR as summarized in Table 3-7.

Table 3-7

Product Descriptions

Product Name & Potency	Dosage Form	Comments
MK-0859 100 mg	tablet	N/A
Placebo to match MK-0859 100 mg	tablet	N/A
Atorvastatin (Lipitor™) 20 mg	tablet	N/A
Placebo to match Atorvastatin (Lipitor™) 20 mg	tablet	N/A
CCI [redacted]	[redacted]	[redacted]
[redacted]	[redacted]	[redacted]
[redacted]	[redacted]	[redacted]
[redacted]	[redacted]	[redacted]
[redacted]		

All placebos were created by Merck & Co., Inc in the image of the active product.

3.6.3 Primary Packaging and Labeling Information

The MK-0859 active/matching placebo and Atorvastatin (Lipitor™) active/matching placebo supplies will be packaged in HDPE bottles with child resistant closures; [redacted]

Table 3-8

Packaging of Clinical Supplies

Interval ID	Container ID	Product name & potency	Fill count	Dosing Instructions
Period 1	Bottle A	Placebo to match MK-0859 100 mg	40 tablets	Take 1 tablet daily in the evening with a meal
Period 1	Bottle B	Atorvastatin (Lipitor™) 20 mg or Placebo to match Atorvastatin (Lipitor™) 20 mg image	40 tablets	Take 1 tablet daily in the evening with a meal
Period 2	Bottle C	MK-0859 100 mg	67 tablets	Take 1 tablet daily in the evening with a meal
Period 2	Bottle D	Atorvastatin (Lipitor™) 20 mg or Placebo to match Atorvastatin (Lipitor™) 20 mg image	67 tablets	Take 1 tablet daily in the evening with a meal
[redacted]	[redacted]	[redacted]	[redacted]	[redacted]
[redacted]	[redacted]	[redacted]	[redacted]	[redacted]
[redacted]	[redacted]	[redacted]	[redacted]	[redacted]
[redacted]	[redacted]	[redacted]	[redacted]	[redacted]

MK-0859 Active/Matching Placebo and Atorvastatin (Lipitor™) Active/Matching Placebo Supplies

Container label text may include the following:

• Lot Trace ID #<br>• Allocation #<br>• Fill Count & Dosage Form<br>• Interval ID<br>• Container ID	• Dosing Instructions<br>• Storage Conditions<br>• Compound ID - Protocol #<br>• Country regulatory requirements<br>• SPONSOR address (If applicable)

[redacted]

[redacted]

3.6.4 Secondary Packaging and Labeling Information (kit)

MK-0859 active/matching placebo and Atorvastatin (Lipitor™) active/matching placebo supplies will be packaged in kit boxes as outlined in Table 3-9 below. Kit configuration is subject to change as a result of packaging constraints.

[redacted]

Table 3-9

Kit Contents

Interval Information	Kit Contents
Periods 1 and 2	4 Bottles (Bottles A, B, C, and D)

Label text may include the following:

• Lot Trace ID #<br>• Allocation #<br>• Kit Contents<br>• Interval ID	• Compound ID - Protocol #<br>• Country regulatory requirements<br>• SPONSOR address (If applicable)

3.6.5 Clinical Supplies Disclosure

MK-0859 active/matching placebo and Atorvastatin (Lipitor™) active/matching placebo supplies will be provided with blinded envelopes containing drug disclosure information. The SPONSOR will provide one sealed envelope to the investigator for each allocation number for each interval ID. [redacted]

Disclosure envelopes must be received by a designated person at the study site and kept in a secured location to which only the investigator and designated assistants have access. The envelope should be opened only in the case of an emergency if the drug identification information is necessary for the welfare of the patient. Every effort should be made not to unblind the patient unless necessary. Prior to unblinding, the investigator

will attempt to contact the CRA. Any unblinding that occurs at the site must be documented and the unblinded envelope retained at site. At the end of the study, all disclosure envelopes (sealed and unsealed) are to be returned to the SPONSOR.

Envelope information may include the following:

- Lot Trace ID or Barcode
- Allocation Number
- Interval ID
- Compound ID – Protocol #
- Drug identity (inside the envelope)

3.6.6 Storage and Handling Requirements

The storage conditions will be indicated on the product label.

The clinical supplies storage area at the site must be monitored by the site staff for temperature consistency with the acceptable storage temperature range specified in this protocol or in the product label attached to the protocol. Documentation of temperature monitoring should be maintained.

3.6.7 Standard Policies / Return of Clinical Supplies

Investigational clinical supplies must be received by a designated person at the study site, handled and stored safely and properly, and kept in a secured location to which only the investigator and designated assistants have access. Clinical supplies are to be dispensed only in accordance with the protocol. The investigator is responsible for keeping accurate records of the clinical supplies received from the SPONSOR, the amount dispensed to and returned by the patients, and the amount remaining at the conclusion of the study. In accordance with Good Pharmacy Practices, gloves should always be worn by study personnel if directly handling tablets or capsules that are returned (i.e., when counting returns). The Clinical Monitor should be contacted with any questions concerning investigational products where special or protective handling is indicated. At the end of the study, all clinical supplies including partial and empty containers must be returned as indicated on the Contact Information page(s).

U.S. sites should follow instructions for the Clinical Supplies Return Form (R464) and contact your SPONSOR representative for review of shipment and form before shipping.

3.6.8 Comparator Statement

At the close of the study after unblinding, a letter is to be sent by the investigator to those patients who received placebos in the image of the competitor's product to provide the following advice:

"You have participated in a study conducted by Merck & Co., Inc. This is to advise you that you were among those who received a look-alike tablet created by Merck & Co., Inc.

to resemble the drug Lipitor™ 20 mg (atorvastatin) as much as possible. You did not receive the active drug Lipitor™ 20 mg (atorvastatin) as manufactured by Pfizer."

3.7 DATA MANAGEMENT

Information regarding Data Management procedures for this protocol will be provided by the SPONSOR.

3.8 BIOLOGICAL SPECIMENS

Information regarding biological specimens for this protocol will be provided by the SPONSOR.

It is the responsibility of the primary investigator to ensure that all staff personnel who will be handling, packaging, and/or shipping clinical specimens act in conformance with International Air Transport Association (IATA) regulations to the handling and shipping of hazardous goods.

4. ADMINISTRATIVE AND REGULATORY DETAILS

4.1 CONFIDENTIALITY

4.1.1 Confidentiality of Data

For Studies Conducted Under the U.S. IND

Particular attention is drawn to the regulations promulgated by the Food and Drug Administration under the Freedom of Information Act providing, in part, that information furnished to clinical investigators and Institutional Review Boards will be kept confidential by the Food and Drug Administration only if maintained in confidence by the clinical investigator and Institutional Review Board.

For All Studies

By signing this protocol, the investigator affirms to the SPONSOR that information furnished to the investigator by the SPONSOR will be maintained in confidence and such information will be divulged to the Institutional Review Board, Ethics Review Committee, or similar or expert committee; affiliated institution; and employees only under an appropriate understanding of confidentiality with such board or committee, affiliated institution and employees. Data generated by this study will be considered confidential by the investigator, except to the extent that it is included in a publication as provided in the Publications section of this protocol.

4.1.2 Confidentiality of Subject/Patient Records

For All Studies

By signing this protocol, the investigator agrees that the SPONSOR (or SPONSOR representative), Institutional Review Board/Independent Ethics Committee (IRB/IEC), or Regulatory Agency representatives may consult and/or copy study documents in order to verify worksheet/case report form data. By signing the consent form, the subject/patient agrees to this process. If study documents will be photocopied during the process of verifying worksheet/case report form information, the subject/patient will be identified by unique code only; full names/initials will be masked prior to transmission to the SPONSOR.

For Studies Conducted Under the U.S. IND

By signing this protocol, the investigator agrees to treat all patient data used and disclosed in connection with this study in accordance with all applicable privacy laws, rules and regulations, including all applicable provisions of the Health Insurance Portability and Accountability Act and its implementing regulations, as amended from time to time. ("HIPAA").

4.1.3 Confidentiality of Investigator Information

For All Studies

By signing this protocol, the investigator recognizes that certain personal identifying information with respect to the investigator, and all subinvestigators and study site

personnel, may be used and disclosed for study management purposes, as part of a regulatory submissions, and as required by law. This information may include:

- name, address, telephone number, and email address;
- hospital or clinic address and telephone number;
- curriculum vitae or other summary of qualifications and credentials; and
- other professional documentation.

Consistent with the purposes described above, this information may be transmitted to the SPONSOR, and subsidiaries, affiliates and agents of the SPONSOR, in your country and other countries, including countries that do not have laws protecting such information. Additionally, the investigator's name and business contact information may be included when reporting certain serious adverse events to regulatory agencies or to other investigators. By signing this protocol, the investigator expressly consents to these uses and disclosures.

For Multicenter Studies
In order to facilitate contact between investigators, the SPONSOR may share an investigator's name and contact information with other participating investigators upon request.

4.2 COMPLIANCE WITH LAW, AUDIT, AND DEBARMENT

By signing this protocol, the investigator agrees to conduct the study in an efficient and diligent manner and in conformance with this protocol; generally accepted standards of Good Clinical Practice; and all applicable federal, state, and local laws, rules and regulations relating to the conduct of the clinical study.

The Code of Conduct, a collection of goals and considerations that govern the ethical and scientific conduct of clinical investigations sponsored by Merck & Co., Inc., is attached.

The investigator also agrees to allow monitoring, audits, Institutional Review Board/Independent Ethics Committee review, and regulatory agency inspection of trial-related documents and procedures and provide for direct access to all study-related source data and documents.

The investigator agrees not to seek reimbursement from subjects/patients, their insurance providers, or from government programs for procedures included as part of the study reimbursed to the investigator by the SPONSOR.

The Investigator shall prepare and maintain complete and accurate study documentation in compliance with Good Clinical Practice standards and applicable federal, state, and local laws, rules and regulations; and, for each subject/patient participating in the study, provide all data, and upon completion or termination of the clinical study submit any

other reports to the SPONSOR as required by this protocol or as otherwise required pursuant to any agreement with the SPONSOR.

Study documentation will be promptly and fully disclosed to the SPONSOR by the investigator upon request and also shall be made available at the investigator's site upon request for inspection, copying, review, and audit at reasonable times by representatives of the SPONSOR or any regulatory agencies. The investigator agrees to promptly take any reasonable steps that are requested by the SPONSOR as a result of an audit to cure deficiencies in the study documentation and worksheets/case report forms.

International Conference of Harmonization Good Clinical Practice guidelines (Section 4.3.3) recommend that the investigator inform the subject's primary physician about the subject's participation in the trial if the subject has a primary physician and if the subject agrees to the primary physician being informed.

According to European legislation, a SPONSOR must designate a principal or coordinating investigator (CI) to review the report (summarizing the study results) and confirm that to the best of his/her knowledge the report accurately describes conduct and results of the study. The SPONSOR may consider one or more factors in the selection of the individual to serve as the CI (e.g., thorough understanding of clinical trial methods, appropriate enrollment of subject/patient cohort, timely achievement of study milestones, availability of the CI during the anticipated review process).

The investigator will promptly inform the SPONSOR of any regulatory agency inspection conducted for this study.

Persons debarred from conducting or working on clinical studies by any court or regulatory agency will not be allowed to conduct or work on this SPONSOR's studies. The investigator will immediately disclose in writing to the SPONSOR if any person who is involved in conducting the study is debarred, or if any proceeding for debarment is pending or, to the best of the investigator's knowledge, threatened.

In the event the SPONSOR prematurely terminates a particular trial site, the SPONSOR will promptly notify that site's IRB/IEC.

4.3 COMPLIANCE WITH FINANCIAL DISCLOSURE REQUIREMENTS

By signing this protocol, the investigator agrees to provide to the SPONSOR accurate financial information to allow the SPONSOR to submit complete and accurate certification and disclosure statements as required by U.S. Food and Drug Administration regulations (21 CFR Part 54). The investigator further agrees to provide this information on a Financial Disclosure/Certification Form that is provided by Merck & Co., Inc. This requirement also extends to subinvestigators. The investigator also consents to the transmission of this information to Merck & Co., Inc. in the United States for these purposes. This may involve the transmission of information to countries that do not have laws protecting personal data.

4.4 QUALITY CONTROL AND QUALITY ASSURANCE

By signing this protocol, the SPONSOR agrees to be responsible for implementing and maintaining quality control and quality assurance systems with written SOPs to ensure that trials are conducted and data are generated, documented, and reported in compliance with the protocol, accepted standards of Good Clinical Practice, and all applicable federal, state, and local laws, rules and regulations relating to the conduct of the clinical study.

4.5 COMPLIANCE WITH INFORMATION PROGRAM ON CLINICAL TRIALS FOR SERIOUS OR LIFE THREATENING CONDITIONS

Under the terms of The Food and Drug Administration Modernization Act (FDAMA), the SPONSOR of the study is solely responsible for determining whether the study is subject to the requirements for submission to the Clinical Trials Data Bank, http://clinicaltrials.gov/. Merck, as SPONSOR of this study, will review this protocol and submit the information necessary to fulfill this requirement. Merck entries are not limited to FDAMA mandated trials. Merck’s voluntary listings, beyond those mandated by FDAMA, will be in the same format as for treatments for serious or life-threatening illnesses. Information posted will allow patients to identify potentially appropriate trials for their disease conditions and pursue participation by calling a central contact number for further information on appropriate study locations and site contact information.

By signing this protocol, the investigator acknowledges that the statutory obligation under FDAMA is that of the SPONSOR and agrees not to submit any information about this study to the Clinical Trials Data Bank.

4.6 PUBLICATIONS

As this study is part of a multicenter trial, publications derived from this study should include input from the investigator(s) and SPONSOR personnel. Such input should be reflected in publication authorship, and whenever possible, preliminary agreement regarding the strategy for order of authors’ names should be established before conducting the study. Subsequent to the multicenter publication, or 24 months after completion of the study, whichever comes first, an investigator and/or his/her colleagues may publish the results for their study site independently. However, the SPONSOR does not recommend separate publication of individual study site results due to scientific concerns.

The SPONSOR must have the opportunity to review all proposed abstracts, manuscripts, or presentations regarding this study 60 days prior to submission for publication/presentation. Any information identified by the SPONSOR as confidential must be deleted prior to submission. SPONSOR review can be expedited to meet publication guidelines.

5. LIST OF REFERENCES

1. Li J, Mehrotra D. An efficient method for accommodating potentially underpowered primary endpoints. Statistics in Medicine 2008;27:5377-5391.

2. Millar JS, Brousseau ME, Diffenderfer MR, Barret PHR, Welty FK, Farugi A, Wolfe ML, Nartsupha C, Digenio AG, Mancurso JP, Dolnikowski GG, Schaefer EJ, Rader DJ. Effects of the cholesteryl ester transfer protein inhibitor torcetrapib on apolipoprotein B100 metabolism in humans. Arterioscler Thromb Vasc Biol 2006; 26:1350-1356.

3. Bloomfield D, Carlson GL, Sapre A, Tribble D, McKenney JM, Littlejohn TW, Sisk CM, Mitchel Y, Pasternak RC. Efficacy and safety of the cholesteryl ester transfer protein inhibitor anacetrapib as monotherapy and coadministration with atorvastatin in dyslipidemic patients. Am Heart J 2009; 0:1-9.e2.

4. Brousseau ME, Diffenderfer MR, Millar JS, Nartsupha C, Asztalos BF, Welty FK, Wolfe ML, Rudling M, Bjorkhem I, Angelin B, Mancuso JP, Digenio AG, Rader DJ, Schaefer EJ. Effects of the cholesteryl ester transfer protein inhibition on high-density-lipoprotein subspecies, apolipoprotein A-I metabolism, and fecal sterol excretion. Arterioscler Thromb Vasc Biol 2005; 25:1057-1064.

5. Lamon-Fava S, Diffenderfer MR, Barrett PHR, Buchsbaum A, Matthan NR, Lichtenstein AH, Dolnikowski GG, Horvath K, Asztalos BF, Zago V, Schaefer EJ. Effects of different doses of atorvastatin on human apolipoprotein B-100, B-48, and A-1 metabolism. J Lipid Res 2007; 48:1546-1753.

6. CCI [redacted]

7. Mehrotra D, Heyse JF. Use of the false discovery rate for evaluating clinical safety data. Statistical Methods in Medical Research 2004; 13:227-238.

8. Benjamini Y, Hochberg Y. (1995) Controlling the false discovery rate: a practical and powerful approach to multiple testing. Journal of the Royal Statistical Society, Series B 1995; 57:289-300.

9. Paultre F, Pearson T, Weil HFC, Tuck CH, Myerson M, Rubin J, Francis C; Marx HF, Philbin EF, Reed RG, Berglund L. High levels of Lp(a) with a small apo(a) isoform are associated with coronary artery disease in african american and white men. Arterioscl Throm and Vas Biol 2000;20(12):2619-2624.

10. CCI [redacted]

11. CCI [redacted]

6. APPENDICES

6.1 CALCULATING BODY MASS INDEX (BMI)

BMI is calculated by taking the subject's weight, in kg, and dividing by the subject's height, in meters, squared (BMI = (Body weight in kilograms) ÷ (height in meters)^2.

BMI	**Height**(cm)																									
**Weight** kg.	**150**	**154**	**154**	**156**	**158**	**160**	**162**	**164**	**166**	**168**	**170**	**172**	**174**	**176**	**178**	**180**	**182**	**184**	**186**	**188**	**190**	**192**	**194**	**196**	**198**	**200**
60	27	26	25	24	24	23	23	22	22	21	21	20	20	19	19	18	18	17	17	17	16	16	16	15	15	15
62	28	27	26	26	25	24	24	23	22	22	21	21	20	20	19	19	19	18	18	17	17	16	16	16	16	15
64	28	28	27	26	26	25	24	24	23	22	22	22	21	21	20	19	19	19	18	18	17	17	17	16	16	16
66	29	29	28	27	26	26	25	24	24	23	23	22	22	21	21	20	20	19	19	18	18	17	17	17	17	16
68	30	30	29	28	27	26	26	25	25	24	23	23	22	22	21	21	20	20	19	19	19	18	18	17	17	17
70	31	30	30	29	28	27	27	26	25	25	24	24	23	23	22	21	21	20	20	20	19	18	18	18	18	17
72	32	31	30	30	29	28	27	27	26	25	25	24	24	23	23	22	22	21	20	20	20	19	19	18	18	18
74	33	32	31	30	30	29	28	27	27	26	26	25	24	24	23	23	22	22	21	21	20	20	20	19	19	19
76	34	33	32	31	30	30	29	28	27	27	26	26	25	24	24	23	23	22	22	21	21	20	20	20	19	19
78	35	34	33	32	31	30	30	29	28	27	27	26	26	25	24	24	23	23	22	22	21	21	21	20	20	19
80	36	35	34	33	32	31	30	30	29	28	27	27	26	26	25	24	24	23	23	22	22	22	21	21	20	20
82	36	36	35	34	33	32	31	30	30	29	28	27	27	26	26	25	25	24	23	23	22	22	22	21	21	20
84	37	37	35	35	34	33	32	31	30	30	29	28	28	27	26	26	25	25	24	23	23	22	22	22	21	21
86	38	37	36	35	34	34	33	32	31	30	30	29	28	28	27	26	26	25	25	24	23	23	23	22	22	21
88	39	38	37	36	35	34	33	32	31	30	30	29	28	28	27	26	26	25	25	24	23	23	23	22	22	21
90	40	39	38	37	36	35	34	33	33	32	31	30	30	29	28	28	27	26	26	25	25	24	24	23	23	22
92	41	40	39	38	37	36	35	34	33	33	32	31	30	30	29	28	28	27	26	26	25	25	24	24	23	23
94	42	41	40	39	38	37	36	35	34	33	32	32	31	30	30	29	28	28	27	26	26	25	25	24	24	23
96	43	42	41	40	38	38	37	36	35	34	33	32	32	31	30	30	29	28	28	27	26	26	25	25	24	24
98	44	43	41	40	39	38	37	36	35	35	34	33	32	32	31	30	30	29	28	28	27	26	26	25	25	24
100	44	43	42	41	40	39	38	37	36	35	35	34	33	32	32	31	30	29	29	28	28	27	26	26	25	25
102	45	44	43	42	41	40	39	38	37	36	35	34	34	33	32	31	31	30	29	29	28	28	27	26	26	25
104	46	45	44	43	42	41	40	39	38	37	36	35	34	34	33	32	31	31	30	29	29	28	28	27	26	26
106	47	46	45	44	42	41	41	39	38	38	37	36	35	34	33	33	32	31	31	30	29	29	28	28	27	26
108	48	47	46	44	43	42	41	40	39	38	37	36	36	35	34	33	33	32	31	31	30	29	29	28	28	27
110	49	48	46	45	44	43	42	41	40	39	38	37	36	35	35	34	33	32	32	31	31	30	29	29	28	28
110 +	49+	48+	46+	45	44	43	42	41	40	39	38	37	36	35	35	34	33	32	32	31	31	31	29	29	28	

6.2 LABORATORY SAFETY TESTS

**Hematology**		**Chemistry**	**Other Studies**
			PT/PTT (Prestudy only)
Hemoglobin		Sodium	Serum FSH ‡
Hematocrit		Potassium	Serum/urine β-hCG‡
WBC (total and differential)		Chloride	
Platelets		Bicarbonate	**Lipid Panel**
		Calcium	
**Urinalysis**		Creatinine	Total cholesterol
		Urea	HDL-C
Dipstick:	pH	Fasting blood sugar	Triglycerides
	Protein	Total Bilirubin†	LDL-C
	Glucose	AST (SGOT)	
Microscopic:	WBCs	ALT (SGPT)	
	RBCs	Alkaline phosphatase	
	Epithelial cells	Albumin	
	Casts (specify)		

† Fractionate if elevated.
‡ The information from FSH and pregnancy will not be collected in the database

Other Measurements

Plasma Lipoproteins

apoA-I
apoA-II
apoB100
apoC-II
apoC-III
apoE
Lp(a)

6.3 ALGORITHM FOR ASSESSING OUT-OF RANGE LABORATORY VALUES

For all laboratory values obtained at prestudy (screening) visit evaluation:

A. If all protocol-specified laboratory values are normal, the subject may enter the study.

B. If a protocol specified laboratory value is outside of the parameter(s) outlined in the inclusion/exclusion criteria (including a repeat if performed), the subject will be excluded from the study.

C. If ≥ 1 protocol-specified laboratory value not specified in the inclusion/exclusion criteria is outside the normal range, the following choices are available:

   1. The subject may be excluded from the study;

   2. The subject may be included in the study if the abnormal value(s) is not clinically significant (NCS) (the investigator must annotate the laboratory value "NCS" on the laboratory safety test source document);

   3. The subject be included in the study if the abnormality is consistent with a pre-existing medical condition which is not excluded per protocol (e.g., elevated eosinophil count in a subject with asthma or seasonal allergies) (this should be annotated on the laboratory report) or

   4. The abnormal test may be repeated (refer items a. and b. below for continuation of algorithm for repeated values).

      a. If the repeat test value is within the normal range, the subject may enter the study.

      b. If the repeat test value is still abnormal, the study investigator will evaluate the potential subject with a complete history and physical examination, looking especially for diseases that could result in the abnormal laboratory value in question. If such diseases can be ruled out, and if the abnormal laboratory value is not clinically relevant, then the subject may enter the study.

6.4 BLOOD DRAWN DURING STUDY

	Number of Collections	Volume/ Collection (mL)	Sampling Periods Per Study	Total Volume
Genomic analysis (if applicable)	1	10	1	10 mL
Laboratory tests (chemistry/hematology)				
FSH	1	5	1	5 mL
Serum β-hCG (prestudy and Poststudy)	1	5	2	10 mL
Prestudy, Visits 4, 7, 8, Poststudy	1	12	5	60 mL
Lipid Panel (Visits 2, 4 and 7)	1	2	3	6 mL
Serum lipoproteins (including lipid panel) LCAT and lipoprotein size by NMR (archive)	1	10	2	20 mL
Lipoprotein Kinetic Assays	18	22	2	792 mL
PCSK9 Concentration	1	3	2	6 mL
HL and LPL (Mass and Activity)	1	5	2	10 mL
Lathosterol	1	3	2	6 mL
MK-0859 (Archive)	1	4	12	48 mL
CETP concentration	1	3	2	6 mL
CETP activity	1	3	2	6 mL
Total Males (approximately)				975 mL
Total Females (approximately)				985 mL

6.5 ARCHIVE PLASMA FOR PCSK9 LEVELS—SAMPLE COLLECTION, HANDLING, LABELING, STORAGE, AND SHIPMENT

A. Plasma for PCSK9 Levels

1. Collection of Plasma

Samples for plasma PCSK9 levels will be obtained as indicated in the Flow Chart. See Flow Chart (Section 1.7) for blood collection times.

2. Sample Labeling

Specimens will be labeled with computer-generated labels which have been preprinted and color-coded for convenience. Labels can only be affixed to dry surfaces. They can be used on polyethylene, glass, etc. and will survive freezing and thawing. One label must be affixed directly to the tube containing the blood or plasma sample.

3. Procedures

a. Collect one 3-mL whole blood in a K2-EDTA (purple-top) tube. Immediately after collection, the blood-containing tubes should be inverted and placed on ice and centrifuged promptly at 3000 rpm (1500 g) at 4˚C for 10 minutes.

b. Plasma (at least 300 µL) should then be carefully transferred to 2 labeled 3.6-mL NUNC internal thread round bottom cryotubes (NUNC 366524).

c. Immediately after collection, plasma samples should be stored in a freezer at -70˚C until shipment for assay until transfer to Merck on DRY ICE.

d. In the event that the blood samples cannot be immediately processed, samples should be kept on ice. However, no more than 45 minutes should elapse between blood draw and freezing of samples.

B. Shipping of Samples

It is the responsibility of the primary investigator to ensure that all staff personnel who will be handling, packaging, and/or shipping clinical specimens act in conformance with International Air Transport Association (IATA) regulations relating to the handling and shipping of hazardous goods.

1. All shipments will be made in freezer boxes containing at least 20 kg DRY ICE and labeled as HUMAN SAMPLES: NONINFECTIOUS.

2. Please include a sample inventory with each shipment.

3. Shipments should be sent on MONDAY or TUESDAY (unless prior approval from SPONSOR to ship WEDNESDAY) to assure receipt by Friday.

4. PCSK9 samples should be shipped to:

   PPD
   Clinical Development Laboratories
   Merck Research Laboratories
   126 East Lincoln Avenue
   Building: RY50-137
   Rahway, NJ 07065
   Telephone: PPD
   FAX No.: PPD

6.6 ARCHIVE SERUM FOR CETP ACTIVITY—SAMPLE COLLECTION, HANDLING, LABELING, STORAGE, AND SHIPMENT

A. Serum for CETP Activity

1. Collection of Serum

Samples for serum CETP activity will be obtained as indicated in the Flow Chart. See Flow Chart (Section 1.7) for blood collection times.

2. Sample Labeling

Specimens will be labeled with computer-generated labels which have been preprinted and color-coded) for convenience. Labels can only be affixed to dry surfaces. They can be used on polyethylene, glass, etc. and will survive freezing and thawing. One label must be affixed directly to the tube containing the blood or plasma sample.

3. Procedures

a. At each collection, 3 mL of whole blood will be drawn into labeled VACUTAINER™ (red-top tube) **without neither anticoagulant nor serum separator**.

b. The blood-containing tubes should be allowed to clot at room temperature, for at least 10 minutes.

c. Blood samples will be centrifuged within 30 minutes of being drawn at 4˚C for 10 minutes at 3000 rpm and the serum withdrawn.

d. The serum will be aliquoted into 2 labeled, 3.6-mL Nunc cryotubes (NUNC 366524).

e. Serum samples should be placed in a freezer and stored at -70˚C until shipment to Merck Clinical Development Laboratory, Rahway, New Jersey for assay.

B. Shipping of Samples

It is the responsibility of the primary investigator to ensure that all staff personnel who will be handling, packaging, and/or shipping clinical specimens act in conformance with International Air Transport Association (IATA) regulations relating to the handling and shipping of hazardous goods.

1. All shipments will be made in freezer boxes containing at least 20 kg DRY ICE and labeled as HUMAN SAMPLES: NONINFECTIOUS.

2. Please include a sample inventory with each shipment.

3. Shipments should be sent on MONDAY or TUESDAY (unless prior approval from SPONSOR to ship WEDNESDAY) to assure receipt by Friday.

4. CETP samples should be shipped to:

PPD
Clinical Development Laboratories
Merck Research Laboratories
126 East Lincoln Avenue
Building: RY50-137
Rahway, NJ 07065
Telephone: PPD
FAX No.: PPD

6.7 ARCHIVE PLASMA FOR MK-0859—SAMPLE COLLECTION, HANDLING, LABELING, STORAGE, AND SHIPMENT

CCI [redacted]

[redacted]

[redacted]

[redacted]

[redacted]

[redacted]

[redacted]

[redacted]

[redacted]

[redacted]

[redacted]

[redacted]

[redacted]

CCI [redacted]

[redacted]

[redacted]

[redacted]

[redacted]

[redacted]

[redacted]

[redacted]

[redacted]

[redacted]

[redacted]

[redacted]

[redacted]

CCI [redacted]

[redacted]

6.8 CCI [redacted]

[redacted]

[redacted]

[redacted]

[redacted]

[redacted]

[redacted]

[redacted]

[redacted]

CCI [redacted]

[redacted] × [redacted] × [redacted]

6.9 EXPECTED EFFECTS FOR INTRA- AND INTER-PANEL COMPARISONS

		**Direction of Expected Effect**			
		**Intra-Panel Comparisons**		**Inter-Panel Comparisons**	
	**Parameter**	**MK-0859 w/ Atorvastatin – Atorvastatin (Panel A)**	**MK-0859 – Placebo (Panel B)**	**MK-0859 w/Atorvastatin – MK-0859 (Period 2)**	**Atorvastatin – Placebo (Period 1)**
Hypothesis Family 1 (F1) Endpoints	LDL apoB100 PR	Decrease	No Change	Decrease	No Change
	LDL apoB100 FCR	Increase	Increase	Increase	Increase
	LDL apoB100 PS	Decrease	Decrease	Decrease	Decrease
	IDL apoB100 PR	Decrease	Decrease	Decrease	Decrease
	IDL apoB100 FCR	Increase	Increase	Increase	Increase
	IDL apoB100 PS	Decrease	Decrease	Decrease	Decrease
	VLDL apoB100 PR	Increase	Increase / No Change	Increase	Increase
	VLDL apoB100 FCR	Increase	Increase	Increase	Increase
	VLDL apoB100 PS	Decrease	Decrease	Decrease	Decrease
	apoB100 Concentration	Decrease	Decrease	Decrease	Decrease
	apoC-II Concentration	No Change	No Change	Decrease	Decrease
	apoC-III Concentration	No Change	No Change	Decrease	Decrease
	apoE Concentration	Increase	Increase	Decrease	Decrease
	LPL Concentration	No Change	No Change	No Change	No Change
	HL Concentration	No Change	No Change	No Change	No Change
	LPL Activity	No Change	No Change	No Change	No Change
	HL Activity	No Change	No Change	No Change	No Change
	% conversion VLDL apoB to IDL apoB	Decrease	Decrease	No Change	No Change
	% conversion VLDL apoB to LDL apoB	Decrease	Decrease	No Change	No Change
	% conversion IDL apoB to LDL apoB	Decrease	Decrease	No Change	No Change
Hypothesis Family 2 (F2) Endpoints	HDL apoA-I PR	No Change	No Change	No Change	No Change
	HDL apoA-I FCR	Decrease	Decrease	No Change	No Change
	HDL apoA-I PS	Increase	Increase	No Change	No Change
	HDL apoA-II PR	No Change	No Change	No Change	No Change
	HDL apoA-II FCR	Decrease	Decrease	No Change	No Change
	HDL apoA-II PS	Increase	Increase	No Change	No Change
	apoA-I Concentration	Increase	Increase	No Change	No Change
	apoA-II Concentration	Increase	Increase	No Change	No Change
	LCAT Activity	Increase	Increase	No Change	No Change
Hypothesis Family 3 (F3) Endpoints	Lp(a) apo(a) PR	Decrease	Decrease	No Change	No Change
	Lp(a) apo(a) FCR	Increase	Increase	No Change	No Change
	Lp(a) apo(a) PS	Decrease	Decrease	No Change	No Change
	Lp(a) Concentration	Decrease	Decrease	No Change	No Change

6.10 PROCEDURE TO MEASURE FASTING LDL-C AND HDL-C (If TG are >400 mg/dL)

1. 1.5 mL plasma will be spun in an ultracentrifuge at density 1.006 g/ml which separates the VLDL fraction.

2. The top fraction will then be separated from the 1.006 g/ml bottom fraction, which contains LDL and HDL.

3. A cholesterol measurement will be obtained on the 1.006 g/ml bottom fraction.

4. HDL for analysis also prepared by adding 20 μL of precipitating reagent (1% dextran sulfate, 0.35 M MgCl2, 0.05% NaN3, final concentration) to 200 μL plasma. This precipitates VLDL and LDL.

5. HDL-cholesterol will than be measured in the supernatant fraction and corrected for the 10% dilution by the precipitating reagent.

Note: HDL-C may be determined according to site-specific SOPs.

Final Calculations:

HDL cholesterol = HDL supernatant cholesterol x 1.1 (to correct for dilution)

LDL cholesterol = 1.006 g/ml bottom cholesterol - HDL cholesterol

7. ATTACHMENTS

Lipitor® (Atorvastatin Calcium) Tablets

Merck & Co., Inc. Code of Conduct for Clinical Trials

Privacy Protection of Pharmacogenomic Sample Collections in Merck & Co., Inc. Clinical Trials: A Guideline for Clinicians and Privacy Board Members

Lipitor®
(Atorvastatin Calcium)
Tablets

DESCRIPTION

LIPITOR® (atorvastatin calcium) is a synthetic lipid-lowering agent. Atorvastatin is an inhibitor of 3-hydroxy-3-methylglutaryl-coenzyme A (HMG-CoA) reductase. This enzyme catalyzes the conversion of HMG-CoA to mevalonate, an early and rate-limiting step in cholesterol biosynthesis.

Atorvastatin calcium is [R-(R*, R*)]-2-(4-fluorophenyl)-ß, δ-dihydroxy-5-(1-methylethyl)-3-phenyl-4-[(phenylamino) carbonyl]-1H-pyrrole-1-heptanoic acid, calcium salt (2:1) trihydrate. The empirical formula of atorvastatin calcium is $(C_{33}H_{34}FN_2O_5)_2Ca \cdot 3H_2O$ and its molecular weight is 1209.42. Its structural formula is:

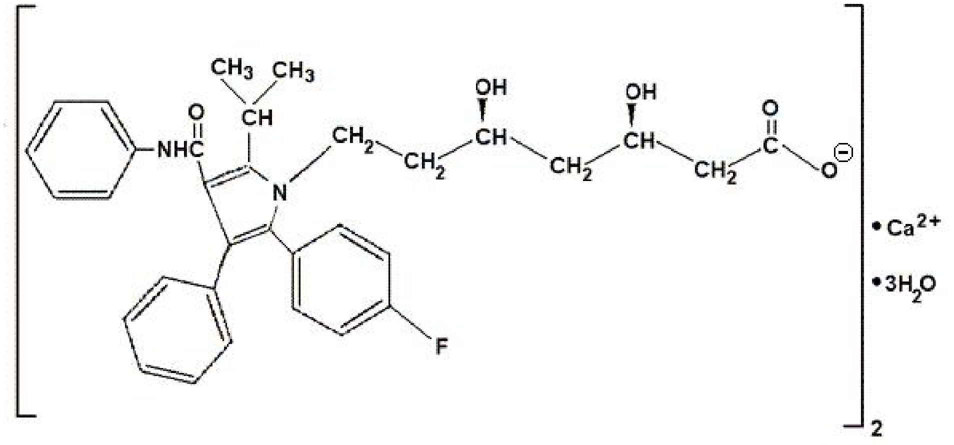

Atorvastatin calcium is a white to off-white crystalline powder that is insoluble in aqueous solutions of pH 4 and below. Atorvastatin calcium is very slightly soluble in distilled water, pH 7.4 phosphate buffer, and acetonitrile, slightly soluble in ethanol, and freely soluble in methanol.

LIPITOR tablets for oral administration contain 10, 20, 40 or 80 mg atorvastatin and the following inactive ingredients: calcium carbonate, USP; candelilla wax, FCC; croscarmellose sodium, NF; hydroxypropyl cellulose, NF; lactose monohydrate, NF; magnesium stearate, NF; microcrystalline cellulose, NF; Opadry White YS-1-7040 (hypromellose, polyethylene glycol, talc, titanium dioxide); polysorbate 80, NF; simethicone emulsion.

CLINICAL PHARMACOLOGY

Mechanism of Action

Atorvastatin is a selective, competitive inhibitor of HMG-CoA reductase, the rate-limiting enzyme that converts 3-hydroxy-3-methylglutaryl-coenzyme A to mevalonate, a precursor of sterols, including cholesterol. Cholesterol and triglycerides circulate in the bloodstream as part of lipoprotein complexes. With ultracentrifugation, these complexes separate into HDL (high-density lipoprotein), IDL (intermediate-density lipoprotein), LDL (low-density lipoprotein), and VLDL (very-low-density lipoprotein) fractions. Triglycerides (TG) and cholesterol in the liver are incorporated into VLDL and released into the plasma for delivery to peripheral tissues. LDL is formed from VLDL and is catabolized primarily through the high-affinity LDL receptor. Clinical and pathologic studies show that elevated plasma levels of total cholesterol (total-C), LDL-cholesterol (LDL-C), and apolipoprotein B (apo B) promote human atherosclerosis and are risk factors for developing cardiovascular disease, while increased levels of HDL-C are associated with a decreased cardiovascular risk.

In animal models, LIPITOR lowers plasma cholesterol and lipoprotein levels by inhibiting HMG-CoA reductase and cholesterol synthesis in the liver and by increasing the number of hepatic LDL receptors on the cell-surface to enhance uptake and catabolism of LDL; LIPITOR also reduces LDL production and the number of LDL particles. LIPITOR reduces LDL-C in some patients with homozygous familial hypercholesterolemia (FH), a population that rarely responds to other lipid-lowering medication(s).

A variety of clinical studies have demonstrated that elevated levels of total-C, LDL-C, and apo B (a membrane complex for LDL-C) promote human atherosclerosis. Similarly, decreased levels of HDL-C (and its transport complex, apo A) are associated with the development of atherosclerosis. Epidemiologic investigations have established that cardiovascular morbidity and mortality vary directly with the level of total-C and LDL-C, and inversely with the level of HDL-C.

LIPITOR reduces total-C, LDL-C, and apo B in patients with homozygous and heterozygous FH, nonfamilial forms of hypercholesterolemia, and mixed dyslipidemia. LIPITOR also reduces VLDL-C and TG and produces variable increases in HDL-C and apolipoprotein A-1. LIPITOR reduces total-C, LDL-C, VLDL-C, apo B, TG, and non-HDL-C, and increases HDL-C in patients with isolated hypertriglyceridemia. LIPITOR reduces intermediate density lipoprotein cholesterol (IDL-C) in patients with dysbetalipoproteinemia.

Like LDL, cholesterol-enriched triglyceride-rich lipoproteins, including VLDL, intermediate density lipoprotein (IDL), and remnants, can also promote atherosclerosis. Elevated plasma triglycerides are frequently found in a triad with low HDL-C levels and small LDL particles, as well as in association with non-lipid metabolic risk factors for

coronary heart disease. As such, total plasma TG has not consistently been shown to be an independent risk factor for CHD. Furthermore, the independent effect of raising HDL or lowering TG on the risk of coronary and cardiovascular morbidity and mortality has not been determined.

Pharmacodynamics

Atorvastatin as well as some of its metabolites are pharmacologically active in humans. The liver is the primary site of action and the principal site of cholesterol synthesis and LDL clearance. Drug dosage rather than systemic drug concentration correlates better with LDL-C reduction. Individualization of drug dosage should be based on therapeutic response (see DOSAGE AND ADMINISTRATION).

Pharmacokinetics and Drug Metabolism

Absorption: Atorvastatin is rapidly absorbed after oral administration; maximum plasma concentrations occur within 1 to 2 hours. Extent of absorption increases in proportion to atorvastatin dose. The absolute bioavailability of atorvastatin (parent drug) is approximately 14% and the systemic availability of HMG-CoA reductase inhibitory activity is approximately 30%. The low systemic availability is attributed to presystemic clearance in gastrointestinal mucosa and/or hepatic first-pass metabolism. Although food decreases the rate and extent of drug absorption by approximately 25% and 9%, respectively, as assessed by Cmax and AUC, LDL-C reduction is similar whether atorvastatin is given with or without food. Plasma atorvastatin concentrations are lower (approximately 30% for Cmax and AUC) following evening drug administration compared with morning. However, LDL-C reduction is the same regardless of the time of day of drug administration (see DOSAGE AND ADMINISTRATION).

Distribution: Mean volume of distribution of atorvastatin is approximately 381 liters. Atorvastatin is ≥98% bound to plasma proteins. A blood/plasma ratio of approximately 0.25 indicates poor drug penetration into red blood cells. Based on observations in rats, atorvastatin is likely to be secreted in human milk (see CONTRAINDICATIONS, Pregnancy and Lactation, and PRECAUTIONS, Nursing Mothers).

Metabolism: Atorvastatin is extensively metabolized to ortho- and parahydroxylated derivatives and various beta-oxidation products. *In vitro* inhibition of HMG-CoA reductase by ortho- and parahydroxylated metabolites is equivalent to that of atorvastatin. Approximately 70% of circulating inhibitory activity for HMG-CoA reductase is attributed to active metabolites. *In vitro* studies suggest the importance of atorvastatin metabolism by cytochrome P450 3A4, consistent with increased plasma concentrations of atorvastatin in humans following coadministration with erythromycin, a known inhibitor of this isozyme (see PRECAUTIONS, Drug Interactions). In animals, the ortho-hydroxy metabolite undergoes further glucuronidation.

Excretion: Atorvastatin and its metabolites are eliminated primarily in bile following hepatic and/or extra-hepatic metabolism; however, the drug does not appear to undergo

enterohepatic recirculation. Mean plasma elimination half-life of atorvastatin in humans is approximately 14 hours, but the half-life of inhibitory activity for HMG-CoA reductase is 20 to 30 hours due to the contribution of active metabolites. Less than 2% of a dose of atorvastatin is recovered in urine following oral administration.

Special Populations

Geriatric: Plasma concentrations of atorvastatin are higher (approximately 40% for Cmax and 30% for AUC) in healthy elderly subjects (age ≥65 years) than in young adults. Clinical data suggest a greater degree of LDL-lowering at any dose of drug in the elderly patient population compared to younger adults (see PRECAUTIONS section; Geriatric Use subsection).

Pediatric: Pharmacokinetic data in the pediatric population are not available.

Gender: Plasma concentrations of atorvastatin in women differ from those in men (approximately 20% higher for Cmax and 10% lower for AUC); however, there is no clinically significant difference in LDL-C reduction with LIPITOR between men and women.

Renal Insufficiency: Renal disease has no influence on the plasma concentrations or LDL-C reduction of atorvastatin; thus, dose adjustment in patients with renal dysfunction is not necessary (see DOSAGE AND ADMINISTRATION).

Hemodialysis: While studies have not been conducted in patients with end-stage renal disease, hemodialysis is not expected to significantly enhance clearance of atorvastatin since the drug is extensively bound to plasma proteins.

Hepatic Insufficiency: In patients with chronic alcoholic liver disease, plasma concentrations of atorvastatin are markedly increased. Cmax and AUC are each 4-fold greater in patients with Childs-Pugh A disease. Cmax and AUC are approximately 16-fold and 11-fold increased, respectively, in patients with Childs-Pugh B disease (see CONTRAINDICATIONS).

Clinical Studies

Prevention of Cardiovascular Disease

In the Anglo-Scandinavian Cardiac Outcomes Trial (ASCOT), the effect of LIPITOR (atorvastatin calcium) on fatal and non-fatal coronary heart disease was assessed in 10,305 hypertensive patients 40-80 years of age (mean of 63 years), without a previous myocardial infarction and with TC levels ≤251 mg/dl (6.5 mmol/l). Additionally all patients had at least 3 of the following cardiovascular risk factors: male gender (81.1%), age >55 years (84.5%), smoking (33.2%), diabetes (24.3%), history of CHD in a first-degree relative (26%), TC:HDL >6 (14.3%), peripheral vascular disease (5.1%), left ventricular hypertrophy (14.4%), prior cerebrovascular event (9.8%), specific ECG

abnormality (14.3%), proteinuria/albuminuria (62.4%). In this double-blind, placebo-controlled study patients were treated with anti-hypertensive therapy (Goal BP <140/90 mm Hg for non-diabetic patients, <130/80 mm Hg for diabetic patients) and allocated to either LIPITOR 10 mg daily (n=5168) or placebo (n=5137), using a covariate adaptive method which took into account the distribution of nine baseline characteristics of patients already enrolled and minimized the imbalance of those characteristics across the groups. Patients were followed for a median duration of 3.3 years.

The effect of 10 mg/day of LIPITOR on lipid levels was similar to that seen in previous clinical trials.

LIPITOR significantly reduced the rate of coronary events [either fatal coronary heart disease (46 events in the placebo group vs. 40 events in the LIPITOR group) or nonfatal MI (108 events in the placebo group vs. 60 events in the LIPITOR group)] with a relative risk reduction of 36% [(based on incidences of 1.9% for LIPITOR vs. 3.0% for placebo), p=0.0005 (see Figure 1)]. The risk reduction was consistent regardless of age, smoking status, obesity or presence of renal dysfunction. The effect of LIPITOR was seen regardless of baseline LDL levels. Due to the small number of events, results for women were inconclusive.

Figure 1: Effect of LIPITOR 10 mg/day on Cumulative Incidence of Nonfatal Myocardial Infarction or Coronary Heart Disease Death (in ASCOT-LLA)

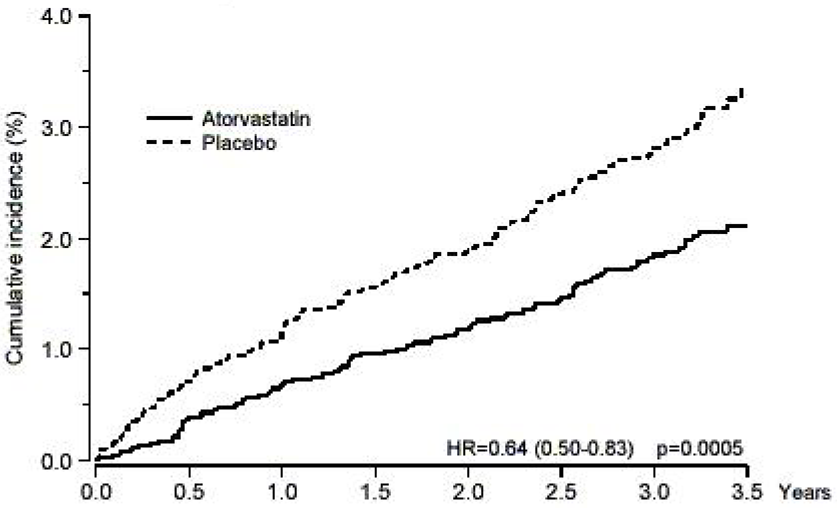

LIPITOR also significantly decreased the relative risk for revascularization procedures by 42%. Although the reduction of fatal and non-fatal strokes did not reach a pre-defined significance level (p=0.01), a favorable trend was observed with a 26% relative risk reduction (incidences of 1.7% for LIPITOR and 2.3% for placebo). There was no significant difference between the treatment groups for death due to cardiovascular causes (p=0.51) or noncardiovascular causes (p=0.17).

In the Collaborative Atorvastatin Diabetes Study (CARDS), the effect of LIPITOR (atorvastatin calcium) on cardiovascular disease (CVD) endpoints was assessed in 2838

subjects (94% White, 68% male), ages 40-75 with type 2 diabetes based on WHO criteria, without prior history of cardiovascular disease and with LDL≤ 160 mg/dL and TG ≤600 mg/dL. In addition to diabetes, subjects had 1 or more of the following risk factors: current smoking (23%), hypertension (80%), retinopathy (30%), or microalbuminuria (9%) or macroalbuminuria (3%). No subjects on hemodialysis were enrolled in the study. In this multicenter, placebo-controlled, double-blind clinical trial, subjects were randomly allocated to either LIPITOR 10 mg daily (1429) or placebo (1411) in a 1:1 ratio and were followed for a median duration of 3.9 years. The primary endpoint was the occurrence of any of the major cardiovascular events: myocardial infarction, acute CHD death, unstable angina, coronary revascularization, or stroke. The primary analysis was the time to first occurrence of the primary endpoint.

Baseline characteristics of subjects were: mean age of 62 years, mean HbA_{1c} 7.7%; median LDL-C 120 mg/dL; median TC 207 mg/dL; median TG 151 mg/dL; median HDL-C 52mg/dL.

The effect of LIPITOR 10 mg/ day on lipid levels was similar to that seen in previous clinical trials.

LIPITOR significantly reduced the rate of major cardiovascular events (primary endpoint events) (83 events in the LIPITOR group vs. 127 events in the placebo group) with a relative risk reduction of 37%, HR 0.63, 95% CI (0.48,0.83) (p=0.001) (see Figure 2). An effect of LIPITOR was seen regardless of age, sex, or baseline lipid levels.

Figure 2. Effect of LIPITOR 10 mg/day on Time to Occurrence of Major Cardiovascular Event (myocardial infarction, acute CHD death, unstable angina, coronary revascularization, or stroke) in CARDS.

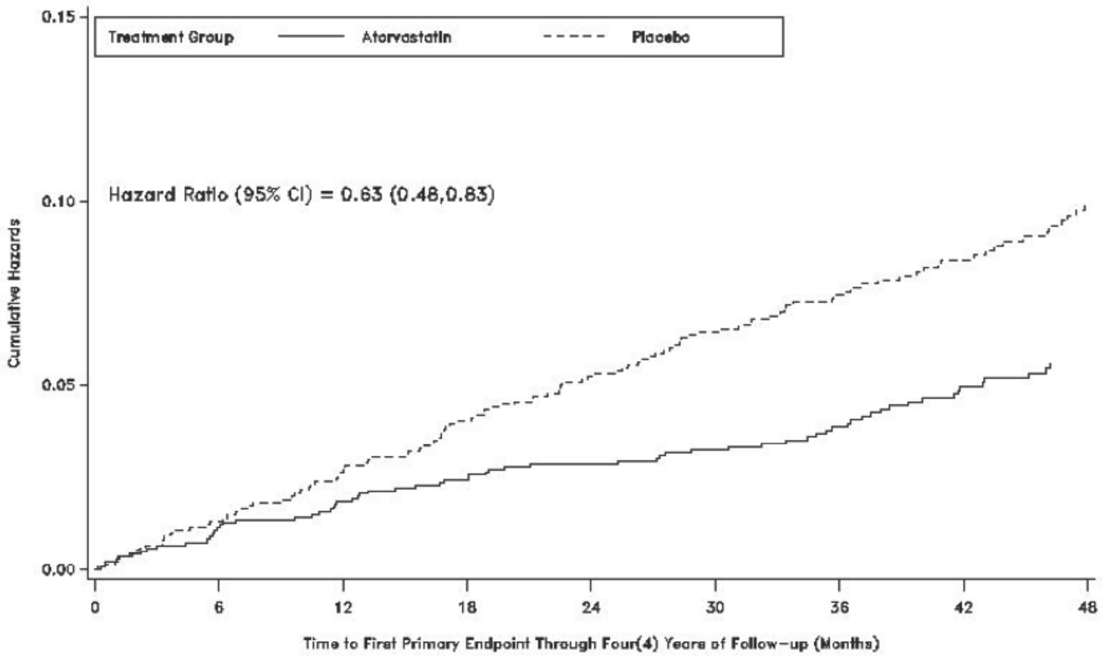

LIPITOR significantly reduced the risk of stroke by 48% (21 events in the LIPITOR group vs 39 events in the placebo group), HR 0.52, 95% CI (0.31,0.89) (p=0.016) and reduced the risk of MI by 42% (38 events in the LIPITOR group vs 64 events in the placebo group), HR 0.58, 95.1% CI (0.39, 0.86) (p=0.007). There was no significant difference between the treatment groups for angina, revascularization procedures, and acute CHD death.

There were 61 deaths in the LIPITOR group vs 82 deaths in the placebo group, (HR 0.73, p=0.059).

In the Treating to New Targets Study (TNT), the effect of LIPITOR 80 mg/day vs. LIPITOR 10 mg/day on the reduction in cardiovascular events was assessed in 10,001 subjects (94% white, 81% male, 38% ≥65 years) with clinically evident coronary heart disease who had achieved a target LDL-C level <130 mg/dL after completing an 8-week, open-label, run-in period with LIPITOR 10 mg/day. Subjects were randomly assigned to either 10 mg/day or 80 mg/day of LIPITOR and followed for a median duration of 4.9 years. The primary endpoint was the time-to-first occurrence of any of the following major cardiovascular events (MCVE): death due to CHD, non-fatal myocardial infarction, resuscitated cardiac arrest, and fatal and non-fatal stroke. The mean LDL-C,

TC, TG, non-HDL and HDL cholesterol levels at 12 weeks were 73, 145, 128, 98 and 47 mg/dL during treatment with 80 mg of LIPITOR and 99, 177, 152, 129 and 48 mg/dL during treatment with 10 mg of LIPITOR.

Treatment with LIPITOR 80 mg/day significantly reduced the rate of MCVE (434 events in the 80mg/day group vs 548 events in the 10 mg/day group) with a relative risk reduction of 22%, HR 0.78, 95% CI (0.69,0.89), p=0.0002 (see Figure 3 and Table 1). The overall risk reduction was consistent regardless of age (<65, ≥65) or gender.

Figure 3. Effect of LIPITOR 80 mg/day vs.10 mg/day on Time to Occurrence of Major Cardiovascular Events (TNT)

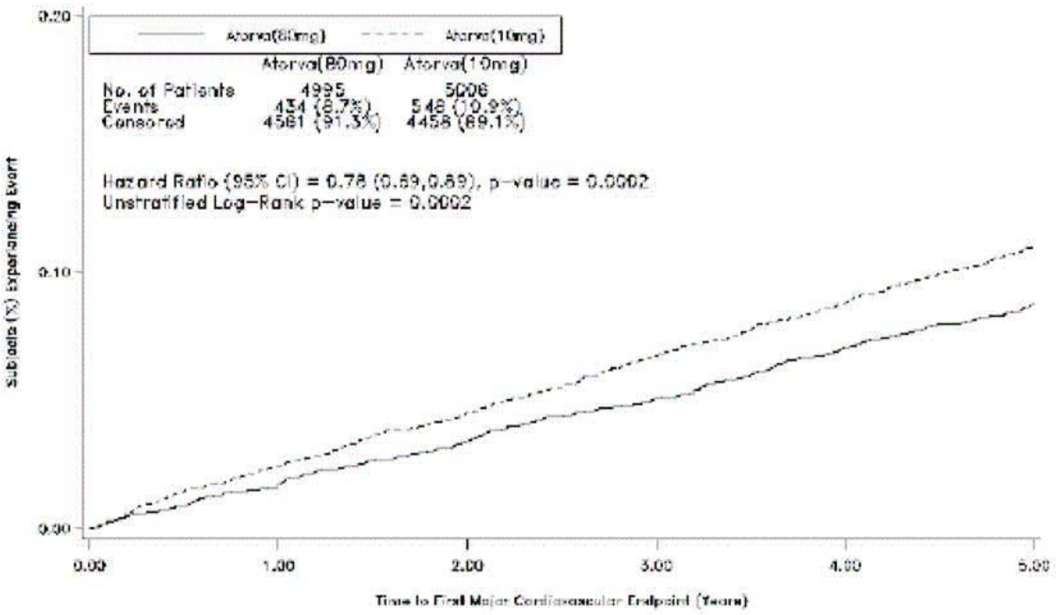

TABLE 1. Overview of Efficacy Results in TNT

Endpoint	Atorvastatin 10 mg (N=5006)		Atorvastatin 80 mg (N=4995)		HR[a] (95%CI)
**PRIMARY ENDPOINT**	n	(%)	n	(%)	
First major cardiovascular endpoint	548	(10.9)	434	(8.7)	0.78 (0.69, 0.89)
**Components of the Primary Endpoint**					
CHD death	127	(2.5)	101	(2.0)	0.80 (0.61, 1.03)
Nonfatal, non-procedure related MI	308	(6.2)	243	(4.9)	0.78 (0.66, 0.93)
Resuscitated cardiac arrest	26	(0.5)	25	(0.5)	0.96 (0.56, 1.67)
Stroke (fatal and non-fatal)	155	(3.1)	117	(2.3)	0.75 (0.59, 0.96)
**SECONDARY ENDPOINTS***					
First CHF with hospitalization	164	(3.3)	122	(2.4)	0.74 (0.59, 0.94)
First PVD endpoint	282	(5.6)	275	(5.5)	0.97 (0.83, 1.15)
First CABG or other coronary revascularization procedure[b]	904	(18.1)	667	(13.4)	0.72 (0.65, 0.80)
First documented angina endpoint[b]	615	(12.3)	545	(10.9)	0.88 (0.79, 0.99)
All cause mortality	282	(5.6)	284	(5.7)	1.01 (0.85, 1.19)
**Components of all cause mortality**					
Cardiovascular death	155	(3.1)	126	(2.5)	0.81 (0.64, 1.03)
Noncardiovascular death	127	(2.5)	158	(3.2)	1.25 (0.99, 1.57)
Cancer death	75	(1.5)	85	(1.7)	1.13 (0.83, 1.55)
Other non-CV death	43	(0.9)	58	(1.2)	1.35 (0.91, 2.00)
Suicide, homicide and other traumatic non-CV death	9	(0.2)	15	(0.3)	1.67 (0.73, 3.82)

a Atorvastatin 80 mg: atorvastatin 10 mg
b component of other secondary endpoints
* secondary endpoints not included in primary endpoint
HR=hazard ratio; CHD=coronary heart disease; CI=confidence interval; MI=myocardial infarction; CHF=congestive heart failure; CV=cardiovascular; PVD=peripheral vascular disease; CABG=coronary artery bypass graft
Confidence intervals for the Secondary Endpoints were not adjusted for multiple comparisons

Of the events that comprised the primary efficacy endpoint, treatment with LIPITOR 80 mg/day significantly reduced the rate of nonfatal, non-procedure related MI and fatal and non-fatal stroke, but not CHD death or resuscitated cardiac arrest (Table 1). Of the predefined secondary endpoints, treatment with LIPITOR 80 mg/day significantly reduced the rate of coronary revascularization, angina and hospitalization for heart failure, but not peripheral vascular disease. The reduction in the rate of CHF with hospitalization was only observed in the 8% of patients with a prior history of CHF.

There was no significant difference between the treatment groups for all-cause mortality (Table 1). The proportions of subjects who experienced cardiovascular death, including the components of CHD death and fatal stroke were numerically smaller in the LIPITOR 80 mg group than in the LIPITOR 10 mg treatment group. The proportions of subjects who experienced noncardiovascular death were numerically larger in the LIPITOR 80 mg group than in the LIPITOR 10 mg treatment group.

In the Incremental Decrease in Endpoints Through Aggressive Lipid Lowering Study (IDEAL), treatment with LIPITOR 80 mg/day was compared to treatment with simvastatin 20-40 mg/day in 8,888 subjects up to 80 years of age with a history of CHD to assess whether reduction in CV risk could be achieved. Patients were mainly male (81%), white (99%) with an average age of 61.7 years, and an average

LDL-C of 121.5 mg/dL at randomization; 76% were on statin therapy. In this prospective, randomized, open-label, blinded endpoint (PROBE) trial with no run-in period, subjects were followed for a median duration of 4.8 years. The mean LDL-C, TC, TG, HDL and non-HDL cholesterol levels at Week 12 were 78, 145, 115, 45 and 100 mg/dL during treatment with 80 mg of LIPITOR and 105, 179, 142, 47 and 132 mg/dL during treatment with 20-40 mg of simvastatin.

There was no significant difference between the treatment groups for the primary endpoint, the rate of first major coronary event (fatal CHD, nonfatal MI and resuscitated cardiac arrest): 411 (9.3%) in the LIPITOR 80 mg/day group vs. 463 (10.4%) in the simvastatin 20-40 mg/day group, HR 0.89, 95% CI (0.78,1.01), p=0.07.

There were no significant differences between the treatment groups for all-cause mortality: 366 (8.2%) in the LIPITOR 80 mg/day group vs. 374 (8.4%) in the simvastatin 20-40 mg/day group. The proportions of subjects who experienced CV or non-CV death were similar for the LIPITOR 80 mg group and the simvastatin 20-40 mg group.

Hypercholesterolemia (Heterozygous Familial and Nonfamilial) and Mixed Dyslipidemia *(Fredrickson* Types IIa and IIb)

LIPITOR reduces total-C, LDL-C, VLDL-C, apo B, and TG, and increases HDL-C in patients with hypercholesterolemia and mixed dyslipidemia. Therapeutic response is seen within 2 weeks, and maximum response is usually achieved within 4 weeks and maintained during chronic therapy.

LIPITOR is effective in a wide variety of patient populations with hypercholesterolemia, with and without hypertriglyceridemia, in men and women, and in the elderly. Experience in pediatric patients has been limited to patients with homozygous FH. In two multicenter, placebo-controlled, dose-response studies in patients with hypercholesterolemia, LIPITOR given as a single dose over 6 weeks significantly reduced total-C, LDL-C, apo B, and TG (Pooled results are provided in Table 2).

TABLE 2. Dose-Response in Patients With Primary Hypercholesterolemia (Adjusted Mean % Change From Baseline)[a]

Dose	N	TC	LDL-C	Apo B	TG	HDL-C	Non-HDL-C/ HDL-C
Placebo	21	4	4	3	10	-3	7
10	22	-29	-39	-32	-19	6	-34
20	20	-33	-43	-35	-26	9	-41
40	21	-37	-50	-42	-29	6	-45
80	23	-45	-60	-50	-37	5	-53

[a] Results are pooled from 2 dose-response studies.

In patients with *Fredrickson* Types IIa and IIb hyperlipoproteinemia pooled from 24 controlled trials, the median (25th and 75th percentile) percent changes from baseline in HDL-C for atorvastatin 10, 20, 40, and 80 mg were 6.4 (-1.4, 14), 8.7(0, 17), 7.8(0, 16),

and 5.1 (-2.7, 15), respectively. Additionally, analysis of the pooled data demonstrated consistent and significant decreases in total-C, LDL-C, TG, total-C/HDL-C, and LDL-C/HDL-C.

In three multicenter, double-blind studies in patients with hypercholesterolemia, LIPITOR was compared to other HMG-CoA reductase inhibitors. After randomization, patients were treated for 16 weeks with either LIPITOR 10 mg per day or a fixed dose of the comparative agent (Table 3).

TABLE 3. Mean Percent Change From Baseline at Endpoint (Double-Blind, Randomized, Active-Controlled Trials)

Treatment (Daily Dose)	N	Total-C	LDL-C	Apo B	TG	HDL-C	Non-HDL-C/ HDL-C
Study 1							
Atorvastatin 10 mg	707	-27[a]	-36[a]	-28[a]	-17[a]	+7	-37[a]
Lovastatin 20 mg	191	-19	-27	-20	- 6	+7	-28
95% CI for Diff[1]		-9.2, -6.5	-10.7, -7.1	-10.0, -6.5	-15.2, -7.1	-1.7, 2.0	-11.1, -7.1
Study 2							
Atorvastatin 10 mg	222	-25[b]	-35[b]	-27[b]	-17[b]	+6	-36[b]
Pravastatin 20 mg	77	-17	-23	-17	- 9	+8	-28
95% CI for Diff[1]		-10.8, -6.1	-14.5, -8.2	-13.4, -7.4	-14.1, -0.7	-4.9, 1.6	-11.5, -4.1
Study 3							
Atorvastatin 10 mg	132	-29[c]	-37[c]	-34[c]	-23[c]	+7	-39[c]
Simvastatin 10 mg	45	-24	-30	-30	-15	+7	-33
95% CI for Diff[1]		-8.7, -2.7	-10.1, -2.6	-8.0, -1.1	-15.1, -0.7	-4.3, 3.9	-9.6, -1.9

[1] A negative value for the 95% CI for the difference between treatments favors atorvastatin for all except HDL-C, for which a positive value favors atorvastatin. If the range does not include 0, this indicates a statistically significant difference.

[a] Significantly different from lovastatin, ANCOVA, $p \leq 0.05$

[b] Significantly different from pravastatin, ANCOVA, $p \leq 0.05$

[c] Significantly different from simvastatin, ANCOVA, $p \leq 0.05$

The impact on clinical outcomes of the differences in lipid-altering effects between treatments shown in Table 3 is not known. Table 3 does not contain data comparing the effects of atorvastatin 10 mg and higher doses of lovastatin, pravastatin, and simvastatin. The drugs compared in the studies summarized in the table are not necessarily interchangeable.

Hypertriglyceridemia *(Fredrickson* Type IV)

The response to LIPITOR in 64 patients with isolated hypertriglyceridemia treated across several clinical trials is shown in the table below. For the atorvastatin-treated patients, median (min, max) baseline TG level was 565 (267-1502).

TABLE 4. Combined Patients With Isolated Elevated TG: Median (min, max) Percent Changes From Baseline

	Placebo (N=12)	Atorvastatin 10 mg (N=37)	Atorvastatin 20 mg (N=13)	Atorvastatin 80 mg (N=14)
Triglycerides	-12.4 (-36.6, 82.7)	-41.0 (-76.2, 49.4)	-38.7 (-62.7, 29.5)	-51.8 (-82.8, 41.3)
Total-C	-2.3 (-15.5, 24.4)	-28.2 (-44.9, -6.8)	-34.9 (-49.6, -15.2)	-44.4 (-63.5, -3.8)
LDL-C	3.6 (-31.3, 31.6)	-26.5 (-57.7, 9.8)	-30.4 (-53.9, 0.3)	-40.5 (-60.6, -13.8)
HDL-C	3.8 (-18.6, 13.4)	13.8 (-9.7, 61.5)	11.0 (-3.2, 25.2)	7.5 (-10.8, 37.2)
VLDL-C	-1.0 (-31.9, 53.2)	-48.8 (-85.8, 57.3)	-44.6 (-62.2, -10.8)	-62.0 (-88.2, 37.6)
non-HDL-C	-2.8 (-17.6, 30.0)	-33.0 (-52.1, -13.3)	-42.7 (-53.7, -17.4)	-51.5 (-72.9, -4.3)

Dysbetalipoproteinemia *(Fredrickson* Type III)

The results of an open-label crossover study of 16 patients (genotypes: 14 apo E2/E2 and 2 apo E3/E2) with dysbetalipoproteinemia *(Fredrickson* Type III) are shown in the table below.

TABLE 5. Open-Label Crossover Study of 16 Patients With Dysbetalipoproteinemia (*Fredrickson* Type III)

		Median % Change (min, max)	
	Median (min, max) at Baseline (mg/dL)	Atorvastatin 10 mg	Atorvastatin 80 mg
Total-C	442 (225, 1320)	-37 (-85, 17)	-58 (-90, -31)
Triglycerides	678 (273, 5990)	-39 (-92, -8)	-53 (-95, -30)
IDL-C + VLDL-C	215 (111, 613)	-32 (-76, 9)	-63 (-90, -8)
non-HDL-C	411 (218, 1272)	-43 (-87, -19)	-64 (-92, -36)

Homozygous Familial Hypercholesterolemia

In a study without a concurrent control group, 29 patients ages 6 to 37 years with homozygous FH received maximum daily doses of 20 to 80 mg of LIPITOR. The mean LDL-C reduction in this study was 18%. Twenty-five patients with a reduction in LDL-C had a mean response of 20% (range of 7% to 53%, median of 24%); the remaining 4 patients had 7% to 24% increases in LDL-C. Five of the 29 patients had absent LDL-receptor function. Of these, 2 patients also had a portacaval shunt and had no significant reduction in LDL-C. The remaining 3 receptor-negative patients had a mean LDL-C reduction of 22%.

Heterozygous Familial Hypercholesterolemia in Pediatric Patients
In a double-blind, placebo-controlled study followed by an open-label phase, 187 boys and postmenarchal girls 10-17 years of age (mean age 14.1 years) with heterozygous familial hypercholesterolemia (FH) or severe hypercholesterolemia were randomized to LIPITOR (n=140) or placebo (n=47) for 26 weeks and then all received LIPITOR for 26 weeks. Inclusion in the study required 1) a baseline LDL-C level ≥ 190 mg/dL or 2) a

baseline LDL-C ≥ 160 mg/dL and positive family history of FH or documented premature cardiovascular disease in a first- or second-degree relative. The mean baseline LDL-C value was 218.6 mg/dL (range: 138.5-385.0 mg/dL) in the LIPITOR group compared to 230.0 mg/dL (range: 160.0-324.5 mg/dL) in the placebo group. The dosage of LIPITOR (once daily) was 10 mg for the first 4 weeks and up-titrated to 20 mg if the LDL-C level was > 130 mg/dL. The number of LIPITOR-treated patients who required up-titration to 20 mg after Week 4 during the double-blind phase was 80 (57.1%).

LIPITOR significantly decreased plasma levels of total-C, LDL-C, triglycerides, and apolipoprotein B during the 26 week double-blind phase (see Table 6).

TABLE 6
Lipid-altering Effects of LIPITOR in Adolescent Boys and Girls with Heterozygous Familial Hypercholesterolemia or Severe Hypercholesterolemia
(Mean Percent Change from Baseline at Endpoint in Intention-to-Treat Population)

DOSAGE	N	Total-C	LDL-C	HDL-C	TG	Apolipoprotein B
Placebo	47	-1.5	-0.4	-1.9	1.0	0.7
LIPITOR	140	-31.4	-39.6	2.8	-12.0	-34.0

The mean achieved LDL-C value was 130.7 mg/dL (range: 70.0-242.0 mg/dL) in the LIPITOR group compared to 228.5 mg/dL (range: 152.0-385.0 mg/dL) in the placebo group during the 26 week double-blind phase.

The safety and efficacy of doses above 20 mg have not been studied in controlled trials in children. The long-term efficacy of LIPITOR therapy in childhood to reduce morbidity and mortality in adulthood has not been established.

INDICATIONS AND USAGE

Prevention of Cardiovascular Disease

In adult patients without clinically evident coronary heart disease, but with multiple risk factors for coronary heart disease such as age, smoking, hypertension, low HDL-C, or a family history of early coronary heart disease, LIPITOR is indicated to:

- Reduce the risk of myocardial infarction
- Reduce the risk of stroke
- Reduce the risk for revascularization procedures and angina

In patients with type 2 diabetes, and without clinically evident coronary heart disease, but with multiple risk factors for coronary heart disease such as retinopathy, albuminuria, smoking, or hypertension, LIPITOR is indicated to:

- Reduce the risk of myocardial infarction
- Reduce the risk of stroke

In patients with clinically evident coronary heart disease, LIPITOR is indicated to:

- Reduce the risk of non-fatal myocardial infarction
- Reduce the risk of fatal and non-fatal stroke

- Reduce the risk for revascularization procedures
- Reduce the risk of hospitalization for CHF
- Reduce the risk of angina

Hypercholesterolemia
LIPITOR is indicated:

1. as an adjunct to diet to reduce elevated total-C, LDL-C, apo B, and TG levels and to increase HDL-C in patients with primary hypercholesterolemia (heterozygous familial and nonfamilial) and mixed dyslipidemia *(Fredrickson* Types IIa and IIb);

2. as an adjunct to diet for the treatment of patients with elevated serum TG levels*(Fredrickson* Type IV);

3. for the treatment of patients with primary dysbetalipoproteinemia *(Fredrickson* Type III) who do not respond adequately to diet;

4. to reduce total-C and LDL-C in patients with homozygous familial hypercholesterolemia as an adjunct to other lipid-lowering treatments (eg, LDL apheresis) or if such treatments are unavailable;

5. as an adjunct to diet to reduce total-C, LDL-C, and apo B levels in boys and postmenarchal girls, 10 to 17 years of age, with heterozygous familial hypercholesterolemia if after an adequate trial of diet therapy the following findings are present:

   a. LDL-C remains ≥ 190 mg/dL or
   b. LDL-C remains ≥ 160 mg/dL and:
      - there is a positive family history of premature cardiovascular disease or
      - two or more other CVD risk factors are present in the pediatric patient

Therapy with lipid-altering agents should be a component of multiple-risk-factor intervention in individuals at increased risk for atherosclerotic vascular disease due to hypercholesterolemia. Lipid-altering agents should be used in addition to a diet restricted in saturated fat and cholesterol only when the response to diet and other nonpharmacological measures has been inadequate (see *National Cholesterol Education Program (NCEP) Guidelines*, summarized in Table 7).

TABLE 7. NCEP Treatment Guidelines: LDL-C Goals and Cutpoints for Therapeutic Lifestyle Changes and Drug Therapy in Different Risk Categories

Risk Category	LDL-C Goal (mg/dL)	LDL Level at Which to Initiate Therapeutic Lifestyle Changes (mg/dL)	LDL Level at Which to Consider Drug Therapy (mg/dL)
CHD [a] or CHD risk equivalents (10-year risk >20%)	<100	≥100	≥130 (100-129: drug optional) [b]

2+ Risk Factors (10-year risk ≤20%)	<130	≥130	10-year risk 10%-20%: ≥130<br>10-year risk <10%: ≥ 160
0-1 Risk factor [c]	<160	≥160	≥190<br>(160-189: LDL-lowering drug optional)

[a] CHD, coronary heart disease
[b] Some authorities recommend use of LDL-lowering drugs in this category if an LDL-C level of < 100 mg/dL cannot be achieved by therapeutic lifestyle changes. Others prefer use of drugs that primarily modify triglycerides and HDL-C, e.g., nicotinic acid or fibrate. Clinical judgement also may call for deferring drug therapy in this subcategory.
[c] Almost all people with 0-1 risk factor have 10-year risk <10%; thus, 10-year risk assessment in people with 0-1 risk factor is not necessary.

After the LDL-C goal has been achieved, if the TG is still ≥200 mg/dL, non-HDL-C (total-C minus HDL-C) becomes a secondary target of therapy. Non-HDL-C goals are set 30 mg/dL higher than LDL-C goals for each risk category.

Prior to initiating therapy with LIPITOR, secondary causes for hypercholesterolemia (e.g., poorly controlled diabetes mellitus, hypothyroidism, nephrotic syndrome, dysproteinemias, obstructive liver disease, other drug therapy, and alcoholism) should be excluded, and a lipid profile performed to measure total-C, LDL-C, HDL-C, and TG. For patients with TG <400 mg/dL (<4.5 mmol/L), LDL-C can be estimated using the following equation: LDL-C = total-C - (0.20 x [TG] + HDL-C). For TG levels >400 mg/dL (>4.5 mmol/L), this equation is less accurate and LDL-C concentrations should be determined by ultracentrifugation.

LIPITOR has not been studied in conditions where the major lipoprotein abnormality is elevation of chylomicrons (*Fredrickson* Types I and V).

The NCEP classification of cholesterol levels in pediatric patients with a familial history of hypercholesterolemia or premature cardiovascular disease is summarized below:

Category	Total-C (mg/dL)	LDL-C (mg/dL)
Acceptable	<170	<110
Borderline	170-199	110-129
High	≥200	≥130

CONTRAINDICATIONS

Active liver disease or unexplained persistent elevations of serum transaminases.

Hypersensitivity to any component of this medication.

Pregnancy and Lactation

Atherosclerosis is a chronic process and discontinuation of lipid-lowering drugs during pregnancy should have little impact on the outcome of long-term therapy of primary hypercholesterolemia. Cholesterol and other products of cholesterol biosynthesis are essential components for fetal development (including synthesis of steroids and cell membranes). Since HMG-CoA reductase inhibitors decrease cholesterol synthesis and possibly the synthesis of other biologically active substances derived from cholesterol, they may cause fetal harm when administered to pregnant women. Therefore, HMG-CoA reductase inhibitors are contraindicated during pregnancy and in nursing mothers. ATORVASTATIN SHOULD BE ADMINISTERED TO WOMEN OF CHILDBEARING AGE ONLY WHEN SUCH PATIENTS ARE HIGHLY UNLIKELY TO CONCEIVE AND HAVE BEEN INFORMED OF THE POTENTIAL HAZARDS. If the patient becomes pregnant while taking this drug, therapy should be discontinued and the patient apprised of the potential hazard to the fetus.

WARNINGS

Liver Dysfunction

HMG-CoA reductase inhibitors, like some other lipid-lowering therapies, have been associated with biochemical abnormalities of liver function. **Persistent elevations (>3 times the upper limit of normal [ULN] occurring on 2 or more occasions) in serum transaminases occurred in 0.7% of patients who received atorvastatin in clinical trials. The incidence of these abnormalities was 0.2%, 0.2%, 0.6%, and 2.3% for 10, 20, 40, and 80 mg, respectively.**

One patient in clinical trials developed jaundice. Increases in liver function tests (LFT) in other patients were not associated with jaundice or other clinical signs or symptoms. Upon dose reduction, drug interruption, or discontinuation, transaminase levels returned to or near pretreatment levels without sequelae. Eighteen of 30 patients with persistent LFT elevations continued treatment with a reduced dose of atorvastatin.

It is recommended that liver function tests be performed prior to and at 12 weeks following both the initiation of therapy and any elevation of dose, and periodically (e.g., semiannually) thereafter. Liver enzyme changes generally occur in the first 3 months of treatment with atorvastatin. Patients who develop increased transaminase levels should be monitored until the abnormalities resolve. Should an increase in ALT or AST of >3 times ULN persist, reduction of dose or withdrawal of atorvastatin is recommended.

Atorvastatin should be used with caution in patients who consume substantial quantities of alcohol and/or have a history of liver disease. Active liver disease or unexplained persistent transaminase elevations are contraindications to the use of atorvastatin (see CONTRAINDICATIONS).

Skeletal Muscle

Rare cases of rhabdomyolysis with acute renal failure secondary to myoglobinuria have been reported with atorvastatin and with other drugs in this class.

Uncomplicated myalgia has been reported in atorvastatin-treated patients (see ADVERSE REACTIONS). Myopathy, defined as muscle aches or muscle weakness in conjunction with increases in creatine phosphokinase (CPK) values >10 times ULN, should be considered in any patient with diffuse myalgias, muscle tenderness or weakness, and/or marked elevation of CPK. Patients should be advised to report promptly unexplained muscle pain, tenderness or weakness, particularly if accompanied by malaise or fever. Atorvastatin therapy should be discontinued if markedly elevated CPK levels occur or myopathy is diagnosed or suspected.

The risk of myopathy during treatment with drugs in this class is increased with concurrent administration of cyclosporine, fibric acid derivatives, erythromycin, clarithromycin, combination of ritonavir plus saquinavir or lopinavir plus ritonavir, niacin, or azole antifungals. Physicians considering combined therapy with atorvastatin and fibric acid derivatives, erythromycin, clarithromycin, a combination of ritonavir plus saquinavir or lopinavir plus ritonavir, immunosuppressive drugs, azole antifungals, or lipid-modifying doses of niacin should carefully weigh the potential benefits and risks and should carefully monitor patients for any signs or symptoms of muscle pain, tenderness, or weakness, particularly during the initial months of therapy and during any periods of upward dosage titration of either drug. Lower starting and maintenance doses of atorvastatin should be considered when taken concomitantly with the aforementioned drugs (See DRUG INTERACTIONS). Periodic creatine phosphokinase (CPK) determinations may be considered in such situations, but there is no assurance that such monitoring will prevent the occurrence of severe myopathy.

Atorvastatin therapy should be temporarily withheld or discontinued in any patient with an acute, serious condition suggestive of a myopathy or having a risk factor predisposing to the development of renal failure secondary to rhabdomyolysis (e.g., severe acute infection, hypotension, major surgery, trauma, severe metabolic, endocrine and electrolyte disorders, and uncontrolled seizures).

PRECAUTIONS

General

Before instituting therapy with atorvastatin, an attempt should be made to control hypercholesterolemia with appropriate diet, exercise, and weight reduction in obese patients, and to treat other underlying medical problems (see INDICATIONS AND USAGE).

Information for Patients

Patients should be advised to report promptly unexplained muscle pain, tenderness, or weakness, particularly if accompanied by malaise or fever.

Drug Interactions

The risk of myopathy during treatment with HMG-CoA reductase inhibitors is increased with concurrent administration of fibric acid derivatives, lipid-modifying doses of niacin or cytochrome P450 3A4 inhibitors (e.g. cyclosporine, erythromycin, clarithromycin, and azole antifungals) (see WARNINGS, Skeletal Muscle).

Inhibitors of cytochrome P450 3A4: Atorvastatin is metabolized by cytochrome P450 3A4. Concomitant administration of atorvastatin with inhibitors of cytochrome P450 3A4 can lead to increases in plasma concentrations of atorvastatin. The extent of interaction and potentiation of effects depends on the variability of effect on cytochrome P450 3A4.

Clarithromycin: Concomitant administration of atorvastatin 80 mg with clarithromycin (500 mg twice daily) resulted in a 4.4-fold increase in atorvastatin AUC (see WARNINGS, Skeletal Muscle, and DOSAGE AND ADMINISTRATION).

Erythromycin: In healthy individuals, plasma concentrations of atorvastatin increased approximately 40% with co-administration of atorvastatin and erythromycin, a known inhibitor of cytochrome P450 3A4 (see WARNINGS, Skeletal Muscle).

Combination of Protease Inhibitors: Concomitant administration of atorvastatin 40 mg with ritonavir plus saquinavir (400 mg twice daily) resulted in a 3-fold increase in atorvastatin AUC. Concomitant administration of atorvastatin 20 mg with lopinavir plus ritonavir (400 mg+100 mg twice daily) resulted in a 5.9-fold increase in atorvastatin AUC (see WARNINGS, Skeletal Muscle, and DOSAGE AND ADMINISTRATION).

Itraconazole: Concomitant administration of atorvastatin (20 to 40 mg) and itraconazole (200 mg) was associated with a 2.5-3.3-fold increase in atorvastatin AUC.

Diltiazem hydrochloride: Co-administration of atorvastatin (40 mg) with diltiazem (240 mg) was associated with higher plasma concentrations of atorvastatin.

Cimetidine: Atorvastatin plasma concentrations and LDL-C reduction were not altered by co-administration of cimetidine.

Grapefruit juice: Contains one or more components that inhibit CYP 3A4 and can increase plasma concentrations of atorvastatin, especially with excessive grapefruit juice consumption (>1.2 liters per day).

Cyclosporine: Atorvastatin and atorvastatin-metabolites are substrates of the OATP1B1 transporter. Inhibitors of the OATP1B1 (e.g. cyclosporine) can increase the bioavailability of atorvastatin. Concomitant administration of atorvastatin 10 mg and cyclosporine 5.2 mg/kg/day resulted in an 8.7-fold increase in atorvastatin AUC. In cases where co-administration of atorvastatin with cyclosporine is necessary, the dose of atorvastatin should not exceed 10 mg (see WARNINGS, Skeletal Muscle).

Inducers of cytochrome P450 3A4 : Concomitant administration of atorvastatin with inducers of cytochrome P450 3A4 (eg efavirenz, rifampin) can lead to variable reductions in plasma concentrations of atorvastatin. Due to the dual interaction mechanism of rifampin, simultaneous co-administration of atorvastatin with rifampin is recommended, as delayed administration of atorvastatin after administration of rifampin has been associated with a significant reduction in atorvastatin plasma concentrations.

Antacid: When atorvastatin and Maalox® TC suspension were coadministered, plasma concentrations of atorvastatin decreased approximately 35%. However, LDL-C reduction was not altered.

Antipyrine: Because atorvastatin does not affect the pharmacokinetics of antipyrine, interactions with other drugs metabolized via the same cytochrome isozymes are not expected.

Colestipol: Plasma concentrations of atorvastatin decreased approximately 25% when colestipol and atorvastatin were coadministered. However, LDL-C reduction was greater when atorvastatin and colestipol were coadministered than when either drug was given alone.

Digoxin: When multiple doses of atorvastatin and digoxin were coadministered, steady-state plasma digoxin concentrations increased by approximately 20%. Patients taking digoxin should be monitored appropriately.

Oral Contraceptives: Coadministration of atorvastatin and an oral contraceptive increased AUC values for norethindrone and ethinyl estradiol by approximately 30% and 20%. These increases should be considered when selecting an oral contraceptive for a woman taking atorvastatin.

Warfarin: Atorvastatin had no clinically significant effect on prothrombin time when administered to patients receiving chronic warfarin treatment.

Amlodipine*:* In a drug-drug interaction study in healthy subjects, co-administration of atorvastatin 80 mg and amlodipine 10 mg resulted in an 18% increase in exposure to atorvastatin which was not clinically meaningful.

Endocrine Function

HMG-CoA reductase inhibitors interfere with cholesterol synthesis and theoretically might blunt adrenal and/or gonadal steroid production. Clinical studies have shown that atorvastatin does not reduce basal plasma cortisol concentration or impair adrenal reserve. The effects of HMG-CoA reductase inhibitors on male fertility have not been studied in adequate numbers of patients. The effects, if any, on the pituitary-gonadal axis in premenopausal women are unknown. Caution should be exercised if an HMG-CoA reductase inhibitor is administered concomitantly with drugs that may decrease the levels or activity of endogenous steroid hormones, such as ketoconazole, spironolactone, and cimetidine.

CNS Toxicity

Brain hemorrhage was seen in a female dog treated for 3 months at 120 mg/kg/day. Brain hemorrhage and optic nerve vacuolation were seen in another female dog that was sacrificed in moribund condition after 11 weeks of escalating doses up to 280 mg/kg/day. The 120 mg/kg dose resulted in a systemic exposure approximately 16 times the human plasma area-under-the-curve (AUC, 0-24 hours) based on the maximum human dose of 80 mg/day. A single tonic convulsion was seen in each of 2 male dogs (one treated at 10 mg/kg/day and one at 120 mg/kg/day) in a 2-year study. No CNS lesions have been observed in mice after chronic treatment for up to 2 years at doses up to 400 mg/kg/day or in rats at doses up to 100 mg/kg/day. These doses were 6 to 11 times (mouse) and 8 to 16 times (rat) the human AUC (0-24) based on the maximum recommended human dose of 80 mg/day.

CNS vascular lesions, characterized by perivascular hemorrhages, edema, and mononuclear cell infiltration of perivascular spaces, have been observed in dogs treated with other members of this class. A chemically similar drug in this class produced optic nerve degeneration (Wallerian degeneration of retinogeniculate fibers) in clinically normal dogs in a dose-dependent fashion at a dose that produced plasma drug levels about 30 times higher than the mean drug level in humans taking the highest recommended dose.

Carcinogenesis, Mutagenesis, Impairment of Fertility

In a 2-year carcinogenicity study in rats at dose levels of 10, 30, and 100 mg/kg/day, 2 rare tumors were found in muscle in high-dose females: in one, there was a rhabdomyosarcoma and, in another, there was a fibrosarcoma. This dose represents a plasma AUC (0-24) value of approximately 16 times the mean human plasma drug exposure after an 80 mg oral dose.

A 2-year carcinogenicity study in mice given 100, 200, or 400 mg/kg/day resulted in a significant increase in liver adenomas in high-dose males and liver carcinomas in high-dose females. These findings occurred at plasma AUC (0-24) values of approximately 6 times the mean human plasma drug exposure after an 80 mg oral dose.

In vitro, atorvastatin was not mutagenic or clastogenic in the following tests with and without metabolic activation: the Ames test with *Salmonella typhimurium* and *Escherichia coli*, the HGPRT forward mutation assay in Chinese hamster lung cells, and the chromosomal aberration assay in Chinese hamster lung cells. Atorvastatin was negative in the *in vivo* mouse micronucleus test.

Studies in rats performed at doses up to 175 mg/kg (15 times the human exposure) produced no changes in fertility. There was aplasia and aspermia in the epididymis of 2 of 10 rats treated with 100 mg/kg/day of atorvastatin for 3 months (16 times the human AUC at the 80 mg dose); testis weights were significantly lower at 30 and 100 mg/kg and epididymal weight was lower at 100 mg/kg. Male rats given 100 mg/kg/day for 11 weeks prior to mating had decreased sperm motility, spermatid head concentration, and increased abnormal sperm. Atorvastatin caused no adverse effects on semen parameters, or reproductive organ histopathology in dogs given doses of 10, 40, or 120 mg/kg for two years.

Pregnancy

Pregnancy Category X

See CONTRAINDICATIONS

Safety in pregnant women has not been established. Atorvastatin crosses the rat placenta and reaches a level in fetal liver equivalent to that of maternal plasma. Atorvastatin was not teratogenic in rats at doses up to 300 mg/kg/day or in rabbits at doses up to 100 mg/kg/day. These doses resulted in multiples of about 30 times (rat) or 20 times (rabbit) the human exposure based on surface area (mg/m^2).

In a study in rats given 20, 100, or 225 mg/kg/day, from gestation day 7 through to lactation day 21 (weaning), there was decreased pup survival at birth, neonate, weaning, and maturity in pups of mothers dosed with 225 mg/kg/day. Body weight was decreased on days 4 and 21 in pups of mothers dosed at 100 mg/kg/day; pup body weight was decreased at birth and at days 4, 21, and 91 at 225 mg/kg/day. Pup development was delayed (rotorod performance at 100 mg/kg/day and acoustic startle at 225 mg/kg/day; pinnae detachment and eye opening at 225 mg/kg/day). These doses correspond to 6 times (100 mg/kg) and 22 times (225 mg/kg) the human AUC at 80 mg/day. Rare reports of congenital anomalies have been received following intrauterine exposure to HMG-CoA reductase inhibitors. There has been one report of severe congenital bony deformity, tracheo-esophageal fistula, and anal atresia (VATER association) in a baby born to a woman who took lovastatin with dextroamphetamine sulfate during the first trimester of pregnancy. LIPITOR should be administered to women of child-bearing potential only when such patients are highly unlikely to conceive and have been informed of the potential hazards. If the woman becomes pregnant while taking LIPITOR, it should be discontinued and the patient advised again as to the potential hazards to the fetus.

Nursing Mothers

Nursing rat pups had plasma and liver drug levels of 50% and 40%, respectively, of that in their mother's milk. Because of the potential for adverse reactions in nursing infants, women taking LIPITOR should not breast-feed (see CONTRAINDICATIONS).

Pediatric Use

Safety and effectiveness in patients 10-17 years of age with heterozygous familial hypercholesterolemia have been evaluated in a controlled clinical trial of 6 months duration in adolescent boys and postmenarchal girls. Patients treated with LIPITOR had an adverse experience profile generally similar to that of patients treated with placebo, the most common adverse experiences observed in both groups, regardless of causality assessment, were infections. **Doses greater than 20 mg have not been studied in this patient population.** In this limited controlled study, there was no detectable effect on growth or sexual maturation in boys or on menstrual cycle length in girls (see CLINICAL PHARMACOLOGY, Clinical Studies section; ADVERSE REACTIONS, Pediatric Patients (ages 10-17 years); and DOSAGE AND ADMINISTRATION, Heterozygous Familial Hypercholesterolemia in Pediatric Patients (10-17 years of age). Adolescent females should be counseled on appropriate contraceptive methods while on LIPITOR therapy (see CONTRAINDICATIONS and PRECAUTIONS, Pregnancy). **LIPITOR has not been studied in controlled clinical trials involving pre-pubertal patients or patients younger than 10 years of age.**

Clinical efficacy with doses up to 80 mg/day for 1 year have been evaluated in an uncontrolled study of patients with homozygous FH including 8 pediatric patients (see CLINICAL PHARMACOLOGY, Clinical Studies: Homozygous Familial Hypercholesterolemia).

Geriatric Use

The safety and efficacy of atorvastatin (10-80 mg) in the geriatric population ($\geq$65 years of age) was evaluated in the ACCESS study. In this 54-week open-label trial 1,958 patients initiated therapy with atorvastatin 10 mg. Of these, 835 were elderly ($\geq$65 years) and 1,123 were non-elderly. The mean change in LDL-C from baseline after 6 weeks of treatment with atorvastatin 10 mg was –38.2% in the elderly patients versus –34.6% in the non-elderly group.

The rates of discontinuation due to adverse events were similar between the two age groups. There were no differences in clinically relevant laboratory abnormalities between the age groups.

Use in Patients with Recent Stroke or TIA

In a post-hoc analysis of the Stroke Prevention by Aggressive Reduction in Cholesterol Levels (SPARCL) study where LIPITOR 80 mg vs placebo was administered in 4,731 subjects without CHD who had a stroke or TIA within the preceding 6 months, a higher incidence of hemorrhagic stroke was seen in the LIPITOR 80 mg group compared to

placebo. Subjects with hemorrhagic stroke on study entry appeared to be at increased risk for hemorrhagic stroke.

ADVERSE REACTIONS

LIPITOR is generally well-tolerated. Adverse reactions have usually been mild and transient. In controlled clinical studies of 2502 patients, <2% of patients were discontinued due to adverse experiences attributable to atorvastatin. The most frequent adverse events thought to be related to atorvastatin were constipation, flatulence, dyspepsia, and abdominal pain.

Clinical Adverse Experiences

Adverse experiences reported in ≥2% of patients in placebo-controlled clinical studies of atorvastatin, regardless of causality assessment, are shown in Table 8.

TABLE 8. Adverse Events in Placebo-Controlled Studies (% of Patients)

BODY SYSTEM/ Adverse Event	Placebo N = 270	Atorvastatin 10 mg N = 863	Atorvastatin 20 mg N = 36	Atorvastatin 40 mg N = 79	Atorvastatin 80 mg N = 94
**BODY AS A WHOLE**					
Infection	10.0	10.3	2.8	10.1	7.4
Headache	7.0	5.4	16.7	2.5	6.4
Accidental Injury	3.7	4.2	0.0	1.3	3.2
Flu Syndrome	1.9	2.2	0.0	2.5	3.2
Abdominal Pain	0.7	2.8	0.0	3.8	2.1
Back Pain	3.0	2.8	0.0	3.8	1.1
Allergic Reaction	2.6	0.9	2.8	1.3	0.0
Asthenia	1.9	2.2	0.0	3.8	0.0
**DIGESTIVE SYSTEM**					
Constipation	1.8	2.1	0.0	2.5	1.1
Diarrhea	1.5	2.7	0.0	3.8	5.3
Dyspepsia	4.1	2.3	2.8	1.3	2.1
Flatulence	3.3	2.1	2.8	1.3	1.1
**RESPIRATORY SYSTEM**					
Sinusitis	2.6	2.8	0.0	2.5	6.4
Pharyngitis	1.5	2.5	0.0	1.3	2.1
**SKIN AND APPENDAGES**					
Rash	0.7	3.9	2.8	3.8	1.1
**MUSCULOSKELETAL SYSTEM**					
Arthralgia	1.5	2.0	0.0	5.1	0.0
Myalgia	1.1	3.2	5.6	1.3	0.0

Anglo-Scandinavian Cardiac Outcomes Trial (ASCOT)
In ASCOT (see CLINICAL PHARMACOLOGY, *Clinical Studies*) involving 10,305 participants treated with LIPITOR 10 mg daily (n=5,168) or placebo (n=5,137), the safety and tolerability profile of the group treated with LIPITOR was comparable to that of the group treated with placebo during a median of 3.3 years of follow-up.

Collaborative Atorvastatin Diabetes Study (CARDS)
In CARDS (see CLINICAL PHARMACOLOGY, *Clinical Studies*) involving 2838 subjects with type 2 diabetes treated with LIPITOR 10 mg daily (n=1428) or placebo (n=1410), there was no difference in the overall frequency of adverse events or serious adverse events between the treatment groups during a median follow-up of 3.9 years. No cases of rhabdomyolysis were reported.

Treating to New Targets Study (TNT)
In TNT (see CLINICAL PHARMACOLOGY, *Clinical Studies*) involving 10,001 subjects with clinically evident CHD treated with LIPITOR 10 mg daily (n=5006) or LIPITOR 80 mg daily (n=4995), there were more serious adverse events and discontinuations due to adverse events in the high-dose atorvastatin group (92, 1.8%; 497, 9.9%, respectively) as compared to the low-dose group (69, 1.4%; 404, 8.1%, respectively) during a median follow-up of 4.9 years. Persistent transaminase elevations (≥3 x ULN twice within 4-10 days) occurred in 62 (1.3%) individuals with atorvastatin 80 mg and in nine (0.2%) individuals with atorvastatin 10 mg. Elevations of CK (≥ 10 x ULN) were low overall, but were higher in the high-dose atorvastatin treatment group (13, 0.3%) compared to the low-dose atorvastatin group (6, 0.1%).

Incremental Decrease in Endpoints Through Aggressive Lipid Lowering Study (IDEAL)
In IDEAL (see CLINICAL PHARMACOLOGY, *Clinical Studies*) involving 8,888 subjects treated with LIPITOR 80 mg/day (n=4439) or simvastatin 20-40 mg daily (n=4449), there was no difference in the overall frequency of adverse events or serious adverse events between the treatment groups during a median follow-up of 4.8 years.

The following adverse events were reported, regardless of causality assessment in patients treated with atorvastatin in clinical trials. The events in italics occurred in ≥2% of patients and the events in plain type occurred in <2% of patients.

Body as a Whole: *Chest pain,* face edema, fever, neck rigidity, malaise, photosensitivity reaction, generalized edema.

Digestive System: *Nausea,* gastroenteritis, liver function tests abnormal, colitis, vomiting, gastritis, dry mouth, rectal hemorrhage, esophagitis, eructation, glossitis, mouth ulceration, anorexia, increased appetite, stomatitis, biliary pain, cheilitis, duodenal ulcer, dysphagia, enteritis, melena, gum hemorrhage, stomach ulcer, tenesmus, ulcerative stomatitis, hepatitis, pancreatitis, cholestatic jaundice.

Respiratory System: *Bronchitis, rhinitis,* pneumonia, dyspnea, asthma, epistaxis.

Nervous System: *Insomnia, dizziness,* paresthesia, somnolence, amnesia, abnormal dreams, libido decreased, emotional lability, incoordination, peripheral neuropathy, torticollis, facial paralysis, hyperkinesia, depression, hypesthesia, hypertonia.

Musculoskeletal System: *Arthritis,* leg cramps, bursitis, tenosynovitis, myasthenia, tendinous contracture, myositis.

Skin and Appendages: Pruritus, contact dermatitis, alopecia, dry skin, sweating, acne, urticaria, eczema, seborrhea, skin ulcer.

Urogenital System: *Urinary tract infection, hematuria, albuminuria,* urinary frequency, cystitis, impotence, dysuria, kidney calculus, nocturia, epididymitis, fibrocystic breast,

vaginal hemorrhage, breast enlargement, metrorrhagia, nephritis, urinary incontinence, urinary retention, urinary urgency, abnormal ejaculation, uterine hemorrhage.

Special Senses: Amblyopia, tinnitus, dry eyes, refraction disorder, eye hemorrhage, deafness, glaucoma, parosmia, taste loss, taste perversion.

Cardiovascular System: Palpitation, vasodilatation, syncope, migraine, postural hypotension, phlebitis, arrhythmia, angina pectoris, hypertension.

Metabolic and Nutritional Disorders: *Peripheral edema*, hyperglycemia, creatine phosphokinase increased, gout, weight gain, hypoglycemia.

Hemic and Lymphatic System: Ecchymosis, anemia, lymphadenopathy, thrombocytopenia, petechia.

Postintroduction Reports

Adverse events associated with LIPITOR therapy reported since market introduction, that are not listed above, regardless of causality assessment, include the following: anaphylaxis, angioneurotic edema, bullous rashes (including erythema multiforme, Stevens-Johnson syndrome, and toxic epidermal necrolysis), rhabdomyolysis, fatigue, and tendon rupture.

Pediatric Patients (ages 10-17 years)
In a 26-week controlled study in boys and postmenarchal girls (n=140), the safety and tolerability profile of LIPITOR 10 to 20 mg daily was generally similar to that of placebo (see CLINICAL PHARMACOLOGY, Clinical Studies section and PRECAUTIONS, Pediatric Use).

OVERDOSAGE

There is no specific treatment for atorvastatin overdosage. In the event of an overdose, the patient should be treated symptomatically, and supportive measures instituted as required. Due to extensive drug binding to plasma proteins, hemodialysis is not expected to significantly enhance atorvastatin clearance.

DOSAGE AND ADMINISTRATION

The patient should be placed on a standard cholesterol-lowering diet before receiving LIPITOR and should continue on this diet during treatment with LIPITOR.

Hypercholesterolemia (Heterozygous Familial and Nonfamilial) and Mixed Dyslipidemia *(Fredrickson* Types IIa and IIb)

The recommended starting dose of LIPITOR is 10 or 20 mg once daily. Patients who require a large reduction in LDL-C (more than 45%) may be started at 40 mg once daily.

The dosage range of LIPITOR is 10 to 80 mg once daily. LIPITOR can be administered as a single dose at any time of the day, with or without food. The starting dose and maintenance doses of LIPITOR should be individualized according to patient characteristics such as goal of therapy and response (see *NCEP Guidelines*, summarized in Table 7). After initiation and/or upon titration of LIPITOR, lipid levels should be analyzed within 2 to 4 weeks and dosage adjusted accordingly.

Since the goal of treatment is to lower LDL-C, the NCEP recommends that LDL-C levels be used to initiate and assess treatment response. Only if LDL-C levels are not available, should total-C be used to monitor therapy.

Heterozygous Familial Hypercholesterolemia in Pediatric Patients (10-17 years of age)
The recommended starting dose of LIPITOR is 10 mg/day; the maximum recommended dose is 20 mg/day (doses greater than 20 mg have not been studied in this patient population). Doses should be individualized according to the recommended goal of therapy (see NCEP Pediatric Panel Guidelines[1], CLINICAL PHARMACOLOGY, and INDICATIONS AND USAGE). Adjustments should be made at intervals of 4 weeks or more.

Homozygous Familial Hypercholesterolemia

The dosage of LIPITOR in patients with homozygous FH is 10 to 80 mg daily. LIPITOR should be used as an adjunct to other lipid-lowering treatments (e.g., LDL apheresis) in these patients or if such treatments are unavailable.

Concomitant Lipid Lowering Therapy

LIPITOR may be used in combination with a bile acid binding resin for additive effect. The combination of HMG-CoA reductase inhibitors and fibrates should generally be avoided (see WARNINGS, Skeletal Muscle, and PRECAUTIONS, Drug Interactions for other drug-drug interactions).

Dosage in Patients With Renal Insufficiency

Renal disease does not affect the plasma concentrations nor LDL-C reduction of atorvastatin; thus, dosage adjustment in patients with renal dysfunction is not necessary (see CLINICAL PHARMACOLOGY, Pharmacokinetics).

Dosage in Patients Taking Cyclosporine, Clarithromycin or A Combination of Ritonavir plus Saquinavir or Lopinavir plus Ritonavir

---

[1] National Cholesterol Education Program (NCEP): Highlights of the Report of the Expert Panel on Blood Cholesterol Levels in Children Adolescents, *Pediatrics.* 89(3):495-501. 1992.

In patients taking cyclosporine, therapy should be limited to LIPITOR 10 mg once daily. In patients taking clarithromycin or in patients with HIV taking a combination of ritonavir plus saquinavir or lopinavir plus ritonavir, for doses of atorvastatin exceeding 20 mg appropriate clinical assessment is recommended to ensure that the lowest dose necessary of atorvastatin is employed (see WARNINGS, Skeletal Muscle, and PRECAUTIONS, Drug Interactions).

HOW SUPPLIED

LIPITOR® (atorvastatin calcium) is supplied as white, elliptical, film-coated tablets of atorvastatin calcium containing 10, 20, 40 and 80 mg atorvastatin.

10 mg tablets: coded “PD 155” on one side and “10” on the other.
NDC 0071-0155-23 bottles of 90
NDC 0071-0155-34 bottles of 5000
NDC 0071-0155-40 10 x 10 unit dose blisters

20 mg tablets: coded “PD 156” on one side and “20” on the other.
NDC 0071-0156-23 bottles of 90
NDC 0071-0156-40 10 x 10 unit dose blisters
NDC 0071-0156-94 bottles of 5000

40 mg tablets: coded “PD 157” on one side and “40” on the other.
NDC 0071-0157-23 bottles of 90
NDC 0071-0157-73 bottles of 500
NDC 0071-0157-88 bottles of 2500
NDC 0071-0157-40 10 x 10 unit dose blisters

80 mg tablets: coded “PD 158” on one side and “80” on the other.
NDC 0071-0158-23 bottles of 90
NDC 0071-0158-73 bottles of 500
NDC 0071-0158-88 bottles of 2500
NDC 0071-0158-92 8 x 8 unit dose blisters

Storage
Store at controlled room temperature 20 - 25˚C (68 - 77˚F) [see USP].

Rx Only

Manufactured by:
Pfizer Ireland Pharmaceuticals
Dublin, Ireland

Distributed by:

Pfizer

Parke-Davis

Division of Pfizer Inc, NY, NY 10017

LAB-0021-20.0 Revised November 2007

Merck & Co., Inc
Code of Conduct for Clinical Trials

I. **Introduction**

A. **Purpose**

Merck & Co., Inc. ("Merck") conducts clinical trials worldwide to evaluate the safety and effectiveness of our products. As such, we are committed to designing, implementing, conducting, analyzing and reporting these studies in compliance with the highest ethical and scientific standards. Protection of patient safety is the overriding concern in the design of clinical trials. In all cases, Merck clinical studies will be consistent with standards established by the Declaration of Helsinki and in compliance with all local and/or national regulations and directives.

B. **Scope**

Such standards shall be endorsed for all clinical interventional investigations sponsored by Merck irrespective of the party (parties) employed for their execution (e.g., contract research organizations, collaborative research efforts). This Code is not intended to apply to studies which are observational in nature, or which are retrospective. Further, this Code does not apply to investigator-initiated studies (e.g., Medical School Grant Program), which are not under the control of Merck.

II. **Scientific Issues**

A. **Study Conduct**

1. **Study Design**

Except for pilot or estimation studies, clinical trial protocols will be hypothesis-driven to assess safety, efficacy and/or pharmacokinetic or pharmacodynamic indices of Merck or comparator products. Alternatively, Merck may conduct outcomes research trials, studies to assess or validate various endpoint measures, or studies to determine patient preferences, etc.

The design and conduct of a study (i.e., patient population, duration, statistical power) must be adequate to address the specific purpose of the study. Research subjects must meet protocol entry criteria to be enrolled in the study, unless specifically exempted by the Merck study monitor.

2. **Site Selection**

Merck selects investigative sites based on medical expertise, access to appropriate patients, adequacy of facilities and staff, previous performance in Merck studies, as well as budgetary considerations. Prior to study initiation, sites are evaluated by Merck personnel to assess the ability to successfully conduct the trial.

3. **Site Monitoring/Scientific Integrity**

Study sites are monitored to assess compliance with the study protocol and general principles of Good Clinical Practice. Merck reviews clinical data for accuracy, completeness and consistency: data are verified versus source documentation according to standard operating procedures. Per Merck policies and procedures, if fraud and/or misconduct are suspected, the issue is investigated: when necessary, the clinical site will be closed and, if appropriate, the responsible regulatory authorities and ethics review committees notified.

B. **Publication and Authorship**

To the extent scientifically appropriate, Merck seeks to publish the results of studies it conducts. Some early phase or pilot studies are intended to be hypothesis-generating rather than hypothesis testing. In such cases, publication of results may not be appropriate since the trial may be underpowered and the analyses complicated by statistical issues of multiplicity.

Merck's policy on authorship is consistent with the requirements outlined in the ICH-Good Clinical Practice guidelines. In summary, authorship should reflect significant contribution to the design and conduct of the study, performance or interpretation of the analysis, and/or writing of the manuscript. All named authors must be able to defend the study results and conclusions. Merck funding of a study will be acknowledged in publications.

III. **Patient Protection**

A. **IRB/ERC review**

All clinical trials will be reviewed and approved by an independent IRB/ERC before being initiated at each site. Significant changes or revisions to the protocol will be approved by the IRB/ERC prior to implementation, except that changes required urgently to protect patient safety and well-being may be enacted in anticipation of IRB/ERC approval. For each site, the IRB/ERC and Merck's Consent Form Review department (U.S. studies) or local medical director (non-U.S. studies) will approve the patient informed consent form.

B. **Safety**

The guiding principle in decision-making in clinical trials is that patient welfare is of primary importance. Potential patients will be informed of the risks and benefits of, as well as alternatives to, study participation. At a minimum, study designs will take into account the local standard of care. Patients are never denied access to appropriate medical care based on participation in a Merck clinical study.

All participation in Merck clinical trials is voluntary. Patients are enrolled only after providing informed consent for participation. Patients may withdraw from a Merck study at any time, without any influence on their access to, or receipt of, medical care that may otherwise be available to them.

C. **Confidentiality**

Merck is committed to safeguarding patient confidentiality, to the greatest extent possible. Unless required by law, only the investigator, sponsor (or representative) and/or regulatory authorities will have access to confidential medical records that might identify the research subject by name.

D. **DNA Research**

DNA sequence analyses, including use of archival specimens collected as part of a clinical trial, will only be performed with the specific informed consent of the subject. With IRB approval, an exception to this restriction on use of archival specimens may be possible (for instance, if specimens are de-identified and are not referable to a specific subject).

IV. **Financial Considerations**

A. **Payments to Investigators**

Clinical trials are time- and labor-intensive. It is Merck's policy to compensate investigators (or the sponsoring institution) in a fair manner for the work performed in support of Merck studies. Merck does not pay incentives to enroll patients in its trials. However, when enrollment is particularly challenging, additional payments may be made to compensate for the time spent in extra recruiting efforts.

Merck does not pay for patient referrals. However, Merck may compensate referring physicians for time spent on chart review to identify potentially eligible patients.

B. **Clinical Research Funding**

Informed consent forms will disclose that the trial is sponsored by Merck, and that the investigator or sponsoring institution is being paid or provided a grant for performing the study. However, the local IRB/ERC may wish to alter the wording of the disclosure statement to be consistent with financial practices at that institution. As noted above, publications resulting from Merck studies will indicate Merck as a source of funding.

C. **Funding for Travel and Other Requests**

Funding of travel by investigators and support staff (e.g. to scientific meetings, investigator meetings, etc.) will be consistent with local guidelines and practices including, in the U.S., those established by the American Medical Association (AMA).

V. **Investigator Commitment**

Investigators will be expected to review Merck's Code of Conduct as an attachment to the study protocol, and in signing the protocol, agree to support these ethical and scientific standards.

Privacy Protection of Pharmacogenomic Sample Collections in Merck & Co., Inc. Clinical Trials
A Guideline for Clinicians and Privacy Board Members

1. **Principles and Introduction**
It is now well recognized that information encoded in genes may affect not only the presence or absence of disease, but also responses to medical treatment. The study of the relationships between genetic variability and drug efficacy is termed *pharmacogenomics*. Merck & Co., Inc. (MERCK) recognizes that pharmacogenomics offers a unique opportunity to enhance our understanding of human disease and ultimately to aid in the discovery of novel, breakthrough medications.
MERCK also recognizes, however, that analyses of samples derived from consenting patients, including pharmacogenomics, must be undertaken with the utmost consideration for human dignity and privacy, as noted in the Declaration of Helsinki, US FDA Requirements (21 CFR 50.20, 50.25, and 50.27), the International Conference on Harmonization (ICH) E6 Good Clinical Practices Guideline, and the 1997 UNESCO Declaration on the Human Genome and Human Rights. This document outlines MERCK's approach to pharmacogenomic analyses.
2. **Definitions**
For the purposes of this document, the following terms will apply:
Pharmacogenomics: The study of how variations in the system of human biomolecules, such as DNA, RNA, and proteins affect individual patient responses to drugs.
Personal Identifiers: Generally defined as data fields that would reasonably allow a third party to identify who a patient is.
Study Site: The local site of the investigation, where patients are actively screened, enrolled and studied as per the clinical protocol.
Dual-Coded Samples/Data are double coded and labeled with the unique second number. The dual code is a random number that is distinct from the allocation number, but has no personal identifiers associated with it. This number is called the Genetic (sample) Identification Code or "GIC". At the beginning of the study, the GIC is associated with allocation numbers in a one-to-one fashion. The link between the clinical study subject number and the unique second number is maintained, but unknown to investigators and patients.
Genetic Identification Code (GIC): A completely random number that is assigned to a sample (see above). No two subjects will have the same GIC within a given study.
Genetic or GIC "Key": The table of uniquely created random numbers (i.e., GICs) associated with the study subject numbers.
Genetic Sample Bank: The third-party entity that is responsible for accessioning, proper handling, and archiving samples from subjects.
3. **Pharmacogenomic Analyses**
Genomic DNA may be extracted from blood samples by standard methods for pharmacogenomic analyses. These DNA samples may be assayed for the presence of genetic variations in genes and blood components that are related to the development and treatment of the diseases studied in MERCK trials. The results of the pharmacogenomic analyses are not directly associated with potentially personally identifying information. The Genetic Key allows the inspection of pharmacogenomic data by regulatory agencies to perform their normal auditing procedures.
4. **Informed Consent**
As per protocol procedures, patients should be presented with the pharmacogenomic analyses informed consent form at a designated visit. The consent should be administered in the standard manner, with special care to explain to the patient how their confidentiality will be maintained. The individual administering consent should also carefully explain that the patient has the option to withdraw their sample at a later date.
Information pertaining to the administration and acquisition of the pharmacogenomic analyses consent will be captured in the Case Report Forms (CRFs). These minimal data will be used by both MERCK and the Genetic Sample Bank to verify that all pharmacogenomics samples were obtained from a patient who gave appropriate consent. Any samples for which a valid informed consent cannot be verified will be promptly destroyed as described below.
5. **Assembly of Kits**
The Genetic Sample Bank will be respons ble for assembling kits for use in the collection of pharmacogenomic samples. These kits will essentially be composed of 1) a 10-mL EDTA collection tube, 2) an appropriate phlebotomy needle, 3) a special requisition form, and 4) a biohazard pouch. These kits will be distributed to the study sites.
6. **Sample Collection**
After administration and obtaining of appropriate informed consent, the study site staff will collect a 10-mL specimen of whole blood by vein into a 10-mL EDTA (lavender top) tube to obtain an anticoagulated specimen. The tube will be inverted 10 times immediately after blood draw to ensure proper anticoagulation.
The study site staff will put the MERCK GIC label on the tube. The tube may or may not have a second label (which is also not personally identifying) according to the specimen management requirements of the Genetic Sample Bank. The study site staff may only write the collection date (date of phlebotomy) and the type of specimen (most commonly "whole blood" or "blood") on the tube label. No other patient information will be applied to the sample label (no patient initials, no birth date, etc.).
7. **Shipping**
The genomic samples (in 10-mL EDTA lavender-top tubes) will be shipped overnight at ambient temperature to the Genetic Sample Bank.
The genomic samples will always travel in a separate pouch with their own accession sheet. The shipment airbill may be preprinted with the study site with contact information or, alternatively, the study site return address may be left blank and filled in at the study site. The final accession number in the Genetic Sample Bank database will not be associated with the investigator name (considered by privacy experts to be a potential personal identifier) or geographic location by using the airbill information.

8. Receipt and Archival of the Pharmacogenomic Specimen

At the Genetic Sample Bank, the 10-mL sample will be routed directly to the appropriate department and accessioned. At the time of receipt, the Genetic Sample Bank will also capture the data fields that travel with the specimen, including collection date and type of specimen. These data are considered to be not personally identifying. The Genetic Sample Bank will also capture as a field in the database the date of specimen receipt and time of receipt. However, investigator information on the airbill will not be recorded in the database, since these may be considered personally identifying. No linkage between the laboratory safety specimens and the pharmacogenomic sample will be maintained by the Genetic Sample Bank.

9. Post-Receipt Processing of Pharmacogenomic Specimen and Storage

The genomic samples will be processed as specified in the contract after receipt by the Genetic Sample Bank, according to the specific pharmacogenomic analyses being performed. MERCK will continuously monitor the disposition of the samples at the Genetic Sample Bank, for example to meet the agreement specified in the consent with respect to sample storage duration. Sample condition will also be carefully monitored so that each sample may be appropriately used for its intended purpose, also as described in the consent document.

The Genetic Sample Bank will track samples using only accession numbers or GICs. Only the above specified fields will be associated with the sample in the Genetic Sample Bank database. No other information about the patient, including study site or geographic location of origination of the sample will be present in the database associated with the sample. Discrepancies/problem vials are to be destroyed and not accessioned, with notation in the database and appropriate notification of MERCK.

10. Sample Destruction Procedures for Withdrawal of Consent

If a patient withdraws consent for the pharmacogenomic analyses portion of the study, any samples taken specifically for pharmacogenomic analyses will be destroyed according to the standard operating procedures of the Genetic Sample Bank where the samples reside. Additionally, if the medical records for the main study are no longer available, for example if the investigator is no longer required by regulatory agencies to retain the main study records, there will no longer be a link between the patient's personal information and their genetic sample. Therefore, the request for sample destruction can not be processed,

Patients that request their samples to be withdrawn are instructed in the consent form to contact the Investigator *in writing*. The Investigator will contact MERCK using the supplied telephone contact (see Sponsor Contact Information section) and a form will be provided by MERCK to obtain appropriate information to complete sample withdrawal. MERCK will identify samples to be destroyed using an agreed upon form provided by the Genetic Sample Bank. After appropriate sign-off by both parties and affirmation of destruction, samples will be retrieved from -70˚C storage and incinerated or pyrolyzed such that DNA and other biomolecules are completely destroyed, i.e. rendered to a state such that the DNA is not able to be manipulated by standard molecular biological techniques (i.e. PCR). A confirmatory letter will be sent from the Genetic Sample Bank to MERCK and then later from MERCK to the investigator.

11. Conclusions

Merck recognizes both the tremendous potential, and the inherent responsibility that pharmacogenomic research provides to clinical studies. The procedures outlined in this document are intended to ensure that meaningful investigation of pharmacogenomics influences in disease and/or responses to therapies can be achieved while providing the highest degree of protection for patients in the study.

8. SIGNATURES

8.1 SPONSOR'S REPRESENTATIVE

TYPED NAME	SIGNATURE	DATE
______________________	______________________	______________________

8.2 INVESTIGATOR

I agree to conduct this clinical study in accordance with the design outlined in this protocol and to abide by all provisions of this protocol (including other manuals and documents referenced from this protocol); deviations from the protocol are acceptable only with a mutually agreed upon protocol amendment. I agree to conduct the study in accordance with generally accepted standards of Good Clinical Practice. I also agree to report all information or data in accordance with the protocol and, in particular, I agree to report any serious adverse experiences as defined in the SAFETY MEASUREMENTS section of this protocol. I also agree to handle all clinical supplies provided by the SPONSOR and collect and handle all clinical specimens in accordance with the protocol. I understand that information that identifies me will be used and disclosed as described in the protocol, and that such information may be transferred to countries that do not have laws protecting such information. Since the information in this protocol and the referenced Investigator's brochure is confidential, I understand that its disclosure to any third parties, other than those involved in approval, supervision, or conduct of the study is prohibited. I will ensure that the necessary precautions are taken to protect such information from loss, inadvertent disclosure, or access by third parties.

TYPED NAME	SIGNATURE	DATE
______________________	______________________	______________________
